# Comprehensive genetic screening of the South African Parkinson’s disease study collection using the NeuroBooster array

**DOI:** 10.64898/2025.12.17.25341881

**Published:** 2025-12-17

**Authors:** Kathryn Step, Lusanda Madula, Nicole Kuznetsov, Elizna Maasdorp, Jonathan Carr, Riaan van Coller, Ignacio F. Mata, Soraya Bardien

**Affiliations:** 1 Division of Molecular Biology and Human Genetics, Faculty of Medicine and Health Sciences, Stellenbosch University, Cape Town, South Africa; 2 South African Medical Research Council Centre for Tuberculosis Research, Stellenbosch University, Cape Town, South Africa; 3 Center for Alzheimer’s and Related Dementias (CARD), National Institute on Aging and National Institute of Neurological Disorders and Stroke, National Institutes of Health, Bethesda, MD, USA.; 4 DataTecnica LLC, Washington, DC 20037, USA; 5 Division of Immunology, Faculty of Medicine and Health Sciences, Stellenbosch University, Cape Town, South Africa; 6 School for Data Science and Computational Thinking, Stellenbosch University, Cape Town, South Africa; 7 Center for Bioinformatics and Computational Biology, Stellenbosch University, Cape Town, South Africa; 8 Division of Neurology, Department of Medicine, Faculty of Medicine and Health Sciences, Stellenbosch University, Cape Town, South Africa; 9 Department of Neurology, Faculty of Health Sciences, University of Pretoria, Pretoria, South Africa; 10 Genome Sciences and Systems Biology, Cleveland Clinic Research, Cleveland Clinic Foundation, Cleveland, United States; 11 South African Medical Research Council/Stellenbosch University Genomics of Brain Disorders Research Unit, Stellenbosch University, Cape Town, South Africa

**Keywords:** Parkinson’s disease, South Africa, pathogenic variants, copy number variants, CNV-Finder, SNCA, PRKN, Multiplex Ligation-dependent Probe Amplification

## Abstract

Parkinson’s disease (PD) is a complex neurodegenerative disorder with a substantial genetic influence. To better characterize the genetic landscape of PD in South Africa, we conducted the largest genetic screening to date for pathogenic single nucleotide and copy number variations (CNVs) using genotyping array data from 689 PD probands. We identified 16 unique missense variants, confirming 15 with Sanger sequencing, in 47 individuals across seven well-established PD genes, with *GBA1* and *PRKN* being most frequent. Also in known PD genes, 18 variants of unknown significance were found. Additionally, CNV analysis using CNV-Finder revealed seven novel CNVs, five in *PRKN* and two in *SNCA*, of which, six were validated with Multiplex Ligation-dependent Probe Amplification. The findings highlight the contribution of both rare variants and structural rearrangements to PD in this underrepresented population. This study underscores the importance of expanding genetic research in African cohorts to improve global understanding of PD etiology.

## Introduction

Parkinson’s disease (PD) is a common neurodegenerative disorder with a disease etiology linked to both environmental and genetic factors.^1^ Understanding the genetic etiology of PD can clarify the biological pathways involved in disease development, inform risk prediction, and improve strategies for disease prevention, diagnosis, and management.^2^

Approximately 5–10% of individuals with PD carry a pathogenic disease variant in a single gene, representing monogenic forms of the disease.^3,4^ In many cases, monogenic forms of the disease are attributed to highly penetrant and rare genetic variants.^2^ Several genes have been linked to PD, particularly monogenic forms, showing either an autosomal dominant or autosomal recessive mode of inheritance. Notably, of these, six genes (*LRRK2*, *PRKN*, *SNCA*, *PINK1*, *PARK7/DJ-1*, and *VPS35*) have been unequivocally linked to monogenic PD.^4^ Variants in *GBA1*, although not monogenic, remain one of the most significant risk factors for PD.^5^ All seven genes are commonly included in PD diagnostic panels^3,6^.

In addition to missense variants and small indels (short insertions or deletions of DNA), the disease etiology can be attributed to genetic structural variants, resulting in a different number of DNA segment copies than normal.^7^ These are known as copy number variations (CNVs) and include deletions, duplications, insertions, and genomic rearrangements. Although CNVs have been shown to play a role in PD pathogenicity,^8^ they are generally rare and observed in a small proportion of affected individuals at any given gene locus.^9^ Notable CNVs in the context of PD commonly include exonic deletions and duplications in *PRKN* and *PINK1* as well as whole gene duplications and triplications of *SNCA*.^10–12^

Although significant progress has been made in understanding the genetic basis of PD, most studies have focused on European populations, leaving the genetic etiology of PD in African populations, including the South African population, largely uncharacterised. Previous PD research in South Africa employed wet-lab screening techniques, including high-resolution melt analysis, Sanger sequencing, and multiplex ligation-dependent probe amplification (MLPA), to detect pathogenic missense variants, indels, and CNVs commonly observed in populations of predominantly European ancestry. However, screening was limited to a small subset of the study collection and revealed that known PD genetic causes are relatively rare in this cohort.^8,13–15^

To address this gap, we performed a comprehensive genetic screening using genotyping array data for the South African PD Study Collection to identify pathogenic variants including CNVs. We further sought to validate these findings using independent techniques.

## Methods

**Figure 1:**
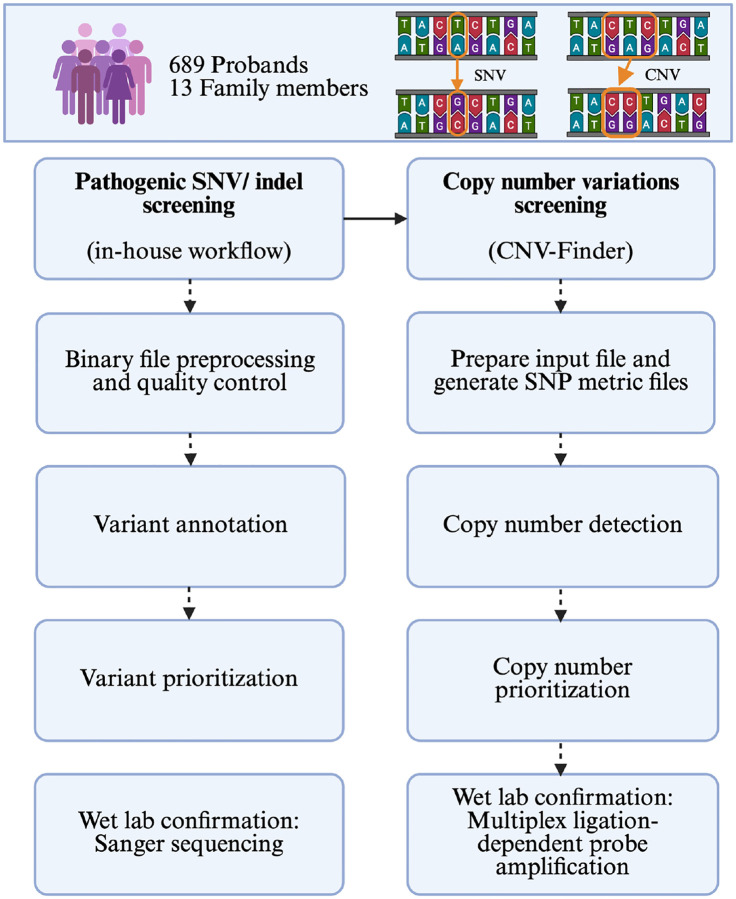
Overview of the study workflow. CNV, copy number variation; SNV, single nucleotide variant.

An overview of the workflow is provided in Figure 1.

### Study participants and genotyping

Study participants were recruited over an 18 year period, from 2002 until June 2020,^16^ as part of the South African PD study collection. Individuals living with PD (n=689 probands; Supplementary Table 1) were diagnosed using the Queen’s Square Brain Bank Criteria.^17^ Ethical clearance was obtained from the Health Research Ethics Committee at Stellenbosch University, South Africa (2002C/059). The study collection was genotyped using the NeuroBooster array (v1.0, Illumina, San Diego, CA) through a collaboration with the Global Parkinson’s Genetics Program (GP2).^18,19^ Previous research has shown the ancestral makeup of the South African PD study collection is 56% European, 18.8% African, 13% indigenous Nama, 6.9% South Asian, and 5.2% Malay.^20^

### Quality control, file preparation, and processing for single nucleotide variant screening

The present study made use of genotyping data. For the pathogenic single nucleotide variant screening, quality control (QC) was performed using PLINK v1.9.^21^ Data missingness was filtered at 10% in individuals and 5% in variants, individuals with sex discrepancies were removed (female F: 0.5; male F: 0.8), variants that deviated from Hardy-Weinberg equilibrium (controls: 1×10^−6^; cases: 1×10^−10^), and those that were more than 3 standard deviations from the mean heterozygosity rate, were removed.

An in-house computational workflow was developed and used to identify and prioritize pathogenic missense variants and indels. Briefly, this consisted of file preparation and conversion from PLINK binary files to variant call format files. Variants were annotated using ANNOVAR v2020-06-08 with all essential databases in Genome Reference Consortium Human Build 38,^22^ including the cytogenetic bands, genome aggregation, nonsynonymous functional prediction, and clinical variant databases. The analysis was performed in two stages. First, the computational workflow was optimized using a subset of probands (n=13) with five pathogenic missense variants previously identified through wet-lab screening conducted by the South African PD research group (Supplementary Table 2). This ensured that the computational workflow could reliably detect known variants. In the second stage, the analysis was restricted to individuals with array genotyping data but without a previously established genetic cause of disease (n=645), allowing for the identification of additional pathogenic missense variants. The ANNOVAR output was aligned to the original files to confirm which individual carried which variant. This was followed by restricting the annotated phenotype to “*Parkinson*” or “*Parkinsonian*”. The files were examined using the following *in-silico* pathogenicity prediction tools and criteria, with variants required to meet at least one of these thresholds to be included: SIFT score < 0.05,^23^ FATHMM score < 0,^24^ PolyPhen-2 score > 0.5,^25^ CADD score >= 20,^26^ MetaLR > 0.8,^27^ and MetaSVM > 0.8.^27^ Finally, variants were filtered using ClinVar (July 2025),^28^ retaining only those classified as “pathogenic”, “likely pathogenic”, or “conflicting pathogenicity” due to their reported associations with the disease. Where DNA from family members was available, co-segregation of the identified pathogenic variants was assessed using Sanger sequencing, as described below. In addition to pathogenic variants, variants of unknown significance (VUS) were also screened using the same workflow. The only modification was adjusting the clinical significance filtering to include variants classified as “uncertain significance” instead of “pathogenic” or “likely pathogenic” according to ClinVar (accessed July 2025).^28^ The prioritized VUS were not confirmed using Sanger sequencing. Additionally, all identified variants in *GBA1* were analysed with GBA1-PD Browser v1.0.^5^

### Validation of single nucleotide variants

The identified putative pathogenic missense variants were confirmed using Sanger sequencing. This included primer design, polymerase chain reaction (PCR) optimization, and Sanger sequencing. The oligonucleotide primers were designed using the Integrated DNA Technologies PrimerQuest^™^ tool and *in-silico* PCR with the University of California Santa Cruz *in-Silico* PCR (https://genome.ucsc.edu/cgi-bin/hgPcr). Detailed information regarding the primers is included in Supplementary Table 3. A gradient PCR was run using the Eppendorf Mastercycler^®^ X50 to optimize the annealing temperatures for the reactions. Following optimization, the PCR reactions were run using the Applied Biosystems GeneAMP^®^ 2700 (Supplementary Table 4 and 5). The PCR products were viewed using 2% agarose gel electrophoresis. Due to the presence of the highly homologous *GBA1LP* pseudogene, the aforementioned protocol was modified when validating the variants in *GBA1*. Here, genomic DNA was amplified using long-range PCR targeting a 7kb fragment, which was viewed using a 0.5% agarose gel and exon-specific primers (nested primers), at a higher-than-normal concentration, were used for Sanger sequencing. All Sanger sequencing was done at the Central Analytical Facilities DNA Sequencing Unit, Stellenbosch University (Supplementary Methods). The DNA chromatograms were analyzed and interpreted using Molecular Evolutionary Genetics Analysis v12.^29^

### File preparation and implementation of CNV-Finder for copy number variations detection

CNV-Finder was applied to the array data using the same workflow as previously described.^30^ Through prior CNV screening, the South African PD Research Group had identified CNVs in 14 probands and six family members (Supplementary Table 6). These previously validated findings were used as reference points to confirm that CNV-Finder was accurately detecting true CNVs in the current dataset. The focus of the screening for the present study was on the probands without a previously identified PD pathogenic variant, including an investigation of potential compound heterozygous CNVs when combined with the single nucleotide variant (SNV) analysis.

Briefly, SNV metric files were created from the genotyped IDAT files as input for the CNV pipeline based on the GenoTools IDAT processing.^31^ For this analysis, the Genome Reference Consortium Human Build 38 was used as a reference genome.^32^ The input SNV metric files were split into interval-based windows to generate sample-level feature matrices for downstream analysis. We applied pretrained Long Short-term Memory models in CNV-Finder, which were trained separately for deletions and duplication, using features based on B Allele Frequency, Log R Ratio, and dosage statistics. For this, deletions were identified using a Log R Ratio of < −0.2, duplications with a Log R Ratio of > 0.2, and insertions with a B Allele Frequency of 0.15–0.35 or 0.65–0.85.^30^ The analysis focused on genes/regions of interest which commonly harbour PD CNVs, including *PARK7/DJ-1*, *PINK1*, *PRKN*, and *SNCA*. This included a default 250kb buffer region for *PARK7/DJ-1*, *PINK1*, and *PRKN* as well as a 1,000kb buffer region for *SNCA* to adequately capture the variations in this gene.^30^ The final output of CNV-Finder includes app-compatible files for visualization using an interactive web application, and results were visually inspected for structural variants.

### Validation of copy number variations

The identified CNVs were confirmed using MPLA. For this, DNA was extracted from a blood sample using established methods. The commercially available SALSA^®^ MLPA^®^ P051 Parkinson Mix-1 kit by MCR Holland (Amsterdam, The Netherlands; http://www.mlpa.com) was used for the MLPA analysis. The kit included probes for *PARK7/DJ-1*, *ATP13A2*, *PINK1*, *PRKN*, and *SNCA*. The analysis was conducted at the Unistel Medical Laboratories in Cape Town, South Africa, according to the manufacturer’s protocol. The MLPA assays were run with positive reference samples (with known CNVs) and wild-type controls, and the results were analysed using the Coffalyser.Net v.250317.1029 software.

## Results

### Bioinformatic analysis confirms the presence of putative pathogenic missense variants

As part of developing and optimizing the pathogenic variant workflow, we confirmed that the workflow was able to detect four previously reported pathogenic variants, which were present in 11 individuals in the South African PD Study Collection. Of the 13 individuals known to carry pathogenic variants, one failed QC due to high missingness, and another, despite passing QC, did not have their pathogenic variant genotyped. This reflects a common limitation of genotyping arrays, which often fail to capture rare pathogenic variants.

When looking at the subset of individuals without a known genetic cause for PD, 637 successfully passed QC. After filtering for PD phenotypes and using *in-silico* pathogenicity prediction tools, 69 variants were identified across nine chromosomes. After further clinical significance filtering to retain variants classified as “pathogenic” or “likely pathogenic”, 16 unique heterozygous variants in 47 individuals were prioritized. These were in the following seven genes: *GBA1* (n=18), *PRKN* (n=14), *LRRK2* (n=5), *PLA2G6* (n=5), *PINK1* (n=3), *POLG* (n=1), and *SYNJ1* (n=1). Sanger sequencing confirmed 15 of the 16 prioritized variants (Table 1). The variant rs191486604 (p.G430D) in *PRKN*, was not validated, suggesting a potential false-positive call in the initial workflow. The GBA1-PD Browser v1.0 classified the two *GBA1* variants as mild risk variants.^5^ Co-segregation analysis was performed for five families, where family member DNA was available. This included the following four variants: rs75548401 (p.T408M) and rs76763715 (p.N409S) in *GBA1* as well as rs199657839 (p.R334C) and rs149953814 (p.P437L) in *PRKN*. Several variants were detected in unaffected relatives, precluding co-segregation with PD. This observation is consistent with *PRKN*, where carriers may be heterozygous while probands are homozygous or compound heterozygous, and for *GBA1*, which is known to exhibit incomplete penetrance. The absence of symptoms among young carriers limits definitive conclusions, underscoring the need for continued clinical follow-up. Additionally, 18 VUS were identified in 27 unrelated cases in the following genes (Supplementary Table 7): *SYNJ1* (n=8), *PINK1* (n=6), *PRKN* (n=4), *GBA1* (n=3), *FBXO7* (n=2), *LRRK2* (n=2), *HTRA2* (n=1), and *PARK7/DJ-1* (n=1). The GBA1-PD Browser v1.0 classified the *GBA1* VUS variants as unknown severity.^5^

### Copy number variant analysis reveals pathogenic structural arrangements

**Figure 2:**
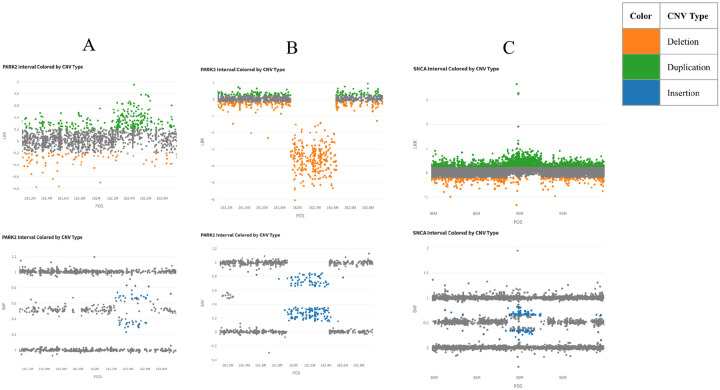
Representative CNV-Finder results showing (A) PRKN duplication, (B) PRKN deletion, and (C) SNCA whole gene duplication. CNVs are determined using visual inspection, prediction values, and the following ranges: Deletions: Log R Ratio (LRR) < −0.2, Duplications: LRR > 0.2, Insertions: 0.15 < B Allele Frequency (BAF) < 0.35 or 0.65 < BAF < 0.85.

The CNV analysis using CNV-Finder identified 18 exonic CNVs in *PRKN* (n=15) and *SNCA* (n=3) in PD probands, along with an additional *SNCA* CNV of interest in the 5’ untranslated region in one proband (Table 2 and Supplementary Table 6). The majority of the CNVs were located in *PRKN*, where ten *PRKN* CNVs were previously identified in probands and two in family members in the study collection.^15,33–35^ The new *PRKN* CNV findings include one duplication and four deletions, all of which were confirmed using MLPA, where three are homozygous. Representative examples of the graphical results from CNV-Finder are provided in Figure 2A and B. Of the 12 exons in *PRKN*, the CNVs identified were localised to exons 2 to 6. Supplementary Figure 1 provides example visualizations of a heterozygous and a homozygous CNV in exon 4 (Supplementary Figure 1). The five cases carrying newly identified *PRKN* CNVs had no PD family history and an age of onset which ranged from 12–45 years.

For *SNCA*, five whole gene multiplications that were previously identified in two families were all confirmed with CNV-Finder. This included three individuals with gene duplications in one family and two individuals with triplications in another.^33,36^ Moreover, one new *SNCA* whole gene duplication was identified using CNV-Finder (Figure 2C) and validated with MLPA. This individual has an age at onset in their 20s and no family history of PD. Additionally, a CNV was identified in the 5’ untranslated region of *SNCA* in one case with an age at onset in their 60s and a family history of PD (Supplementary Figure 2). However, due to the intronic location of this CNV, confirmation with MLPA was not possible.

Detailed clinical information for each novel case is presented in Table 2. We also observed *PRKN* cases with two CNVs that might indicate biallelic variation. These potential pairs were already known from earlier screening efforts, but the second CNV in each case fell below the CNV-Finder prediction score threshold and was excluded, as indicated in Supplementary Table 6. When considering both the pathogenic SNV and CNV analysis, there were no new compound heterozygous variants in the recessive genes detected in the South African PD Study Collection.

## Discussion

This study is the largest genetic screening for PD pathogenic missense and CNVs completed to date for the South African PD Study Collection. We successfully identified 16 missense variants, 18 VUS, and seven CNVs. Additionally, the new findings were confirmed using wet-laboratory approaches including Sanger sequencing and MLPA. The *PRKN* p.G430D variant did not validate with Sanger sequencing, suggesting it may represent a false positive. In contrast, the intronic *SNCA* CNV could not be assessed because the MLPA kit does not include probes covering this region, preventing experimental confirmation.

Integrating the findings from this study with previous screening efforts by the South African PD Research Group, 12.63% of probands (n=87) were found to carry a pathogenic variant across nine genes (Table 3). When considering only confirmed variants, excluding those with incomplete compound heterozygous findings or variants of uncertain penetrance, 3.48% of probands (n=24) harbored a variant that could fully explain their genetic basis for PD. These frequencies are notably lower than those reported in other global cohorts, where pathogenic variants have been detected in upwards of 20% of PD cases, highlighting potential population-specific differences in genetic risk.^1,6,37,38^

In total, the presence of 16 putative pathogenic missense variants were confirmed in 47 individuals with PD. Several well-established PD-associated genes, including *GBA1*, *LRRK2*, *PINK1*, *PRKN*, *PLA2G6*, *POLG*, and *SYNJ1*, were found to harbor variants in our study collection. The most frequent were *GBA1* variants rs75548401 (p.T408M) and rs76763715 (p.N409S), which are known to have higher frequencies in European populations compared to African populations.^39^ Rare variants in *LRRK2* and *PINK1* were also identified, several of which have been proposed as familial causes of PD and are predicted to alter kinase function.^40,41^ Variants in *PRKN*, *PLA2G6*, *POLG*, and *SYNJ1* were observed at low frequencies, with *in-silico* tools supporting pathogenic classifications. This study made use of raw (non-imputed) genotyping data, which likely contributed to the limited number of pathogenic rare variants detected. Our analysis identified 18 VUS, assessed using ClinVar, the clinical implications of which remain to be established. Notably, the majority of these variants have been previously reported to be associated with PD in African, Asian, and European populations,^42–44^ suggesting potential relevance that warrants further investigation. Collectively, these findings highlight genetic contributors to PD in the South African cohort and emphasize the importance of variant characterization in underrepresented populations.

Furthermore, our findings highlight both previously reported and novel CNVs within our study collection. Notably, all five newly identified *PRKN* CNVs carriers presented with early-onset PD (<50 years) (Table 2), consistent with prior reports.^12,45^ All five cases reported no family history for PD. In terms of clinical manifestations, three of the *PRKN* CNV carriers displayed features consistent with *PRKN*-associated PD, albeit with variable presentations. Age at onset ranged from 12 to 45 years. Two presented with dystonia at onset, causing diagnostic delays, including one initially misdiagnosed as dopa-responsive dystonia. Another showed a tremor-dominant phenotype with typical progression, while autonomic symptoms such as urge incontinence and impotence were noted in two cases. Mild neuropsychiatric manifestations, including depression, anxiety, and REM sleep behaviour disorder, occurred in three individuals. Notably, three underwent deep brain stimulation, all with favourable outcomes. Overall, these findings reflect the phenotypic heterogeneity of *PRKN*-associated PD, although individuals still present with the characteristic features of early-onset, good levodopa response, and preserved cognition.^46^

The genomic distribution of *PRKN* CNVs in our dataset further supports observations in literature,^8,45,47^ with most occurring between exons 2 and 6, with exon 4 being the most frequently affected region. Importantly, *PRKN* is well known for harboring compound heterozygous variants, with most PD cases resulting from either two CNVs or a CNV combined with a pathogenic SNV. However, in the present study, no new compound heterozygous cases in *PRKN* were identified. Previous studies have suggested that single CNVs in *PRKN* may increase PD risk,^48,49^ although this association remains inconclusive given contradictory findings in literature.^50,51^ In our dataset, two *PRKN* CNVs were observed as single events, with no accompanying pathogenic SNVs or second CNVs. This suggests that, for those individuals, *PRKN* CNVs did not contribute to PD through the typical biallelic mechanism described in the literature. It remains possible that these individuals carry more complex or hidden structural variants in *PRKN* that are not detectable with genotyping array data. Recent long-read sequencing studies have demonstrated that such approaches can resolve previously unexplained single-heterozygous *PRKN* cases by uncovering intricate structural alternations.^52^ However, in three probands, we identified and validated homozygous *PRKN* CNVs, providing strong evidence that these biallelic events are the genetic cause of their PD.

For *SNCA*, seven CNVs were identified in probands and family members with two being novel findings (Table 2). The individual with the gene duplication showed an early age at onset and no family history of PD. While CNVs in *SNCA* tend to follow an autosomal dominant inheritance pattern,^9^ this type of CNV has been observed in both familial and sporadic PD cases.^53,54^ This individual experienced a range of non-motor symptoms including muscle spasms, loss of smell, sleep disturbances, and constipation. Additionally, motor symptoms included resting tremor which started on the right side and moved to the left, weakened hand strength, as well as poor balance and coordination. The presence of the *SNCA* CNV provides evidence that this is likely the genetic cause of PD for this proband. Finally, they also reported the onset and progression of short term memory loss. While whole gene multiplications are the most common CNV for *SNCA*, duplications are more frequently observed in sporadic PD cases with less severe disease symptoms in comparison to triplications.^55,56^ The *SNCA* CNV identified in the 5’ untranslated region is of particular interest, as disruptions in this region may affect gene expression by altering regulatory elements and transcription factor binding.^51,57^ This individual has late-onset PD and a positive family history, with an affected maternal grandmother. No further clinical information is available as this individual was lost to follow-up. Given the limitations of array-based CNV calling, the presence of this variant would ideally require confirmation with independent validation methods; however, this was not possible as the CNV lies outside the *SNCA* coding region, which is not covered by MLPA.

The reported presence of PD-associated CNVs in individuals of African ancestry, particularly from sub-Saharan Africa, remains limited, although it is unclear whether this reflects small sample size, true rarity, or both. Consistent with this uncertainty, previous screening efforts identified only five probands with *PRKN* CNVs and no *SNCA* CNVs reported in individuals of African ancestry.^8^ Similarly, a recent study successfully identified *PRKN* CNVs in individuals with African and African admixed ancestry on a global scale, but with no *SNCA* CNVs detected.^58^ However, the reported frequency of *PRKN* CNVs in that study was 0.7% of cases, highlighting the low frequency of CNVs in African populations compared to other ancestral groups, where frequencies as high as 10.8% have been reported.^59^ Taken together, this available literature suggests low observed CNV frequencies in African populations and suggests that novel genetic contributors remain to be uncovered.

In our cohort, several individuals carried heterozygous *PINK1* or *PRKN* variants (either SNVs or CNVs) that, on their own, do not fulfil the expected autosomal recessive inheritance pattern typically associated with pathogenicity in these genes.^46,60^ Despite comprehensive screening using both SNV and CNV analyses, we did not identify second hits, for example additional coding variants or copy-number events. In the absence of confirmed compound heterozygosity or biallelic disruption, these cases remain unresolved. It is therefore plausible that some individuals may harbor additional pathogenic alterations that were not detectable with our current array-based CNV approaches.^52^

While a number of pathogenic variants and CNVs were prioritized in the analysis, the study has several limitations. Genotyping arrays are widely used for their cost-effectiveness and coverage of common variants, however, they have limited sensitivity for rare variant detection and do not capture novel pathogenic variants.^61^ Additionally, probe sensitivity varies across genotyping arrays, which can lead to the detection of false positives and false negatives, highlighting the importance of further validation. Moreover, the detection of true CNVs using genotyping arrays remains challenging. While various bioinformatics approaches are available for this analysis, there is considerable variability in these methods.^62^ Some compound heterozygous CNVs, particularly in *PRKN*, were missed because one of the CNVs in the pair received a low CNV-Finder prediction value and was therefore excluded. Furthermore, potential compound heterozygous CNVs occurring in trans across consecutive exons may also be missed without long-read sequencing or parental testing to resolve allele phase. This indicates a limitation of this tool currently and requires further refinement. The results from CNV detection must therefore be carefully evaluated, either by integrating multiple tools or through wet-lab confirmation. While this is possible for genes that are on MLPA kits, not all CNVs can be validated with this approach. Ultimately, long-read whole-genome sequencing or array comparative genomic hybridisation is required to confirm novel CNVs and complex structural arrangements, such as inversions, in this population. This includes variants in non-coding regulatory regions, such as the 5’ untranslated region of *SNCA*, which cannot be validated using MLPA, where preliminary evidence suggests this CNV may represent a true finding.

In conclusion, this study is the first large-scale, systematic SNV and CNV screening in Southern Africa. We identified and prioritized several pathogenic missense variants and CNVs, providing valuable insights into the genetic architecture of the disease in the South African population. While whole genome sequencing is increasing in popularity, the scalability and affordability of genotyping arrays secure their continued role in research contexts. Ultimately, ensuring that researchers maximize the utility of genotyping data is vital for large-scale pathogenic screening, especially in underrepresented populations where resources are limited. Notably, CNV-Finder performed well in our dataset, with MLPA confirming all prioritised CNVs, highlighting its utility as a rapid and cost-effective alternative to MLPA for large-scale CNV detection. Importantly, through our collaboration with the Global Parkinson’s Genetics Program (GP2), WGS data for our study collection will become available in the future, enabling a more comprehensive investigation of pathogenic SNVs and CNVs that may have been missed due to the inherent limitations of array-based analyses. Importantly, these findings indicate the limited number of pathogenic variants detected in the known PD genes. Consequently, this study provides an important foundation for future studies and highlights the need to include African populations in neurological disease research to advance our understanding of disease etiology.

## Supplementary Material

Supplement 1

## Figures and Tables

**Table 1:** Single nucleotide variants identified with the computational workflow which are classified as pathogenic, likely pathogenic, or conflicting pathogenicity.

GP2 sample ID/our lab ID	Gene	Variant (Amino acid change/ rsID)	Family history of PD	EOPD/LOPD
000163/ 10.111	*GBA1*	p.T408M / rs75548401	No	EOPD
000286/ 95.41	*GBA1*	p.T408M / rs75548401	No	LOPD
000339/ 96.91	*GBA1*	p.T408M / rs75548401	Yes	LOPD
000355/ 97.37	*GBA1*	p.T408M / rs75548401	No	LOPD
000510/ 12.797	*GBA1*	p.T408M / rs75548401	Yes	LOPD
000633/ 13.509	*GBA1*	p.T408M / rs75548401	Yes	LOPD
000066/ 67.7	*GBA1*	p.T408M / rs75548401	No	LOPD
000113/ 81.61	*GBA1*	p.T408M / rs75548401	No	LOPD
000133/ 82.45	*GBA1*	p.N409S / rs76763715	No	LOPD
000176/ 83.41	*GBA1*	p.N409S / rs76763715	No	EOPD
000206/ 88.29	*GBA1*	p.N409S / rs76763715	No	LOPD
000357/ 97.43	*GBA1*	p.N409S / rs76763715	Yes	LOPD
000417/ 10.327	*GBA1*	p.N409S / rs76763715	No	LOPD
000427/ 11.59	*GBA1*	p.N409S / rs76763715	No	LOPD
000580/ 13.266	*GBA1*	p.N409S / rs76763715	No	EOPD
000599/ 13.352	*GBA1*	p.N409S / rs76763715	Yes	LOPD
000273/ 12.87	*GBA1*	p.N409S / rs76763715	No	EOPD
000146/ 82.91	*GBA1*	p.N409S / rs76763715	No	LOPD
000437/ 12.705	*LRKK2*	p.L119P / rs33995463	No	LOPD
000195/ 99.48	*LRKK2*	p.R793M / rs35173587	No	LOPD
000366/ 98.33	*LRKK2*	p.R793M / rs35173587	No	LOPD
000154/ 83.00	*LRKK2*	p.R793M / rs35173587	No	LOPD
000545/ 12.925	*LRKK2*	p.R1325Q / rs72546338	No	LOPD
000293/ 95.66	*PINK1*	p.A339T / rs55831733	No	LOPD
000020/ 55.45	*PINK1*	p.A359T / rs76753586	No	LOPD
000052/ 65.68	*PINK1*	p.A494V / rs542258150	No	LOPD
000107/ 81.06	*PINK1*	p.A494V / rs542258150	No	LOPD
000248/ 94.58	*PLA2G6*	p.S34L / rs147948449	Yes	LOPD
000458/ 12.061	*PLA2G6*	p.S34L / rs147948449	No	LOPD
000623/ 13.47	*PLA2G6*	p.D31N / rs150024227	No	LOPD
000050/ 65.26	*PLA2G6*	p.G361S / rs143826762	No	LOPD
000074/ 11.849	*PLA2G6*	p.G361S / rs143826762	No	LOPD
000236/ 92.97	*POLG*	p.V1044A / rs150233690	No	LOPD
000289/ 95.44	*PRKN*	p.R334C / rs199657839	No	LOPD
000194/ 84.3	*PRKN*	p.R334C / rs199657839	No	LOPD
000628/ 13.497	*PRKN*	p.R334C / rs199657839	No	EOPD
000405/ 10.306	*PRKN*	p.R334C / rs199657839	No	EOPD
000552/ 12.964	*PRKN*	p.R334C / rs199657839	No	EOPD
000578/ 13.228	*PRKN*	p.R334C / rs199657839	No	LOPD
000582/ 13.268	*PRKN*	p.R334C / rs199657839	No	LOPD
000616/ 13.429	*PRKN*	p.R402C / rs55830907	No	EOPD
000375/ 99.49	*PRKN*	p.R402C / rs55830907	Yes	LOPD
000478/ 12.242	*PRKN*	p.R402C / rs55830907	No	EOPD
000195/ 99.48	*PRKN*	p.R402C / rs55830907	No	LOPD
000447/ 11.833	*PRKN*	p.P437L / rs149953814	No	LOPD
000050/ 65.26	*PRKN*	p.P437L / rs149953814	No	LOPD
000060/ 12.858	*SYNJ1*	p.I707T / rs147929290	Yes	LOPD

This table provides the variants of interest classified as “pathogenic”, “likely pathogenic”, or “conflicting pathogenicity” as of July 2025 defined by ClinVar. EOPD, early-onset PD; LOPD, late-onset PD; PD, Parkinson’s disease.

**Table 2:** Novel prioritized copy number variations identified in the present study

GP2 ID/our lab ID	Variant	CNV-Finder prediction value	EOPD/LOPD	Family history of PD	Clinical features				
					Pre-motor symptoms	Initial motor symptoms	Prominent motor features	Prominent non-motor symptoms	Treatment note
***PRKN* CNVs**									
000478/12.242	Exon 2 Het DUP	1	EOPD	No	None reported	Head and hand tremor	Freezing of gait	None	DBS for motor fluctuations
000399/10.270	Exons 3–6 Hom DEL	1	EOPD	No	Clinical details unavailable (lost to follow-up).				
000528/12.872	Exon 4 Hom DEL	1	EOPD	No	None reported	Leg tremor	Tremor, off-treatme nt dystonia	Urge-incontinence	Levodopa induced motor complications
000504/12.729	Exon 4 Het DEL	0.9604	EOPD	No	None reported	Dystonia	Dystonia, gait disorder	Autonomic symptoms (urge incontinence, impotence). Marked neuropsychiatric features including insomnia, depression, anxiety, and REM sleep behavior disorder. Normal cognition	DBS for motor complications
000648/13.582	Exon 2–3 Hom DEL	1	EOPD	No	None reported	Dystonia	Abnormal eye movement, poor gait and balance with frequent falls	Mild neuropsychiatric symptoms. Normal cognition	Underwent pallidal DBS for dystonia, later revised to STN with improved outcome
***SNCA* CNVs**									
000493/12.495	Gene Duplication	1	EOPD	No	Muscle spasms, hyposmia, constipation, and REM sleep disturbance	Tremor	Gait and balance impairment	Dysarthria, sialorrhea mild cognitive impairment	
000289/95.44	5’ UTR Duplication	1	LOPD	Yes	Clinical details unavailable (lost to follow-up).				

The CNV-Finder prediction value represents the confidence score for each identified CNV, with values closer to 1 indicating higher confidence in the CNV call. All individuals are PD probands. AAO, age at onset; CNV, Copy number variations; DBS, deep brain stimulation; DEL, deletion; DUP, duplication; EOPD, early-onset PD; Het, Heterozygous; Hom, Homozygous; LOPD, late-onset PD; MLPA, multiplex ligation-dependent probe amplification; *PRKN*, Parkin; *SNCA*, alpha-synuclein; STN, subthalamic nuclei.

**Table 3:** Overview of the variants identified in study collection probands

Gene	Proband carriers, n (%)	Family history of PD, n (%)	EOPD, n (%)	Probands explained, n (%)
*PARK7/DJ-1*	1 (0.15)	0 (0)	1 (0.15)	0 (0)
*GBA1*	22 (3.19)	6 (0.87)	7 (1.02)	0 (0)
*LRRK2*	14 (2.03)	6 (0.87)	3 (0.44)	9 (1.31)
*PRKN*	34 (4.93)	13 (1.89)	19 (2.76)	11 (1.6)
*PINK1*	5 (0.73)	1 (0.15)	1 (0.15)	1 (0.15)
*PLA2G6*	5 (0.73)	1 (0.15)	0 (0)	0 (0)
*POLG*	1 (0.15)	0 (0)	0 (0)	0 (0)
*SYNJ1*	1 (0.15)	1 (0.15)	0 (0)	0 (0)
*SNCA*	4 (0.58)	3 (0.44)	3 (0.44)	3 (0.44)

This table provides a summary of all genetic variants associated with Parkinson’s disease identified in the South African PD study collection to date, comprising 689 probands. ‘Proband carriers, n (%)’ denotes the percentage of probands carrying a pathogenic, likely pathogenic, or variant of uncertain significance (VUS), combining results presented in this manuscript with findings from previous screening initiatives. Percentages for family history and EOPD correspond specifically to this group of proband carriers. ‘Probands explained, n (%)’ refers to the proportion of probands for whom a definitive genetic cause of PD has been established, and includes only confirmed pathogenic or likely pathogenic variants. This category excludes VUS, as well as incomplete compound heterozygous findings and variants with unknown penetrance.

## Data Availability

The data was obtained from the Global Parkinson’s Genetics Program (GP2) and access can be requested through the Accelerating Medicines Partnership in Parkinson’s Disease (AMP-PD) via the online application process (https://www.amp-pd.org/). This analysis made use of CNV-Finder https://github.com/GP2code/CNV-Finder; DOI 10.5281/zenodo.14182563. Additionally, an overview of the analysis and any additional scripts can be found in the GP2 public domain on GitHub (https://github.com/GP2code/PD_genescreen_SouthAfr; DOI 10.5281/zenodo.17478293).

## References

[1] Funayama M., Nishioka K., Li Y. & Hattori N. Molecular genetics of Parkinson’s disease: Contributions and global trends. J. Hum. Genet. 68, 125–130 (2023). 35821405 10.1038/s10038-022-01058-5 PMC9968657

[2] Blauwendraat C., Nalls M. A. & Singleton A. B. The genetic architecture of Parkinson’s disease. Lancet Neurol. 19, 170–178 (2020). 31521533 10.1016/S1474-4422(19)30287-X PMC8972299

[3] Trevisan L. Genetics in Parkinson’s disease, state-of-the-art and future perspectives. Br. Med. Bull. 149, 60–71 (2024). 38282031 10.1093/bmb/ldad035 PMC10938543

[4] Jia F., Fellner A. & Kumar K. R. Monogenic Parkinson’s Disease: Genotype, Phenotype, Pathophysiology, and Genetic Testing. Genes 13, (2022). 10.3390/genes13030471 PMC8950888 35328025

[5] Parlar S. C., Grenn F. P., Kim J. J., Baluwendraat C. & Gan-Or Z. Classification of GBA1 variants in Parkinson’s disease: The GBA1-PD browser. Mov. Disord. 38, 489–495 (2023). 36598340 10.1002/mds.29314 PMC10033371

[6] Cook L. Parkinson’s disease variant detection and disclosure: PD GENEration, a North American study. Brain 147, 2668–2679 (2024). 39074992 10.1093/brain/awae142 PMC11292896

[7] Hastings P. J., Lupski J. R., Rosenberg S. M. & Ira G. Mechanisms of change in gene copy number. Nat. Rev. Genet. 10, 551–564 (2009). 19597530 10.1038/nrg2593 PMC2864001

[8] Müller-Nedebock A. C. Copy number variation in Parkinson’s disease: An update from sub-Saharan Africa. Mov. Disord. 36, 2442–2444 (2021). 34228376 10.1002/mds.28710 PMC8530875

[9] Toft M. & Ross O. A. Copy number variation in Parkinson’s disease. Genome Med. 2, 62 (2010). 20828427 10.1186/gm183 PMC3092113

[10] Singleton A. B. alpha-Synuclein locus triplication causes Parkinson’s disease. Science 302, 841 (2003). 14593171 10.1126/science.1090278

[11] Bardien S. Genetic screening of South African families with Parkinson’s disease. S. Afr. Med. J. 114, e1750 (2024). 38525571 10.7196/SAMJ.2024.v114i2.1750

[12] Landoulsi Z. Large-scale copy number variant analysis in genes linked to Parkinsońs disease. NPJ Parkinsons Dis. 11, 225 (2025). 40750593 10.1038/s41531-025-01076-y PMC12316845

[13] Williams U., Bandmann O. & Walker R. Parkinson’s disease in Sub-Saharan Africa: A review of epidemiology, genetics and access to care. J. Mov. Disord. 11, 53–64 (2018). 29860783 10.14802/jmd.17028 PMC5990907

[14] du Toit N., van Coller R., Anderson D. G., Carr J. & Bardien S. Frequency of the LRRK2 G2019S mutation in South African patients with Parkinson’s disease. Neurogenetics 20, 215–218 (2019). 31493133 10.1007/s10048-019-00588-z

[15] Haylett W. L. Mutations in the parkin gene are a minor cause of Parkinson’s disease in the South African population. Parkinsonism Relat. Disord. 18, 89–92 (2012). 21996382 10.1016/j.parkreldis.2011.09.022

[16] van Rensburg Z. J. The South African Parkinson’s Disease Study Collection. Mov. Disord. 37, 230–232 (2022). 34676912 10.1002/mds.28828

[17] Hughes A. J., Daniel S. E., Kilford L. & Lees A. J. Accuracy of clinical diagnosis of idiopathic Parkinson’s disease: a clinico-pathological study of 100 cases. J. Neurol. Neurosurg. Psychiatry 55, 181–184 (1992). 1564476 10.1136/jnnp.55.3.181 PMC1014720

[18] Global Parkinson’s Genetics Program. GP2: The global Parkinson’s genetics program. Mov. Disord. 36, 842–851 (2021). 33513272 10.1002/mds.28494 PMC9290711

[19] Step K. Advancing Parkinson’s disease research in Africa: A strategic training framework of the global Parkinson’s genetics program. Mov. Disord. 40, 51–56 (2025). 39482233 10.1002/mds.30051 PMC11752974

[20] Step K. Genome-wide association analyses reveal susceptibility variants linked to Parkinson’s disease in the South African population using inferred global and local ancestry. medRxiv (2025) doi:10.1101/2025.08.01.25331910.

[21] Purcell S. PLINK: a tool set for whole-genome association and population-based linkage analyses. Am. J. Hum. Genet. 81, 559–575 (2007). 17701901 10.1086/519795 PMC1950838

[22] Wang K., Li M. & Hakonarson H. ANNOVAR: functional annotation of genetic variants from high-throughput sequencing data. Nucleic Acids Res. 38, e164 (2010). 20601685 10.1093/nar/gkq603 PMC2938201

[23] Ng P. C. & Henikoff S. SIFT: Predicting amino acid changes that affect protein function. Nucleic Acids Res. 31, 3812–3814 (2003). 12824425 10.1093/nar/gkg509 PMC168916

[24] Rogers M. F. FATHMM-XF: accurate prediction of pathogenic point mutations via extended features. Bioinformatics 34, 511–513 (2018). 28968714 10.1093/bioinformatics/btx536 PMC5860356

[25] Adzhubei I., Jordan D. M. & Sunyaev S. R. Predicting functional effect of human missense mutations using PolyPhen-2. Curr. Protoc. Hum. Genet. Chapter 7, Unit 7.20 (2013). 10.1002/0471142905.hg0720s76 PMC4480630 23315928

[26] Rentzsch P., Witten D., Cooper G. M., Shendure J. & Kircher M. CADD: predicting the deleteriousness of variants throughout the human genome. Nucleic Acids Res. 47, D886–D894 (2019). 30371827 10.1093/nar/gky1016 PMC6323892

[27] Dong C. Comparison and integration of deleteriousness prediction methods for nonsynonymous SNVs in whole exome sequencing studies. Hum. Mol. Genet. 24, 2125–2137 (2015). 25552646 10.1093/hmg/ddu733 PMC4375422

[28] Landrum M. J. ClinVar: public archive of relationships among sequence variation and human phenotype. Nucleic Acids Res. 42, D980–5 (2014). 24234437 10.1093/nar/gkt1113 PMC3965032

[29] Kumar S. MEGA12: Molecular evolutionary genetic analysis version 12 for adaptive and green computing. Mol. Biol. Evol. 41, (2024). 10.1093/molbev/msae263 PMC11683415 39708372

[30] Kuznetsov N. CNV-Finder: Streamlining copy number variation discovery. bioRxivorg (2024) doi:10.1101/2024.11.22.624040. PMC13503034 42639318

[31] Vitale D. GenoTools: an open-source Python package for efficient genotype data quality control and analysis. G3 (Bethesda) 15, (2025). 10.1093/g3journal/jkae268 PMC11708233 39566101

[32] Guo Y. Improvements and impacts of GRCh38 human reference on high throughput sequencing data analysis. Genomics 109, 83–90 (2017). 28131802 10.1016/j.ygeno.2017.01.005

[33] Keyser R. J., Lombard D., Veikondis R., Carr J. & Bardien S. Analysis of exon dosage using MLPA in South African Parkinson’s disease patients. Neurogenetics 11, 305–312 (2010). 20013014 10.1007/s10048-009-0229-6

[34] Mahne A. C., Carr J. A., Bardien S. & Schutte C. M. Clinical findings and genetic screening for copy number variation mutations in a cohort of South African patients with Parkinson’s disease. S. Afr. Med. J. 106, 623 (2016). 10.7196/SAMJ.2016.v106i6.10340 27245731

[35] van der Merwe C., Carr J., Glanzmann B. & Bardien S. Exonic rearrangements in the known Parkinson’s disease-causing genes are a rare cause of the disease in South African patients. Neurosci. Lett. 619, 168–171 (2016). 27001088 10.1016/j.neulet.2016.03.028

[36] Braun A. Deep brain stimulation improves symptoms in an individual with alpha-synuclein-gene-associated Parkinson’s disease. Mov. Disord. Clin. Pract. 12, 1200–1203 (2025). 40156366 10.1002/mdc3.70057 PMC12371441

[37] Lim S.-Y. Uncovering the genetic basis of Parkinson’s disease globally: from discoveries to the clinic. Lancet Neurol. 23, 1267–1280 (2024). 39447588 10.1016/S1474-4422(24)00378-8

[38] Bouhouche A. LRRK2 G2019S mutation: Prevalence and clinical features in Moroccans with Parkinson’s disease. Parkinsons Dis. 2017, 2412486 (2017). 28465860 10.1155/2017/2412486 PMC5390546

[39] Karczewski K. J. The mutational constraint spectrum quantified from variation in 141,456 humans. Nature 581, 434–443 (2020). 32461654 10.1038/s41586-020-2308-7 PMC7334197

[40] Vela-Desojo L. A new LRRK2 variant in a family with Parkinson’s disease affects binding to RAB8A. NPJ Parkinsons Dis. 11, 154 (2025). 40483312 10.1038/s41531-025-00989-y PMC12145433

[41] Parlar S. C. LRRK2 rare-variant per-domain genetic burden in Parkinson’s Disease: association confined to the kinase domain. NPJ Parkinsons Dis. 11, 102 (2025). 40301370 10.1038/s41531-025-00934-z PMC12041573

[42] Rizig M. Identification of genetic risk loci and causal insights associated with Parkinson’s disease in African and African admixed populations: a genome-wide association study. Lancet Neurol. 22, 1015–1025 (2023). 37633302 10.1016/S1474-4422(23)00283-1 PMC10593199

[43] Wang C.-Y. Genetic variations of Omi/HTRA2 in Chinese patients with Parkinson’s disease. Brain Res. 1385, 293–297 (2011). 21338583 10.1016/j.brainres.2011.02.037

[44] Junker J. Team science approaches to unravel monogenic Parkinson’s disease on a global scale. Mov. Disord. 39, 1868–1873 (2024). 39076159 10.1002/mds.29925 PMC12131278

[45] Hedrich K. Distribution, type, and origin of Parkin mutations: review and case studies. Mov. Disord. 19, 1146–1157 (2004). 15390068 10.1002/mds.20234

[46] Menon P. J. Genotype-phenotype correlation in PRKN-associated Parkinson’s disease. NPJ Parkinsons Dis. 10, 72 (2024). 38553467 10.1038/s41531-024-00677-3 PMC10980707

[47] Guo J.-F. Exon dosage analysis of parkin gene in Chinese sporadic Parkinson’s disease. Neurosci. Lett. 604, 47–51 (2015). 26240990 10.1016/j.neulet.2015.07.046

[48] Huttenlocher J. Heterozygote carriers for CNVs in PARK2 are at increased risk of Parkinson’s disease. Hum. Mol. Genet. 24, 5637–5643 (2015). 26188007 10.1093/hmg/ddv277

[49] Pankratz N. Copy number variation in familial Parkinson disease. PLoS One 6, e20988 (2011). 21829596 10.1371/journal.pone.0020988 PMC3149037

[50] Wang L. High-resolution survey in familial Parkinson disease genes reveals multiple independent copy number variation events in PARK2. Hum. Mutat. 34, 1071–1074 (2013). 23616242 10.1002/humu.22344 PMC4464794

[51] La Cognata V., Morello G., D’Agata V. & Cavallaro S. Copy number variability in Parkinson’s disease: assembling the puzzle through a systems biology approach. Hum. Genet. 136, 13–37 (2017). 27896429 10.1007/s00439-016-1749-4 PMC5214768

[52] Daida K. The utility of long-read sequencing in diagnosing early onset Parkinson’s disease. Ann. Neurol. 97, 753–765 (2025). 39699073 10.1002/ana.27155 PMC11889530

[53] Duan R.-N. Characterization of SNCA multiplication in Parkinson’s disease: 2 new cases and evaluation of the literature. Mov. Disord. Clin. Pract. 10, 1536–1541 (2023). 37868923 10.1002/mdc3.13852 PMC10585967

[54] Chartier-Harlin M.-C. Alpha-synuclein locus duplication as a cause of familial Parkinson’s disease. Lancet 364, 1167–1169 (2004). 15451224 10.1016/S0140-6736(04)17103-1

[55] Farrer M. Comparison of kindreds with parkinsonism and alpha-synuclein genomic multiplications. Ann. Neurol. 55, 174–179 (2004). 14755720 10.1002/ana.10846

[56] Ross O. A. Genomic investigation of alpha-synuclein multiplication and parkinsonism. Ann. Neurol. 63, 743–750 (2008). 18571778 10.1002/ana.21380 PMC3850281

[57] Gamazon E. R. & Stranger B. E. The impact of human copy number variation on gene expression. Brief. Funct. Genomics 14, 352–357 (2015). 25922366 10.1093/bfgp/elv017 PMC4592354

[58] Akçimen F. Large-scale genetic characterization of Parkinson’s disease in the African and African admixed populations. Brain (2025) doi:10.1093/brain/awaf379. PMC13140531 41058593

[59] Darvish H. Detection of copy number changes in genes associated with Parkinson’s disease in Iranian patients. Neurosci. Lett. 551, 75–78 (2013). 23880019 10.1016/j.neulet.2013.07.013

[60] Krohn L. Comprehensive assessment of PINK1 variants in Parkinson’s disease. Neurobiol. Aging 91, 168.e1–168.e5 (2020). 10.1016/j.neurobiolaging.2020.03.003 PMC7236133 32249012

[61] Quick C. Sequencing and imputation in GWAS: Cost-effective strategies to increase power and genomic coverage across diverse populations. Genet. Epidemiol. 44, 537–549 (2020). 32519380 10.1002/gepi.22326 PMC7449570

[62] van Baardwijk M. N., Heijnen L. S. E. M., Zhao H., Baudis M. & Stubbs A. P. A systematic benchmark of copy number variation detection tools for high density SNP genotyping arrays. Genomics 116, 110962 (2024). 39547585 10.1016/j.ygeno.2024.110962

