# Supplementary material for: Comprehensive genetic screening of the South African Parkinson’s disease study collection using the NeuroBooster array
Source: medRxiv. 2025 Dec 17:2025.12.17.25341881. Preprint. [Version 1] doi: 10.64898/2025.12.17.25341881 (PMC12723977; doi:10.64898/2025.12.17.25341881)
Supplement: Supplement 1 [file media-1.pdf]

Comprehensive genetic screening of the South African Parkinson's disease study collection using the NeuroBooster array

Kathryn Step[#] and Lusanda Madula[#], Nicole Kuznetsov, Elizna Maasdorp, Jonathan Carr, Riaan van Coller, Ignacio F. Mata, Soraya Bardien on behalf of the Global Parkinson's Genetics Program (GP2)

[#] These authors share first authorship

Table of contents:

Supplementary Methods 3
Sanger sequencing 3
Supplementary figures 3
Supplementary Figure 1: Example of Log R Ratio (LRR) plots showing PRKN exon 4 CNVs for (A) homozygous deletion and (B) heterozygous deletion detected with CNV-Finder. The exons are plotted in red with exon 1 on the right of the figure and exon 12 on the left 3
Supplementary Figure 2: CNV in 5' untranslated region of SNCA. The gene region is plotted in red. The UCSC Genome Browser was used to confirm the location of the CNV. BAF; B Allele Frequency; LRR, Log R Ratio 4
Supplementary tables 5
Supplementary Table 1: Proband demographics for the South African Parkinson's Disease Study Collection 5
Supplementary Table 2: Previously identified pathogenic missense variants in the South African Parkinson's Disease Study Collection 5
Supplementary Table 3: Polymerase chain reaction primer information and reaction conditions 6
Supplementary Table 4: Polymerase chain reaction reagents and volumes 8
Supplementary Table 5: Thermal cycling conditions used for polymerase chain reaction 8
Supplementary Table 6: Previously identified copy number variations in the South African Study Collection 9
Supplementary Table 7: Variants of unknown significance identified in the present study 11
References 11
Global Parkinson's Genetics Program banner author list 13

Supplementary Methods

Sanger sequencing

For Sanger sequencing, the PCR product and the relevant primers at a 1.1µM concentration (or 10µM for the *GBA1* variants) were submitted to the DNA Sequencing Unit at the Central Analytical Facilities, Stellenbosch University for Sanger sequencing. Post-PCR purification was conducted using the Nucleofast 96-well post-PCR clean-up system (Nagel, Germany). DNA sequencing was performed with the BigDye Terminator v3.1 Cycle Sequencing Kit (Applied Biosystems, USA) according to the manufacturer's instructions. This included an initial denaturation at 94 °C for 5 minutes, followed by 25 cycles of denaturation at 94 °C for 10 seconds, annealing at 55 °C for 10 seconds, and extension at 60 °C for 4 minutes. Finally, capillary electrophoresis was conducted on either an ABI 3500xI or ABI 3730xI Genetic Analyzer, equipped with a 50 cm capillary array and POP7 polymer (Applied Biosystems, USA).

Supplementary figures

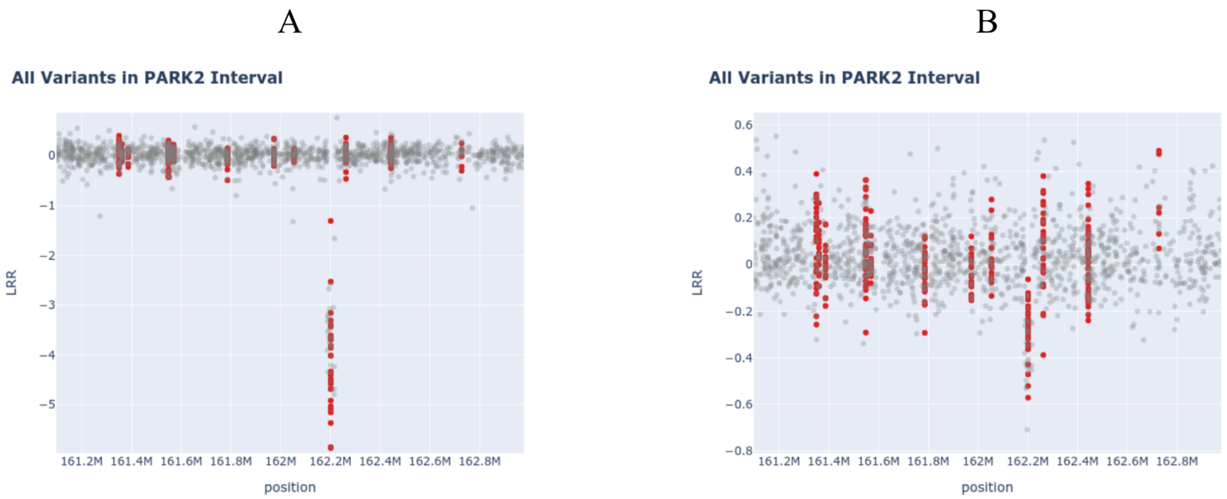

Supplementary Figure 1: *Example of Log R Ratio (LRR) plots showing PRKN exon 4 CNVs for (A) homozygous deletion and (B) heterozygous deletion detected with CNV-Finder. The exons are plotted in red with exon 1 on the right of the figure and exon 12 on the left.*

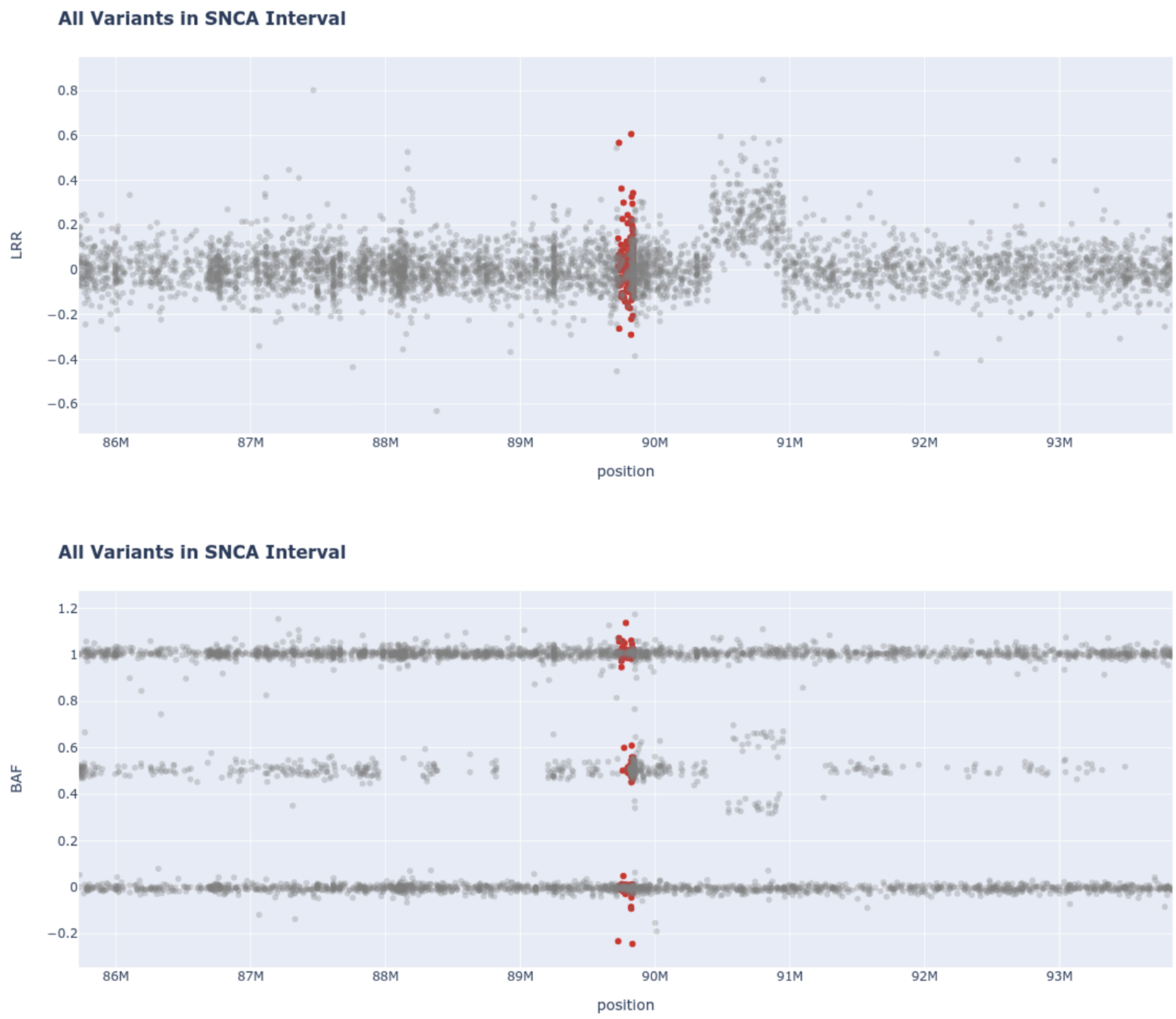

Supplementary Figure 2: *CNV in 5' untranslated region of SNCA. The gene region is plotted in red. The UCSC Genome Browser was used to confirm the location of the CNV. BAF; B Allele Frequency; LRR, Log R Ratio*

Supplementary tables

Supplementary Table 1: *Proband demographics for the South African Parkinson's Disease Study Collection*

Participant group	Number of participants	Mean age at onset (SD)	Males, n (%)	Females, n(%)
Probands	689	57.3 (12.8)	403 (58.49%)	286 (40.97%)
Legend: SD, Standard Deviation				

Supplementary Table 2: *Previously identified pathogenic missense variants in the South African Parkinson's Disease Study Collection*

GP2 ID / our lab ID/ family number	Gene symbol	Pathogenic variant, zygosity
001505/ 82.47/ 154	*LRRK2*	p.G2019S, Het
001520/ 11.640/ 456	*LRRK2*	p.G2019S, Het
001508/ 84.25/ 206	*LRRK2*	p.G2019S, Hom
001522/ 13.002/ 594	*LRRK2*	p.G2019S, Het
001521/ 12.482/ 523	*LRRK2*	p.G2019S, Het
001525/ 68.06/ 85	*LRRK2*	p.G2019S, Het
001517/ 60.47/ 40	*LRRK2*	p.G2019S, Het
001504/ 81.64/ 133	*LRRK2*	p.G2019S, Het
001512/ 85.57/ 224	*LRRK2*	p.R1441C, Het
001499/ 78.76/ 108	*PRKN*	p.G430D, Het
001498/ 78.74/ 107	*PRKN*	p.G430D, Het
001515/ 95.94/ 340	*PRKN*	p.R275W/ p.M432V, Hom
001526/ 71.95/ 88	*PINK1*	p.Y258X, Hom
Legend: Het, heterozygous; Hom, homozygous		

Supplementary Table 3: *Polymerase chain reaction primer information and reaction conditions*

**Gene**	**Exon**	**SNV**	**Primer sequence (5'– 3')**	**Tm (°C)**	**PCR condition (Ta)(°C)**	**Size of fragment (Bp)**
GBA	Large fragment	Both p.T408M and p.N409S	For: GTT-GTC-ACC-CAT-ACA-TGC-CC<br>Rev: CTC-TCA-TGC-ATT-CCA-GAG-GC	62<br>62	64	7kb
GBA	9	p.T408M	For: GCA-ACT-CTG-GGG-AAC-CA<br>Rev: CAT-TGG-GGT-TTT-CTG-TTG-CT	54<br>58	58	700
GBA	10	p.N409S	For: AAG-GGC-TTC-TGT-CAG-TCT-TTG<br>Rev: GTA-CAT-TGG-TAC-CTG-GAC-TTT-CT	62<br>66	58	260
LRRK2	4	p.L119P	For: AAC-ACA-TGA-GCT-TCA-CTA-CAG-G<br>Rev: GGT-CTT-CAG-TCC-AAT-CAC-TGA-C	64<br>66	59.7	200
LRKK2	19	p.R793M	For: GAG-AAA-GAG-AGC-AGT-CCC-AAA-T<br>Rev: TAA-AGG-ACC-AAG-CCA-AGA-AGG	64<br>62	59.7	216
LRKK2	29	p.R1325Q	For: GTG-TGA-CAT-GTA-AAA-GAA-CTC-ACC<br>Rev: CCC-TGT-TCC-AAA-CAA-ATG-GT	68<br>58	58	368
PINK1	5	p.A339T and p.A359T	For: CGT-CGA-TGT-GTG-GTA-GCC<br>Rev: TCT-AGT-GCC-CCT-GGA-GAG-C	58<br>62	57.1	250
PINK1	7	p.A494V	For: ATG-GGC-GGG-CAG-CGT-GAT-GTC-T<br>Rev: CTG-GAA-CGA-GAA-CAG-AGG-TTT-C	72<br>66	56.7	339
PLA2G6	2	p.S34L and p.D31N	For: CCT-CAG-ACA-GAG-ACT-CAA-AGA-AA<br>Rev: CGC-CTG-GTC-AAT-ACC-TTC-A	66<br>58	59	300
PLA2G6	13	p.G361S	For: CGG-AAC-ACC-TCA-TCC-TTC-AT<br>Rev: GGC-CCA-GAA-TTT-GGG-TTT-G	56<br>58	59	200
POLG	20	p.V1044A	For: CTC-TGC-CCA-TGC-TCC-AAA<br>Rev: CCT-TAG-GTC-ACA-GTG-GAA-GAA-G	56<br>66	59	206
PRKN	9	p.R334C	For: TCC-CAT-GCA-CTG-TAG-CTC-CT<br>Rev: CCA-GCC-CAT-GTG-CAA-AAG-C	62<br>60	56.7	297

PRKN	11	p.R402C	For: CCG-ACG-TAC-AGG-GAA-CAT-AAA<br>Rev: GGC-ACC-TTC-AGA-CAG-CAT-CT	62<br>62	56.7	300
PRKN	12	p.G430D	For: AAT-GAA-GGT-AGA-CAC-TGG-GTA-TG<br>Rev: GGT-TTG-GGA-ATG-CGT-GTT-T	66<br>56	56.7	246
PRKN	12	p.P437L	For: TCT-AGG-CTA-GCG-TGC-TGG-TT<br>Rev: GCG-TGT-GTG-TGT-GTG-TTT-GA	62<br>60	56.7	300
SYNJ1	17	p.I707T	For: AGC-AGC-AAG-AGC-TTA-CCA-TAG<br>Rev: CAA-AGG-GAT-GTT-GCA-GTT-GAT-AC	62<br>66	59	214

Legend: °C, degree Celsius; 5’-3’, 5 prime to 3 prime direction; Ta, Annealing temperature; Bp, Base pair; For, Forward primer; Tm, Melting temperature; Rev: Reverse primer; SNV: Single nucleotide variant.

Supplementary Table 4: *Polymerase chain reaction reagents and volumes*

**Reagent**	**Volume per reaction**
Thermo Fisher Scientific DreamTaq™ hot start PCR master mix	25µl
Nuclease-free-water	21µl
Forward primer (20µM)	1µl
Reverse primer (20µM)	1µl
Genomic DNA	2µl
Total	50µl
Legend: DNA, Deoxyribonucleic acid; µl, Microliter; PCR, Polymerase chain reaction	

Supplementary Table 5: *Thermal cycling conditions used for polymerase chain reaction*

**Temperature and time**	**Number of cycles**	**PCR step**
95ºC – 3 minutes		Initial Denaturation
95ºC – 30 seconds	30 Cycles	Denaturation
58ºC – 30 seconds		Annealing
72ºC – 30 seconds		Extension
72ºC – 10 minutes		Final Extension
4ºC		Hold
Legend: ºC, degree Celsius; PCR, polymerase chain reaction		

Supplementary Table 6: *Previously identified copy number variations in the South African Study Collection*

GP2 ID/ our lab ID/ family number	Proband	CNV type	Variant	CNV-Finder prediction value	Confirmed with MLPA	AAO	Family history	Citation
PRKN **CNVs**								
001498/ 78.74/ 107	Yes	DEL	Exon 4 Het	0.0781*	Yes	56	No	(Haylett et al. 2012)
001494/ 37.12/ 1	Yes	DEL & DUP	Exon 9 Het & Exon 2 Het	DEL: 0.6827; DUP: 0.0137*	Yes	45	Yes	(Keyser et al. 2010)
001493/ 37.13/ 1	No	DEL & DUP	Exon 9 Het & Exon 2 Het	DEL: 0.7033; DUP: 0.0311*	Yes	Unknown	Yes	(Keyser et al. 2010)
001500/ 81.03/ 121	Yes	DUP	Exon 2-6 Het & Exon 5 Het	1	Yes	48	Yes	(Haylett et al. 2012)
000024/ 55.54/ 31	Yes	DUP	Exon 2 Het	1	Yes	65	No	(Keyser et al. 2010; Haylett et al. 2012)
001496/ 77.60/ 105	Yes	DEL	Exon 3 Het	1	Yes	25	No	(Keyser et al. 2010)
001497/ 82.15/ 105	No	DEL	Exon 3 Het	1	Yes	Unaffected	Yes	(Haylett et al. 2012)
001499/ 78.76/ 108	Yes	DEL	Exon 4 Het	0.9948	Yes	27	No	(Haylett et al. 2012)
001513/ 53.44/ 23	Yes	DEL	Exon 3-4 Hom	1	Yes	27	No	(Bardien et al. 2009)
001506/ 34.22/ 16	No	DEL	Exon 4 Hom	1	Yes	22	Yes	(Bardien et al. 2009)
001507/ 56.45/ 16	Yes	DEL	Exon 4 Hom	1	Yes	22	Yes	(Bardien et al. 2009)
000081/ 68.22/ 91	Yes	DUP	Exon 2-3 Het	1	Yes	50	No	(Keyser et al. 2010; Haylett et al. 2012)
000332/ 96.69/ 358	Yes	DEL	Exon 4 Het	0.9011	Yes	48	No	(van der Merwe et al. 2016)
000109/ 81.27/ 126	Yes	DEL	Exon 4 Het	0.5226*	Yes	49	Yes	(Haylett et al. 2012)

000195/ 99.48/ 217	Yes	DEL	Exon 3-4 Het	1	Yes	61	No	(Haylett et al. 2012)
***SNCA* CNVs**								
001484/ 11.789/ 459	Yes	DUP	Gene	1	Yes	35	Yes	(Braun et al. 2025)
001485/ 11.617/ 459	No	DUP	Gene	1	Yes	43	Yes	(Braun et al. 2025)
001486/ 11.861/ 459	No	DUP	Gene	1	Yes	63	Yes	(Braun et al. 2025)
001501/ 75.49/ 13	No	TRIP	Gene	1	Yes	Unaffected	Yes	(Keyser et al. 2010)
001502/ 75.51/ 13	Yes	TRIP	Gene	1	Yes	34	Yes	(Keyser et al. 2010)

The CNV-Finder prediction value represents the confidence score for each identified CNV, with values closer to 1 indicating higher confidence in the CNV call. * indicates the prediction values too low for CNV-Finder to confidently call the known CNV. The 'STELLENBOS_' prefix included in the GP2 IDs has been removed for presentation in the table. AAO, age at onset; CNV, Copy number variations; DEL, deletion; DUP, duplication; Het, heterozygous; Hom, Homozygous; MLPA, multiplex ligation-dependent probe amplification; PRKN, Parkin; SNCA, alpha-synuclein; TRIP, triplication

Supplementary Table 7: *Variants of unknown significance identified in the present study*

**rsID**	**Pathogenic variant, zygosity**	**Gene**	**Number of probands**	**Mean AAO (SD)**
rs144538200	p.N502S, het	*FBXO7*	2	60.5 (14.85)
rs149487315	p.M400I, het	*GBA*	2	54.50 (9.19)
rs1141812	p.R83C, het	*GBA*	1	70
rs145946172	p.G26E, het	*HTRA2*	1	60
rs143710836	p.A1215T, het	*LRRK2*	1	69
rs77428810	p.R1941H, het	*LRRK2*	1	62
rs71653622	p.A179T, het	*PARK7*	1	34
rs144440543	p.L461R, het	*PINK1*	1	45
rs759468742	p.R207Q, het	*PINK1*	2	64 (21.21)
rs113092523	p.S284Y, het	*PINK1*	1	50
rs202048763	p.S73L, het	*PINK1*	1	53
rs150562946	p.R256C, het	*PRKN*	3	68.33 (2.89)
rs114696251	p.Y267H, het	*PRKN*	1	65
rs142964720	p.G1144D, het	*SYNJ1*	1	41
rs114053718	p.I866T, het	*SYNJ1*	4	59.80 (9.70)
rs866258846	p.L1406S, het	*SYNJ1*	1	62
rs760863245	p.P1199S, het	*SYNJ1*	1	68
rs546986773	p.Q1303R, het	*SYNJ1*	1	74

AAO, age at onset; Het, heterozygous; SD, standard deviation. These variants are reported as uncertain significance by ClinVar as of July 2025.

References

Bardien, Soraya, Rowena Keyser, Yandiswa Yako, Debbie Lombard, and Jonathan Carr. 2009. "Molecular Analysis of the Parkin Gene in South African Patients Diagnosed with Parkinson's Disease." *Parkinsonism & Related Disorders* 15 (2): 116–21.

Braun, Abigail, Dion Basson, Shahida Moosa, Jonathan Carr, Soraya Bardien, and Riaan van Coller. 2025. "Deep Brain Stimulation Improves Symptoms in an Individual with Alpha-Synuclein-Gene-Associated Parkinson's Disease." *Movement Disorders Clinical Practice* 12 (8): 1200–1203.

Haylett, William L., Rowena J. Keyser, Melissa C. du Plessis, Celia van der Merwe, Janine Blanckenberg,

Debbie Lombard, Jonathan Carr, and Soraya Bardien. 2012. “Mutations in the Parkin Gene Are a Minor Cause of Parkinson’s Disease in the South African Population.” *Parkinsonism & Related Disorders* 18 (1): 89–92.

Keyser, Rowena J., Debbie Lombard, Rene Veikondis, Jonathan Carr, and Soraya Bardien. 2010. “Analysis of Exon Dosage Using MLPA in South African Parkinson’s Disease Patients.” *Neurogenetics* 11 (3): 305–12.

Merwe, Celia van der, Jonathan Carr, Brigitte Glanzmann, and Soraya Bardien. 2016. “Exonic Rearrangements in the Known Parkinson’s Disease-Causing Genes Are a Rare Cause of the Disease in South African Patients.” *Neuroscience Letters* 619 (April):168–71.

Global Parkinson's Genetics Program banner author list

Country	Name	Email address	Institution	City	State/Province (USA, Canada, Australia only)	Funders and Disclosures
**Algeria**	Yasser Mecheri	mecheriyasser@outlook.com	Centre Hospitalo-Universitaire Dr Benbadis Constantine	Constantine		Nothing to declare
**Algeria**	Bouchetara Mohamed Sofiane	neurobouchetara@yahoo.fr	Hospital university of Oran-Algeria	Oran		Nothing to declare
**Algeria**	Benhassine Traki	trakibenhassine@hotmail.com	Faculty of Biological Sciences, USTHB Bab Ezzouar, Algiers	Algiers		Nothing to declare
**Argentina**	Emilia M Gatto	egatto@ineba.net	Sanatorio de la Trinidad Mitre- INEBA	Buenos Aires		Nothing to declare
**Argentina**	Marcelo Kauffman	mkauffman@austral.edu.ar	Hospital JM Ramos Mejia	Buenos Aires		Nothing to declare
**Argentina**	Federico Capparelli	fcapparelli@fleni.org.ar	Centro de Educación Médica e Investigaciones Clínicas Norberto Quirno	Buenos Aires		Nothing to declare
**Argentina**	Maria Valentina Muller	mariavalentinamuller@gmail.com	Hospital General San Martin	La Plata		Nothing to declare
**Argentina**	Marcela Susana Tela	marcetela@gmail.com	Hospital Fernandez	Buenos Aires		Nothing to declare
**Argentina**	Adamec, Dario Sergio	darioadamec@yahoo.com.ar	HOSPITAL NACIONAL PROFESOR ALEJANDRO POSADAS	Buenos Aires		Nothing to declare
**Argentina**	Cesar Luis Avila	cesar.avila@fbqf.unt.edu.ar	CONICET-UNT	San Miguel de Tucumán		Nothing to declare

**Argentina**	Valentin Selvaggi	selvaggi.valentin@gmail.com	Hospital Ramos Mejia	Buenos Aires		Nothing to declare
**Argentina**	Nahuel Fonseca	nahufonseca@gmail.com	Consejo Nacional de Investigaciones Científicas y Técnicas	Buenos Aires		Nothing to declare
**Argentina**	Elena AVALE	elena.avale@conicet.gov.ar	CONICET	Buenos Aires		Nothing to declare
**Argentina**	Marcelo Merello	mmerello@fleni.org.ar	Fleni	Buenos Aires		Nothing to declare
**Argentina**	Griselda Judith Alvarado	grialvarado.ga@gmail.com	SIPROSA	Tucumán		Nothing to declare
**Argentina**	Juan Pablo Diaz Rearte	jotapegc@gmail.com	SIPROSA	Tucumán		Nothing to declare
**Argentina**	Luciana Rojas Vazquez	lucianarojasvazquez@gmail.com	SIPROSA	Tucumán		Nothing to declare
**Argentina**	Lucía Wang	parkinson.arg@gmail.com	PARKINSON ARGENTINA/ALAPA	Buenos Aires		Nothing to declare
**Argentina**	Carlos Matias López Razquin	servicioneuro.sm@gmail.com	H.I.G.A. "Gral. San Martín" - ALAPA	La Plata		Nothing to declare
**Argentina**	Andrea Bril	Av Cerviño 3356	Hospital Fernández	Buenos Aires		Nothing to declare
**Argentina**	Florencia Nicole Wainberg	fwainberg.ext@fleni.org.ar	Fleni	Buenos Aires		Nothing to declare
**Argentina**	Maria Fernanda López	mariafernanda.lopez@hospitalposadas.gob.ar	Hospital Nacional Alejandro Posadas. Argentina	El Palomar		Nothing to declare
**Armenia**	Samson Khachatryan	drsamkhach@gmail.com	Somnus Neurology Clinic	Yerevan		Nothing to declare
**Armenia**	Zaruhi Tavadyan	zdtavadyan@gmail.com	Somnus Neurology Clinic	Yerevan		Nothing to declare

**Armenia**	Mariam Isayan	drmariamisayan@gmail.com	Somnus Neurology Clinic	Yerevan		Nothing to declare
**Australia**	Claire E Shepherd	c.shepherd@neura.edu.au	Neuroscience Research Australia	Sydney	New South Wales	The Sydney Brain Bank is located at and supported by Neuroscience Research Australia
**Australia**	Kishore Kumar	kkum4618@uni.sydney.edu.au	Garvan Institute of Medical Research and Concord Repatriation General Hospital	Darlinghurst	New South Wales	Paul Ainsworth Family Foundation
**Australia**	Melina Ellis	melina.ellis@health.nsw.gov.au	Concord Hospital	Concord	New South Wales	Nothing to declare
**Australia**	Miguel E. Rentería	miguel.renteria@qimrberghofer.edu.au	QIMR Berghofer Medical Research Institute	Herston	Queensland	The Australian Parkinson's Genetics Study is supported by the Shake It Up Australia Foundation and The Michael J. Fox Foundation for Parkinson's Research
**Australia**	Sulev Koks	sulev.koks@murdoch.edu.au	Murdoch University	Perth	Western Australia	Nothing to declare

**Australia**	Simon Rowe	s.rowe@neura.edu.au	Neuroscience Research Australia	Sydney	New South Wales	Nothing to declare
**Australia**	Dennis Yeow	d.yeow@neura.edu.au	Neuroscience Research Australia	Sydney	New South Wales	Nothing to declare
**Australia**	Carolyn Sue	c.sue@neura.edu.au	Neuroscience Research Australia	Sydney	New South Wales	Nothing to declare
**Australia**	Victor Flores Ocampo	victorfloresocampo@yahoo.com.mx	QIMR Berghofer Medical Research Institute	Brisbane	Queensland	Nothing to declare
**Australia**	Christine Wools	christine.wools@mh.org.au	Epworth hospital	Melbourne	Victoria	Nothing to declare
**Australia**	Keren Aliza Weiss	k.weiss@garvan.org.au	Garvan Institute of Medical Research	Sydney	New South Wales	Nothing to declare
**Australia**	Ryan L Davis	ryan.davis@sydney.edu.au	University of Sydney	Sydney	New South Wales	Nothing to declare
**Australia**	Amanda Willis	a.willis@garvan.org.au	Garvan Institute of Medical Research	Sydney	New South Wales	Nothing to declare
**Australia**	Steven He	s.he@garvan.org.au	Garvan Institute of Medical Research	Sydney	New South Wales	Nothing to declare
**Australia**	Robert Arthur Wilcox	Robert.Wilcox@sa.gov.au	Flinders Medical Centre	Bedford Park	South Australia	Nothing to declare
**Australia**	Denise Howting	denise.howting@perron.uwa.edu.au	Perron Institute for Neurological and Translational Science	Nedlands	Western Australia	Nothing to declare
**Australia**	Jack David Price	jack.price@perron.uwa.edu.au	Perron Institute	Perth	Western Australia	Nothing to declare
**Australia**	Pak Leng Cheong	pak.cheong@health.nsw.gov.au	Sydney Local Health District	Sydney	New South Wales	Nothing to declare

**Australia**	Michel Tchan	michel.tchan@health.nsw.gov.au	Westmead Hospital	Westmead	New South Wales	Nothing to declare
**Australia**	Mary-Anne Young	m.young@garvan.org.au	MonoPD	Sydney	New South Wales	Nothing to declare
**Australia**	Catriona Mclean	c.mclean@alfred.org.au	Florey neuroscience	Melbourne	Victoria	Nothing to declare
**Australia**	Nicholas G. Martin	nick.martin@qimrb.edu.au	QIMR Berghofer Medical Research Institute	Brisbane	Queensland	Nothing to declare
**Australia**	Hugo Morales Briceño	hugo.moralesbriceno@health.nsw.gov.au	Westmead Hospital	Sydney	New South Wales	Nothing to declare
**Australia**	Thomas Kimber	thomas.kimber@sa.gov.au	Central Adelaide Local Health Network	Adelaide	South Australia	Nothing to declare
**Australia**	Kathy H. C. Wu	kathy.wu@svha.org.au	St Vincent's Hospital Sydney	Darlinghurst	New South Wales	Nothing to declare
**Australia**	John O'Sullivan	john.osullivan@uq.edu.au	University of Queensland	Brisbane	Queensland	Nothing to declare
**Australia**	Lewis M Singleton	lewis.singleton@perron.uwa.edu.au	Perron Institute of Neurological and Translational Science	Perth	Western Australia	Nothing to declare
**Australia**	Laura Ivete Rudaks	laura.rudaks@gmail.com	Concord Repatriation General Hospital	Sydney	New South Wales	Nothing to declare
**Australia**	Luis M. García-Marín	garciamarinlm@gmail.com	Queensland Institute of Medical Research (QIMR Berghofer)	Brisbane	Queensland	Nothing to declare
**Australia**	Amanda Lim	amanda.limwy@gmail.com	Queensland Institute of Medical Research (QIMR Berghofer)	Brisbane	Queensland	Nothing to declare
**Australia**	Sue-Faye Siow	s.siow@garvan.org.au	Garvan Institute of Medical Research	Sydney	New South Wales	Nothing to declare
**Australia**	Natalia Soledad	natalia.ogonowski@qimrb.edu.au	QIMR Berghofer	Brisbane	Queensland	Nothing to declare

	Ogonowski					
**Australia**	Santiago Diaz Torres	Santiago.DiazTorres@qimrb.edu.au	Queensland Institute of Medical Research (QIMR Berghofer) / The University of Queensland	Brisbane	Queensland	Nothing to declare
**Australia**	Fangyuan Cao	fangyuan.cao@qimrb.edu.au	QIMR Berghofer	Brisbane	Queensland	Nothing to declare
**Austria**	Alexander Zimprich	alexander.zimprich@meduniwien.ac.at	Medical University Vienna Austria	Vienna		Nothing to declare
**Azerbaijan**	Kanan Jafarov	kcaferov1975@gmail.com	Istanbul Klinik	Baku		Nothing to declare
**Bangladesh**	Imran Sarker	imran37mmc@gmail.com	National Institute of Neurosciences and Hospital	Dhaka		Nothing to declare
**Belgium**	David Crosiers	david.crosiers@uantwerpen.be	University of Antwerp	Antwerp		Nothing to declare
**Bolivia**	Erick Gonzalez Delgado	erick.gonzalez@medicalcenter.bo	MedicalCenter Neurocenter S.A.	Santa Cruz de la Sierra		Nothing to declare
**Brazil**	Artur F. Schumacher-Schuh	aschuh@hcpa.edu.br	Universidade Federal do Rio Grande do Sul / Hospital de Clínicas de Porto Alegre	Porto Alegre		Nothing to declare
**Brazil**	Carlos Rieder	carlosrieder@gmail.com	Federal University of Health Sciences of Porto Alegre	Porto Alegre		Nothing to declare
**Brazil**	Paula Saffie Awad	paula.saffie@clinicasantamaria.cl	Universidade Federal do Rio Grande do Sul	Porto Alegre		Global Parkinson's Genetics Program
**Brazil**	Vitor Tumas	tumasv@fmrp.usp.br	University of São Paulo	São Paulo		Nothing to declare
**Brazil**	Sarah Camargos	sarahcamargos@ufmg.br	Universidade Federal de Minas Gerais	Belo Horizonte		Nothing to declare
**Brazil**	Lucas Faria Costa	farialucas13@gmail.com	Universidade Federal de Minas Gerais	Belo Horizonte		Nothing to declare

**Brazil**	Pedro Braga Neto	pbraganeto@ufc.br	Federal University of Ceará	Fortaleza		Nothing to declare
**Brazil**	Juan Sebastián Sánchez León	jsleon@hcpa.edu.br	Hospital de Clínicas de Porto Alegre	Porto Alegre		Nothing to declare
**Brazil**	Maira Rozenfeld Olchik	molchik@hcpa.edu.br	Hospital de Clínicas de Porto Alegre (HCPA)	Porto Alegre		Nothing to declare
**Brazil**	Bruno Lopes Santos-Lobato	bruls@ufpa.br	Hospital Ophir Loyola	Belém		Nothing to declare
**Brazil**	Grace Helena Letro	centrodepesquisa@hospitalda-puc-campinas.edu.br	Hospital da Pontificia Universiade Católica de Campinas (Hospital PUC-Campinas	Campinas		Nothing to declare
**Brazil**	Daniel Teixeira-dos-Santos	danielsantos@hcpa.edu.br	Hospital de Clinicas de Porto Alegre	Porto Alegre		Nothing to declare
**Brazil**	Henrique Ballalai Ferraz	hbferraz@unifesp.br	Universidade Federal de São Paulo (UNIFESP/EPM)	São Paulo		Nothing to declare
**Brazil**	Carolina Candeias da Silva	ccsilva@unifesp.br	Universidade Federal de São Paulo UNIFESP/EPM	São Paulo		Nothing to declare
**Brazil**	Vanderci Borges	v.borges@unifesp.br	Universidade Federal de São Paulo UNIFESP/EPM	São Paulo		Michael G. Fox Foundation and Fundação de Apoio à Pesquisa -FAP UNIFESP
**Brazil**	Dayany Leonel Boone	dayany.boone@unifesp.br	Universidade Federal de São Paulo - UNIFESP/EPM	São Paulo		Nothing to declare

**Brazil**	Mariana Cavalcanti Costa	mariana.cavalcanti@unifesp.br	Universidade Federal de São Paulo - UNIFESP/EPM	São Paulo		Nothing to declare
**Canada**	Oury Monchi	oury.monchi@umontreal.ca	Institut universitaire de gériatrie de Montréal	Montreal	Quebec	CIHR, Brain Canada, Parkinson Canada
**Canada**	Edward Fon	ted.fon@mcgill.ca	McGill University	Montreal	Quebec	Nothing to declare
**Canada**	Robert Thibault	rthibault@parkinsonsroadmap.org	Aligning Science Across Parkinson's	Vancouver	British Columbia	Nothing to declare
**Canada**	Ziv Gan-Or	ziv.gan-or@mcgill.ca	McGill University	Montreal	Quebec	Nothing to declare
**Canada**	Meron Teferra	meron.teferra@mcgill.ca	McGill University	Montreal	Quebec	Nothing to declare
**Canada**	Anthony Lang	Anthony.Lang@uhnresearch.ca	University of Toronto	Toronto	Ontario	Nothing to declare
**Canada**	Konstantin Senkevich	konstantin.senkevich@mcgill.ca	McGill University	Montreal	Quebec	Nothing to declare
**Chile**	Marcelo Miranda	Directiva@fundaciondiagnosis.cl	Departament of Neurology, Clinica MEDS, Santiago , Chile . Fundación Diagnosis , Santiago , Chile	Santiago		Nothing to declare
**Chile**	María Leonor Bustamante	mbustamante@uchile.cl	Faculty of Medicine Universidad de Chile	Santiago		Nothing to declare
**Chile**	Juan Cristobal Nuñez	jc.nunezf@gmail.com	Universidad de Chile - Clínica Alemana Santiago	Santiago		Nothing to declare
**Chile**	Boris Lucero	bluceroucm@gmail.com	Universidad Católica del Maule	Talca		Nothing to declare
**Chile**	Alicia Colombo	acolombo@hcuch.cl	University of Chile	Santiago		Nothing to declare

**Chile**	Maria Teresa Muñoz Personal	mtmunoz@uchile.cl	Universidad de Chile	Santiago		Internal Research Fund – Occupational pesticide exposure and Parkinson's disease risk in older Chilean adults from the LARGE-PD cohort, School of Public Health, Universidad de Chile.<br><br>FONDECYT Regular 1240899 – Environmental exposure to pesticides and neurobehavioral and neurophysiological effects in adults from rural communities in the Maule Region, Chile
**Chile**	Eduardo Pérez Palma	eperezpalma@gmail.com	Universidad del Desarrollo	Santiago		Nothing to declare

**Chile**	Pedro Chana-Cuevas	pchana@cetram.org	Universidad de Santiago de Chile	Santiago		Nothing to declare
**Chile**	Ana Belen Miranda Cortes	anamirandacortes@gmail.com	Fundación Diagnosis	Santiago		Nothing to declare
**Chile**	María Eugenia Contreras Pinto	mecontreraspinto@gmail.com	Hospital San Juan de Dios	La serena		Nothing to declare
**Chile**	Francisca Canals	mfcanals@gmail.com	Inmov	Santiago		Nothing to declare
**Chile**	Patricio Alejandro Olguín Aguilera	patricioolguin@uchile.cl	Universidad de Chile, Facultad de Medicina	Santiago		Nothing to declare
**Chile**	Elias Fernandez-Toledo	elias.mft@gmail.com	University of Concepción	Concepcion		Nothing to declare
**Chile**	Benjamin Pizarro Galleguillos	benjaminpizarro@ug.uchile.cl	Centro de Imagenología, Hospital Clínico Universidad de Chile	Santiago		Nothing to declare
**Chile**	Carlos Hernandez	carlhernandeza@udd.cl	Universidad del Desarrollo	Santiago		Nothing to declare
**Chile**	Floria C. Pancetti	pancetti@ucn.cl	Universidad Católica del Norte	Coquimbo		Nothing to declare
**Chile**	Juan Cristobal Nuñez Fuster	jc.nunezf@gmail.com	Hospital Clínico Unversidad de Chile - Clínica Alemana Santiago	Santiago		Nothing to declare
**Chile**	José Manuel Fernández	cotefernandez@gmail.com	INMOV	Buenos Aires		Nothing to declare

**Chile**	Lorena Hudson Aros	lorehud@gmail.com	INMOV (Instituto Nacional de Movimientos Anormales)	Buenos Aires		Nothing to declare
**China**	Beisha Tang	bstang7398@csu.edu.cn	Central South University	Changsha		Nothing to declare
**China**	Huifang Shang	shanghuifang@scu.edu.cn	West China Hospital Sichuan University	Chengdu		Nothing to declare
**China**	Jifeng Guo	guojifeng2003@163.com	Xiangya Hospital	Changsha		Nothing to declare
**China**	Piu Chan	pbchan@hotmail.com	Capital Medical University	Beijing		Nothing to declare
**China**	Wei Luo	weiluo@zju.edu.cn	Zhejiang University	Hangzhou		Nothing to declare
**China**	Zhenhua Liu	liuzhenhua@csu.edu.cn	Xiangya Hospital, Central South University	Changsha		Nothing to declare
**China**	Germaine Hiu-Fai Chan	Chf862@ha.org.hk	Queen Elizabeth Hospital	Kowloon		Nothing to declare
**China**	Nancy Ip	boip@ust.hk	The Hong Kong University of Science and Technology	Kowloon		Nothing to declare
**China**	Nelson Yuk-Fai Cheung		Queen Elizabeth Hospital	Kowloon		Nothing to declare
**China**	Phillip Chan	philipch.chan@polyu.edu.hk	The Hong Kong University of Science and Technology	Kowloon		Nothing to declare
**China**	Xiaopu Zhou	xpzhou@ust.hk	The Hong Kong University of Science and Technology	Kowloon		Nothing to declare
**Colombia**	Gonzalo Arboleda	gharboledab@unal.edu.co	Universidad Nacional de Colombia	Bogotá		Nothing to declare
**Colombia**	Jorge Orozco	jorge.orozco@fvl.org.co	Fundación Valle del Lili	Santiago De Cali		Nothing to declare
**Colombia**	David Antonio Pineda-Salazar	dapinedasas@gmail.com	GRUPO DE NEUROCIENCIAS DE ANTIOQUIA (GNA)	Medellín		Nothing to declare

**Colombia**	Beatriz Munoz Ospina	beatriz.munoz@fvl.org.co	Fundacion Valle del Lili	Santiago De Cali		Nothing to declare
**Colombia**	Tatiana Lopez-Gonzalez	ntlopezg@gmail.com	Universidad Nacional de Colombia	Bogotá		Nothing to declare
**Colombia**	Carlos Velez-Pardo	calberto.velez@udea.edu.co	Universidad de Antioquia	Medellín		Nothing to declare
**Colombia**	Marlene Jimenez-Del-Rio	marlene.jimenez@udea.edu.co	Universidad de Antioquia	Medellín		Nothing to declare
**Colombia**	Sonia Moreno Masmela	sonia.moreno@gna.org.co	Universidad de Antioquia	Medellín		Nothing to declare
**Colombia**	Valentina Quintana Pena	valentina.quintana@northwestern.edu	Icesi University	Cali		Nothing to declare
**Colombia**	David Fernando Aguillon Niño	david.aguillon@gna.org.co	University of Antioquia	Medellín		Nothing to declare
**Colombia**	Eliana Pineda Mateus	ruth.pineda@urosario.edu.co	Universidad del Rosario	Bogotá		Nothing to declare
**Colombia**	Karen Lizzette Velasquez Mendez	klvelasquezm@unal.edu.co	Universidad Nacional de Colombia	Bogotá		Nothing to declare
**Costa Rica**	Alvaro Hernandez	ahernandez@williamblair.com	University of Costa Rica	San Jose		Nothing to declare
**Costa Rica**	Jaime Fornaguera Trías	jfornagu@gmail.com	Universidad de Costa Rica	San José		Nothing to declare

**Costa Rica**	Roger Rodriguez Monge	Roger.rodriguezmonge@ucr.ac.cr	Universidad de Costa Rica	San José		Nothing to declare
**Denmark**	Per Borghammer	borghammer@clin.au.dk	Aarhus University	Aarhus		Nothing to declare
**Dominican Republic**	Rossy Cruz Vicioso	rossy.vicioso@clinicaunionmedica.com	Unión Médica Del Norte, Clínica Universitaria	Santiago de los Caballeros		Nothing to declare
**Dominican Republic**	Alpher Perez Arias	alpher.perez@clinicaunionmedica.com	Clinica Union Medica del Norte	Santiago de los Caballeros		Nothing to declare
**Dominican Republic**	Ernestina Castro Salazar	ernestina.castro@clinicaunionmedica.com	Unión Medica del Norte	Santiago de los Caballeros		Nothing to declare
**Ecuador**	Carlos Rodriguez-Alarcon	crodriguez@interlabsa.com	Interlab	Guayaquil		Nothing to declare
**Egypt**	Mohamed Salama	mohamed-salama@aucegypt.edu	The American University in Cairo	Cairo		The AUC/ ASRT/ DAAD
**Egypt**	Ali Shalash	ali_neuro@yahoo.com	Ain Shams University Hospital	Cairo		TRAPCAF
**El Salvador**	Tatiana Ascencio	tatianaascencio@gmail.com	Dr. Andres Bello university	San Salvador		Nothing to declare
**El Salvador**	Oscar Peña-Rodas	oscar.penarodas@gmail.com	Universidad Dr Andrés Bello	San Salvador		Nothing to declare
**El Salvador**	Susana Lissette Peña Martínez	dra.susanapneuro@gmail.com	UNAB	San Salvador		Nothing to declare

**Ethiopia**	Yared Z. Zewde	yared.zenebe@aau.edu.et	Addis Ababa University	Addis Ababa		Nothing to declare
**France**	Alexis Brice	alexis.brice@icm-institute.org	Paris Brain Institute	Paris		Nothing to declare
**France**	Jean-Christophe Corvol	jean-christophe.corvol@aphp.fr	Sorbonne Université	Paris		Nothing to declare
**France**	Mari Vidailhet	marie.vidailhet@aphp.fr	Salpêtriere Hospital (AP-HP), Sorbonne Université	Paris		Nothing to declare
**France**	Mathieu Anheim	mathieu.anheim@chru-strasbourg.fr	University Hospital of Strasbourg, Strasbourg, France	STRASBOURG		Nothing to declare
**France**	Yves Agid	yves.agid@icm-institute.org	Paris Brain Institute	Paris		Nothing to declare
**France**	Louise-Laure Mariani	louise-laure.mariani@icm-institute.org	Paris Brain Institute - Sorbonne University	Paris		Nothing to declare
**France**	Alexandra Durr	alexandra.durr@icm-institute.org	Paris Brain Institute	Paris		Nothing to declare
**France**	Rascol	olivier.rascol@univ-tlse3.fr	université Toulouse	toulouse		Nothing to declare
**France**	Ory Magne Fabienne	ory.f@chu-toulouse.fr	chu toulouse	toulouse		Nothing to declare
**France**	Suzanne Lesage	laly.lesage@gmail.com	Paris Brain Institute (ICM)	Paris		Nothing to declare
**France**	Defebvre Luc	luc.defebvre@chu-lille.fr	CHU Lille	Lille		Nothing to declare
**France**	Tesson Christelle	christelle.tesson@icm-institute.org	Institut du Cerveau-Paris Brain Institute-ICM	Paris		Nothing to declare
**France**	Philippe Damier	philippe.damier@univ-nantes.fr	Nantes Université	Nantes		Nothing to declare
**France**	François Tison	francois.tison@chu-bordeaux.fr	University of Bordeaux, France	Bordeaux		Nothing to declare
**France**	Stéphane Thobois	stephane.thobois@chu-lyon.fr	Hospices civils de Lyon, Hopital Neurologique Pierre Wertheimer	BRON		Nothing to declare

**France**	Jean-Luc Houeto	jean-luc.houeto@unilim.fr	Limoges University Hospital	Limoges		Nothing to declare
**France**	Brefel Courbon Christine	christine.brefel-courbon@univ-tlse3.fr	CHU Toulouse	TOULOUSE		Nothing to declare
**France**	Sara Sambin	sara.sambin@icm-institute.org	CIC Neurosciences ,Paris Brain institute	Paris		Nothing to declare
**France**	Aymeric Lanore	lanore.aymeric@gmail.com	Paris Brain Institute	Paris		Nothing to declare
**France**	NS-PARK Consortium	/	/	/		/
**France**	Isabelle Arnulf	isabelle.arnulf@aphp.fr	Hopital Universitaire Pitie Salpetriere, AP-HP	Paris		Nothing to declare
**France**	Stéphane Lehericy	stephane.lehericy@icm-institute.org	Hopital Universitaire Pitie Salpetriere, AP-HP	Paris		Nothing to declare
**France**	Graziella Mangone	graziella.mangone@icm-institute.org	Hopital Universitaire Pitie Salpetriere, AP-HP	Paris		Nothing to declare
**France**	Poornima Menon	poornimajmenon@gmail.com	Hopital Universitaire Pitie Salpetriere, AP-HP	Paris		Nothing to declare
**France**	David Grabli	david.grabli@aphp.fr	Hopital Universitaire Pitie Salpetriere, AP-HP	Paris		Nothing to declare
**France**	Florence Cormier-Dequaire	florence.cormier@aphp.fr	Hopital Universitaire Pitie Salpetriere, AP-HP	Paris		Nothing to declare
**France**	Bertrand Degos	bertrand.degos@aphp.fr	Avicenne Hospital	Bobigny		Nothing to declare
**France**	Marie-Alexandrine	ma.glachant@icm-institute.org	Hopital Universitaire Pitie Salpetriere, AP-HP	Paris		Nothing to declare
**France**	Eve Benchetrit	eve.benchetrit@ap-hm.fr	La Timone Hospital	Marseille		Nothing to declare
**France**	Anais Raud	raud.anais@gmail.com	Centre de réadaptation de l'Estuaire & Résidence les jardines de la chenaie	Le Havre		Nothing to declare
**France**	Virginie Czernecki	virginie.czernecki@aphp.fr	Hopital Universitaire Pitie Salpetriere, AP-HP	Paris		Nothing to declare

**France**	Pierre Pouget	pierre.pouget@icm-institute.org	ICM	Paris		Nothing to declare
**France**	Tymothée Poitou	tymothee.poitou@icm-institute.org	ICM	Paris		Nothing to declare
**France**	Elodie Hainque	elodie.hainque@aphp.fr	Hopital Universitaire Pitie Salpetriere, AP-HP	Paris		Nothing to declare
**France**	Smaranda Leu Semenescu	smaranda.leu@aphp.fr	Hopital Universitaire Pitie Salpetriere, AP-HP	Paris		Nothing to declare
**France**	Pauline Dodet	pauline.dodet@aphp.fr	Hopital Universitaire Pitie Salpetriere, AP-HP	Paris		Nothing to declare
**France**	Samir Bekadar	sbekadar@gmail.com	Eli Lilly and Company	Paris		Nothing to declare
**France**	Fanny Mochel	fanny.mochel@icm-institute.org	Hopital Universitaire Pitie Salpetriere, AP-HP	Paris		Nothing to declare
**France**	Farid Ichou	f.ichou@ihuican.org	Hopital Universitaire Pitie Salpetriere, AP-HP	Paris		Nothing to declare
**France**	Rahul Gaurav	rahul.gaurav@icm-institute.org	Hopital Universitaire Pitie Salpetriere, AP-HP	Paris		Nothing to declare
**France**	Nadya Pyatigorskaya	nadya.pyatigorskaya@gmail.com	Hopital Universitaire Pitie Salpetriere, AP-HP	Paris		Nothing to declare
**France**	Romain Valabregue	romain.valabregue@upmc.fr	ICM	Paris		Nothing to declare
**France**	Cécile Galléa	cecile.gallea@icm-institute.org	ICM	Paris		Nothing to declare
**France**	Marie-Odile Habert	mohabert@gmail.com	Hopital Universitaire Pitie Salpetriere, AP-HP	Paris		Nothing to declare
**France**	Dijana Petrovska	dijana.petrovska@telecom-sudparis.eu	Telecom Sud Paris, Evry	Évry		Nothing to declare
**France**	Laetitia Jeancolas	laetitia.jeancolas@icm-institute.org	Telecom Sud Paris, Evry / ICM	Évry		Nothing to declare
**France**	Manon Gomes	manon.gomes@icm-institute.org	Hopital Universitaire Pitie Salpetriere, AP-HP	Paris		Nothing to declare

**France**	Stephanie Carvalho	stephanie.carvalho@icm-institute.org	Hopital Universitaire Pitie Salpetriere, AP-HP	Paris		Nothing to declare
**France**	Alizé Chalançon	alizé.chalancon@icm-institute.org	Hopital Universitaire Pitie Salpetriere, AP-HP	Paris		Nothing to declare
**France**	Carole Dongmo-Kenfack	cdongmok@yahoo.fr	Hopital Universitaire Pitie Salpetriere, AP-HP	Paris		Nothing to declare
**France**	Mickael Le	mickael.le@icm-institute.org	Hopital Universitaire Pitie Salpetriere, AP-HP	Paris		Nothing to declare
**France**	Amelie Bernardo	amelie.bernardo@icm-institute.org	Hopital Universitaire Pitie Salpetriere, AP-HP	Paris		Nothing to declare
**France**	Avigaelle Abitbol	avigaelle.abitbol@icm-institute.org	Hopital Universitaire Pitie Salpetriere, AP-HP	Paris		Nothing to declare
**France**	Marion Houot	marion.houot@aphp.fr	Hopital Universitaire Pitie Salpetriere, AP-HP	Paris		Nothing to declare
**France**	Sylvie Forlani	sylvie.forlani@icm-institute.org	Hopital Universitaire Pitie Salpetriere, AP-HP	Paris		Nothing to declare
**France**	Sophia Loiodice	sophia.loiodice@icm-institute.org	Hopital Universitaire Pitie Salpetriere, AP-HP	Paris		Nothing to declare
**France**	Ludmila Jornea	ludmila.jornea@icm-institute.org	Hopital Universitaire Pitie Salpetriere, AP-HP	Paris		Nothing to declare
**Georgia**	Mariam Kekenadze	mariam.kekenadze@gmail.com	Tbilisi State Medical University	Tbilisi		Nothing to declare
**Georgia**	Irine Khatiashvili	irenekhat@yahoo.com	S. Khechinashvili University Hospital	Tbilisi		Nothing to declare
**Georgia**	Maia Beridze	postdoc2002@gmail.com	Tbilisi State Medical University	Tbilisi		Nothing to declare
**Georgia**	Sophia Sopromadze	ssopromadze@yahoo.com	Ivane Javakhishvili Tbilisi State University	Tbilisi		Nothing to declare
**Georgia**	Irine Khatiashvilli	irenekhat@yahoo.com	Ivane Javakhishvili Tbilisi State University	Tbilisi		Nothing to declare

**Georgia**	Mariam Mshvenieradze	mariammshvenieridze5@gmail.com	Ivane Javakhishvili Tbilisi State University	Tbilisi		Nothing to declare
**Georgia**	Marika Megrelishvili	marikamegre@gmail.com	Ilia State University	Tbilisi		Nothing to declare
**Georgia**	Alexander Tsiskaridze	alexander.tsiskaridze@tsu.ge	Ivane Javakhishvili Tbilisi State University	Tbilisi		Nothing to declare
**Germany**	Ana Westenberger	ana.westenberger@uni-luebeck.de	University of Lübeck	Lübeck		Nothing to declare
**Germany**	Anastasia Illarionova	anastasia.illarionova@dzne.de	Deutsches Zentrum für Neurodegenerative Erkrankungen	Göttingen		Nothing to declare
**Germany**	Brit Mollenhauer	brit.mollenhauer@med.uni-goettingen.de	University Medical Center Göttingen	Göttingen		Nothing to declare
**Germany**	Christine Klein	christine.klein@neuro.uni-luebeck.de	University of Lübeck	Lübeck		CK serves as a medical Advisor to Centogene on genetic testing reports in the field of movement disorders, except Parkinson's disease, and is a member of the Scientific Advisory Board of Retromer Therapeutics

**Germany**	Eva-Juliane Vollstedt	jule.vollstedt@neuro.uni-luebeck.de	University of Lübeck	Lübeck		Nothing to declare
**Germany**	Franziska Hopfner	franziska.hopfner@med.uni-muenchen.de	Department of Neurology, University Hospital, LMU Munich	Munich		Nothing to declare
**Germany**	Günter Höglinger	Guenter.Hoeglinger@med.uni-muenchen.de	Department of Neurology, University Hospital, LMU Munich	Munich		Nothing to declare
**Germany**	Harutyun Madoev	harutyun.madoev@neuro.uni-luebeck.de	University of Lübeck	Lübeck		Nothing to declare
**Germany**	Joanne Trinh	joanne.trinh@neuro.uni-luebeck.de	University of Lübeck	Lübeck		Nothing to declare
**Germany**	Katja Lohmann	katja.lohmann@neuro.uni-luebeck.de	University of Lübeck	Lübeck		Nothing to declare
**Germany**	Manu Sharma	manu.sharma@uni-tuebingen.de	University of Tubingen	Tübingen		Dr. Sharma is further funded by the Michael J Fox Foundation, USA Genetic Diversity in PD Program: GAP-India Grant ID: 009411.
**Germany**	Sergiu Groppa	segroppa@uni-mainz.de	University of Mainz	Mainz		Nothing to declare
**Germany**	Thomas Gasser	thomas.gasser@uni-tuebingen.de	University of Tübingen	Tübingen		Nothing to declare
**Germany**	Zih-Hua Fang	Zih-Hua.Fang@dzne.de	The German Center for Neurodegenerative Diseases	Göttingen		Nothing to declare
**Germany**	Karl Heilbron	kheilbro@broadinstitute.org	Charité - Universitätsmedizin Berlin	Berlin		Former employee of 23andMe, Inc. Owns stock and/or stock options in 23andMe, Inc.

**Germany**	Wenhua Sun	wenhua.sun@uni-tuebingen.de	University of Teubingen	Tübingen		Nothing to declare
**Germany**	Inke König	inke.koenig@uni-luebeck.de	University of Lübeck	Lübeck		Nothing to declare
**Germany**	Daniela Berg	daniela.berg@uksh.de	University Medical Center Schleswig-Holstein	Lübeck		Nothing to declare
**Germany**	Bernhard Haslinger	bernhard.haslinger@tum.de	Technical University of Munich	Munich		Nothing to declare
**Germany**	Teresa Kleinz	teresa.kleinz@uni-luebeck.de	University of Lübeck	Lübeck		Nothing to declare
**Germany**	Norbert Brüggemann	norbert.brueggemann@uni-luebeck.de	University of Lübeck	Lübeck		Dr. Brüggemann received honaria from Abbvie, Esteve, Ipsen, Merz, Teva and Zambon
**Germany**	Konstantin Kufer	Konstantin.Kufer@dzne.de	German Centre for Neurodegenerative Diseases (DZNE) / University Hospital Bonn	Bonn		Nothing to declare
**Germany**	Antonia Maria Buchal	Antonia.Buchal@ukbonn.de	University Hospital Bonn	Bonn		Nothing to declare
**Germany**	Matthias Höllerhage	hoellerhage.matthias@mh-hannover.de	Hannover Medical School	Hannover		Nothing to declare
**Germany**	Florian Wegner	wegner.florian@mh-hannover.de	Hannover Medical School	Hannover		Nothing to declare
**Germany**	Nils Schroeter	Nils.Schroeter@uks.eu	UKS, University of Saarland	Homburg and Mainz		Nothing to declare

**Germany**	Kathrin Brockmann	kathrin.brockmann@uni-tuebingen.de	University of Tübingen	Tübingen		KB has received research funding from the Michael J. Fox Foundation for Parkinson's Research, the German Society for Parkinson DPG, the Health Forum Baden Wuerttemberg, the Else Kröner Fresenius Stiftung, the University of Tuebingen, and from the German Research Foundation DFG. KB is a consultant for F. Hoffmann-La Roche Ltd., Vanqua Bio, and the Michael J. Fox

						Foundation for Parkinson's Research and has received speaker honoraria from Abbvie, Lundbeck, UCB and Zambon.
**Germany**	Isabel Wurster	isabel.wurster@med.uni-tuebingen.de	University of Tübingen	Tübingen		Nothing to declare
**Germany**	Theresa Lüth	t.lueth@uni-luebeck.de	University of Lübeck	Lübeck		Nothing to declare
**Germany**	Christian Beetz	christian.beetz@centogene.com	CENTOGENE	Rostock		Nothing to declare
**Germany**	Krishnakumar Kandaswamy	krishna.kandaswamy@centogene.com	Centogene GmBH	Berlin		Nothing to declare
**Germany**	Eva Schäffer	eva.schaeffer@uksh.de	Kiel University	Kiel		Nothing to declare
**Germany**	Kirsten Zeuner	kirsten.zeuner@uksh.de	Kiel University	Kiel		Nothing to declare
**Germany**	Gregor Kuhlenbäumer	gregor.kuhlenbaeumer@uksh.de	Kiel University	Kiel		Nothing to declare
**Germany**	Peter Bauer	peter.bauer@centogene.com	Centogene GmbH	Rostock		Nothing to declare
**Germany**	Martin Klietz	klietz.martin@mh-hannover.de	Hannover Medical School	Hannover		Nothing to declare
**Germany**	Carolin Gabbert	c.gabbert@uni-luebeck.de	University of Lübeck	Lübeck		Nothing to declare
**Germany**	Alexander Balck	alexander.balck@uni-luebeck.de	University of Lübeck	Lübeck		Nothing to declare

**Germany**	Christoph Westenberger	christoph.westenberger@uni-luebeck.de	University of Lübeck	Lübeck		Nothing to declare
**Germany**	Claudia Schulte	claudia.schulte@uni-tuebingen.de	University of Tübingen	Tübingen		Nothing to declare
**Germany**	Sebastian Schade	sebastian.schade@pkd.de	Paracelsus-Elena-Klinik Kassel	Kassel		Nothing to declare
**Germany**	Michael Sommerauer	michael.sommerauer@ukbonn.de	University Hospital Bonn	Bonn		Nothing to declare
**Germany**	Andre Fienemann	andre.fienemann@student.uni-luebeck.de	University of Lübeck	Lübeck		Nothing to declare
**Germany**	Ann-Kathrin Hauser	ann-kathrin.hauser@medizin.uni-tuebingen.de	University of Tübingen	Tübingen		Nothing to declare
**Germany**	Claudia Schulte	claudia.schulte@uni-tuebingen.de	University of Tübingen	Tübingen		Nothing to declare
**Germany**	Sana Hrir	sanahrir9@gmail.com	University of Lübeck	Lübeck		Nothing to declare
**Ghana**	Albert Akpalu	alakpalu@ug.edu.gh	University of Ghana Medical School	Accra		Nothing to declare
**Ghana**	Momodou Cham	modoufa@gmail.com	Richard Novati Catholic Hospital, Catholic Health Service Trust	Accra		Nothing to declare
**Ghana**	Vida Obese	nadwos@hotmail.com	Kwame Nkrumah University of Science and Technology	Kumasi		Nothing to declare
**Ghana**	Andrew Jacobs Bilson	videyram@gmail.com	Kwame Nkrumah University of Science and Technology	Kumasi		Nothing to declare
**Greece**	Georgia Xiromerisiou	geoksirom@med.uth.gr	University of Thessaly	Volos		Nothing to declare
**Greece**	Georgios Hadjigorgiou	G.Hadjigeorgiou@euc.ac.cy	University of Thessaly	Volos		Nothing to declare
**Greece**	Ioannis Dagklis	iadaglis@phys.uoa.gr	Aristotle University of Thessaloniki	Thessaloniki		Nothing to declare

**Greece**	Ioannis Tarnanas	ioannis.tarnanas@gbhi.org	Ionian University	Corfu		Nothing to declare
**Greece**	Leonidas Stefanis	lstefanis@bioacademy.gr	Biomedical research Foundation of the Academy of Athens	Athens		PPMI2 (funded by MJFF), ALAMEDA (H2020 grant), funding by HFRI
**Greece**	Maria Stamelou	mstamelou@hygeia.gr	Diagnostic and Therapeutic Centre HYGEIA Hospital	Marousi		The non-profit organisation for scientific research in Parkinson's disease and related disorders
**Greece**	Efthymios Dadiotis	edar@med.uth.gr	University of Thessaly	Volos		Nothing to declare
**Greece**	Tsamis Konstantinos	ktsamis@uoi.gr	University of Ioannina	Ioannina		Honoraria and research funding: Sanofi, Genesis, Abbvie, Lavipharm, Medochemie, IPSEN, Bennett, PD Neurotechnology
**Greece**	Konitsiotis Spyridon	skonitso@uoi.gr	University of Ioannina	Ioannina		Honoraria and research funding: Novartis,

						Sanofi, BMS, TEVA, Merck, Genesis, Innovis, Viatris, Abbvie, Lundbeck, Angelini, UCB, Lavipharm, Φαρμασερβ, Elpen, Roche, Sandoz, Medtronic, Medison, AstraZeneca, ITF, IPSEN, PD Neurotechnology
**Greece**	Iro Boura	boura.iro@gmail.com	University of Crete	Heraklion		University of Crete, Changeing-Excellence Hub, AbbVie
**Greece**	Makrygianni Mariza	marizmak711@gmail.com	HYGEIA Hospital	Athens		Nothing to declare
**Greece**	Lina Florentin	lflorentin@alab.gr	HYGEIA Hospital	Athens		Nothing to declare
**Greece**	Maria Makrygianni	marizmak711@gmail.com	HYGEIA Hospital	Athens		Nothing to declare
**Greece**	Foivos S. Kanellos	f.kanellos@uoi.gr	University of Ioannina	Ioannina		Nothing to declare

**Greece**	Cleanthe Spanaki	kliospanaki@gmail.com	University of Crete	Heraklion		C.S. has received honoraria for lecturing, advisory fees, educational grants, and travel grants from ITF Hellas, Innovis, Merck Serono, AbbVie, CSL Behring, Roche and Teva. She received research funding from the Greek National Precision Medicine Network, the European Union's Horizon 2020 research innovation program under the Marie Sklodowska-Curie grant agreement No 101007926

						and from the European Union's program Excellence Hubs - HORIZON-WIDERA-2022-ACCESS-04-01 under grant agreement No. 101087071.
**Honduras**	Alex Medina	alexmedina84@hotmail.com	Hospital San Felipe	Tegucigalpa		Nothing to declare
**Honduras**	Evelin Álvarez Herrera	evelynah.ea@gmail.com	Universidad Tecnológica Centroamericana (UNITEC)	Tegucigalpa		Nothing to declare
**Honduras**	Heike Hesse Joya	hhesse.neurologia@gmail.com	Universidad Tecnológica Centroamericana UNITEC	Tegucigalpa		Nothing to declare
**Honduras**	Reyna M. Durón	reynamduron@gmail.com	Universidad Tecnológica Centroamericana	Tegucigalpa		Nothing to declare
**Honduras**	Glenda Oliva Fuentes	glenda.oliva13@gmail.com	Fundación Lucas para la Salud	Tegucigalpa		Nothing to declare
**Honduras**	Eduardo Jose Ponce Murillo	eduardomurillo1795@gmail.com	Universidad Tecnológica Centroamericana	Tegucigalpa		Nothing to declare
**Iceland**	Kari Stefansson	kstefans@decode.is	deCODE genetics/Amgen Inc., Reykjavik, Iceland Faculty of Medicine, University of Iceland, Reykjavik, Iceland	Reykjavik		employee of deCODE genetics/Amgen inc
**Iceland**	Hreinn Stefansson	hreinn@decode.is	deCODE genetics/Amgen Inc., Reykjavik, Iceland Faculty of Medicine, University of Iceland, Reykjavik, Iceland	Reykjavik		employee of deCODE genetics/Amgen inc

**Iceland**	Vala Palmadottir	valakp@landspitali.is	deCODE genetics/Amgen Inc., Reykjavik, Iceland Faculty of Medicine, University of Iceland, Reykjavik, Iceland	Reykjavik		employee of deCODE genetics/Amgen inc
**Iceland**	Astros Th. Skuladottir	astross@decode.is	deCODE genetics/Amgen Inc., Reykjavik, Iceland Faculty of Medicine, University of Iceland, Reykjavik, Iceland	Reykjavik		employee of deCODE genetics/Amgen inc
**India**	Asha Kishore	Asha.kishore@asterdmhealthcare.com	Aster Medcity	Kochi		Michael J. Fox Foundation
**India**	Divya Kp	drdivyakp@sctimst.ac.in	Sree Chitra Tirunal Institute for Medical Sciences and Technology	Thiruvananthapuram		Nothing to declare
**India**	Pramod Pal	palpramod@nimhans.ac.in	National Institute of Mental Health & Neurosciences	Bengaluru		Nothing to declare
**India**	Prashanth Lingappa Kukkle	drprashanth.lk@movementdisordersclinic.com	Manipal Hospital	Delhi		Nothing to declare
**India**	Roopa Rajan	rooparajan@aiims.edu	All India Institute of Medical Sciences	Delhi		Nothing to declare
**India**	Rupam Borgohain	b_rupam@hotmail.com	Nizam's Institute Of Medical Sciences	Hyderabad		Nothing to declare
**Iran**	Mehri Salari	mehri.salari@gmail.com	Shahid Beheshti University of Medical Science	Tehran		Nothing to declare
**Israel**	Tamara Shiner	tamarsh@tlvmc.gov.il	Tel Aviv Sourasky Medical Center	Tel Aviv-Yafo		Nothing to declare
**Israel**	Avner Thaler	avnert@tlvmc.gov.il	Tel Aviv Sourasky Medical Center	Tel Aviv-Yafo		Nothing to declare
**Israel**	Noa Bregman	noabr@tlvmc.gov.il	Tel Aviv Medical Center	Tel Aviv-Yafo		Nothing to declare
**Italy**	Andrea Quattrone	an.quattrone@icloud.com	Magna Græcia University of Catanzaro	Catanzaro		Nothing to declare
**Italy**	Enza Maria Valente	enzamaria.valente@unipv.it	University of Pavia	Pavia		Nothing to declare

**Italy**	Grazia Annesi	grazia.annesi@cnr.it	National Research Council	Cosenza		Nothing to declare
**Italy**	Lucilla Parnetti	lucilla.parnetti@unipg.it	University of Perugia	Perugia		Nothing to declare
**Italy**	Micol Avenali	micol.avenali@mondino.it	University of Pavia	Pavia		Nothing to declare
**Italy**	Monica Gagliardi	monica.gagliardi@unicz.it	Magna Graecia University	Catanzaro		Nothing to declare
**Italy**	Tommaso Schirinzi	tommaso.schirinzi@opbg.net	University of Rome Tor Vergata	Rome		Nothing to declare
**Italy**	Caterina Galandra	caterina.galandra@mondino.it	IRCCS Mondino Foundation	Pavia		Nothing to declare
**Italy**	Anna De Rosa	anna.derosa1@unina.it	University of Naples Federico II	Naples		Nothing to declare
**Italy**	Rosangela Ferese	ferese.rosangela@gmail.com	IRCCS Neuromed	Pozzilli		Nothing to declare
**Italy**	Jolanda Buonocore	jolandabuonocore@gmail.com	Magna Graecia University	Catanzaro		Nothing to declare
**Italy**	Radha Procopio	radha.procopio@unicz.it	Magna Graecia University	Catanzaro		Nothing to declare
**Italy**	Ilaria Palmieri	ilaria.palmieri01@universitadipavia.it	IRCCS Mondino Foundation	Pavia		Nothing to declare
**Italy**	Michele Terzaghi	michele.terzaghi@mondino.it	University of Pavia	Pavia		Nothing to declare
**Italy**	Paola Dimartino	paola.dimartino@unipv.it	University of Pavia	Pavia		Nothing to declare
**Italy**	Roberta Bovenzi	roberta.bovenzi@gmail.com	University of Tor Vergata	Rome		Nothing to declare
**Italy**	Maxine Paige-Pritchett	maxine.paigepritchett01@universitadipavia.it	Università di Pavia	Pavia		Nothing to declare
**Japan**	Manabu Funayama	funayama@juntendo.ac.jp	Juntendo University	Tokyo		Nothing to declare
**Japan**	Nobutaka Hattori	nhattori@juntendo.ac.jp	Juntendo University faculty of medicine	Tokyo		Nothing to declare

**Japan**	Tomotaka Shiraishi	shiraishito@jikei.ac.jp	Jikei University School of Medicine	Tokyo		Nothing to declare
**Japan**	Kensuke Daida	k-daida@juntendo.ac.jp	Juntendo University	Bunkyō		Nothing to declare
**Kazakhstan**	Altynay Karimova	neurokz@mail.ru	Institute of Neurology and Neurorehabilitation	Almaty		Nothing to declare
**Kazakhstan**	Gulnaz Kaishibayeva	Neurology_institute@mail.ru	Institute of Neurology and Neurorehabilitation	Almaty		Nothing to declare
**Kazakhstan**	Aigerim Utegenova	87012226598@mail.ru	West Kazakhstan Marat Ospanov State Medical University	Aktobe		Nothing to declare
**Kazakhstan**	Vadim Akhmetzhanov	vadiman49@gmail.com	Medline medical center	Astana		Nothing to declare
**Kazakhstan**	Seitzhan Aidarov	seitzhanaidarov@gmail.com	National Center for Neurosurgery	Astana		Nothing to declare
**Kazakhstan**	Tautanova Raushan	raushanat@gmail.com	Astana Medical University	Astana		Nothing to declare
**Kazakhstan**	Dinara Alzhanova	dinara.alzhanowa@yandex.ru	Astana Medical University	Astana		Nothing to declare
**Kazakhstan**	Zhanybek Myrzayev	myrzaevjanik@gmail.com	International University of Postgraduate Education	Almaty		Nothing to declare
**Kazakhstan**	Saltanat Abdraimova	abdraimovasaltanat06@gmail.com	South Kazakhstan Medical Academy	Shymkent		Nothing to declare
**Kazakhstan**	Nazira Zharkinbekova	nazirazhar@mail.ru	South Kazakhstan Medical Academy	Shymkent		South Kazakhstan Medical academy
**Kazakhstan**	Chingiz Shashkin	chingizshashkin@gmail.com	International Research Institute of Postgraduate Education	Almaty		Nothing to declare
**Kazakhstan**	Guzel Shiderova	guzelshiderova@gmail.com	Institute of Neurology and Neurorehabilitation	Almaty		Nothing to declare
**Kazakhstan**	Bagzhan Syzdykova	syzdykova-75@mail.ru	Astana Medical University	Astana		Nothing to declare

**Kazakhstan**	Aigul. P. Yermagambetova	aiguler72@mail.ru	West Kazakhstan Marat Ospanov Medical University	Aktobe		Nothing to declare
**Kazakhstan**	Alima A. Khamidulla	alima.khamidulla@gmail.com	West Kazakhstan Marat Ospanov Medical University	Aktobe		Nothing to declare
**Kazakhstan**	Zhanylsyn U.Urasheva	zhanylsyn.urasheva90@gmail.com	West Kazakhstan Marat Ospanov Medical University	Aktobe		Nothing to declare
**Kazakhstan**	Gulnar B. Kabdrakhmanova	nevrolog.ufa@gmail.com	West Kazakhstan Marat Ospanov Medical University	Aktobe		Nothing to declare
**Kazakhstan**	Talgat Khaibullin	htn2006@mail.ru	Semey Medical University	Semey		Nothing to declare
**Kazakhstan**	Altynay Talgatkyzy	neurokz@mail.ru	Semey Medical University	Semey		Nothing to declare
**Kazakhstan**	Akper Sagynysh	sagga133124@mail.ru	Multiple Sclerosis and Autoimmune Neurological Disorders Center at the Multidisciplinary City Hospital No. 1	Moscow		Nothing to declare
**Kazakhstan**	Sadenova Aigul	sadenova.97@mail.ru	Multidisciplinary City Hospital No. 1	Moscow		Nothing to declare
**Kenya**	Eunice Nyambane	Eunice.nyambane@aku.edu	Aga Khan University Nairobi	Nairobi		Nothing to declare
**Kyrgyzstan**	Cholpon Shambetova	cholpon.shambetova@gmail.com	Kyrgyz State Medical Academy	Bishkek		Nothing to declare
**Kyrgyzstan**	Bermet Nurbekova	nurbekovabermet@gmail.com	I.K. Akhunbaev Kyrgyz State Medical Academy (KSMA)	Bishkek		Nothing to declare
**Kyrgyzstan**	Nurayim Beishembieva	Nuraiymbeishenbieva@gmail.com	I.K. Akhunbaev Kyrgyz State Medical Academy (KSMA)	Bishkek		Nothing to declare
**Kyrgyzstan**	Nargiza Atambekova	nargiza.atambekova94@gmail.com	I.K. Akhunbaev Kyrgyz State Medical Academy (KSMA), Clinical Hospital of Emergency Medical Care	Bishkek		Nothing to declare

**Kyrgyzstan**	Zhumagul Osmonova	jnprof05@gmail.com	Kyrgyz State Medical Institute of postgraduate training and continuous education named after S.B. Daniyarov	Bishkek		Nothing to declare
**Kyrgyzstan**	Maatali Abdimanap uulu	aliteshebaev@gmail.com	Osh State University, Osh City Clinical Hospital	Osh		Nothing to declare
**Kyrgyzstan**	Aisuikum Abdumalikova	aaisuikum@gmail.com	I.K. Akhunbaev Kyrgyz State Medical Academy (KSMA), Osh City Clinical Hospital	Osh		Nothing to declare
**Kyrgyzstan**	Adylbek Iusupov	adylbekusupov@gmail.com	I.K. Akhunbaev Kyrgyz State Medical Academy (KSMA), Osh City Clinical Hospital	Osh		Nothing to declare
**Kyrgyzstan**	Aliia Mukhanova	aliyucha11lin@mail.ru	International Higher School of Medicine	Bishkek		Nothing to declare
**Kyrgyzstan**	Zhyldyz Imanalieva	imanalieva2016kgz@gmail.com	I.K. Akhunbaev Kyrgyz State Medical Academy (KSMA), "Aqua Lab" LLC	Bishkek		Nothing to declare
**Kyrgyzstan**	Gulmira Zhamilova	gulmrazh@gmail.com	Clinical Hospital of the Presidential Administration of the Kyrgyz Republic	Bishkek		Nothing to declare
**Kyrgyzstan**	Shokhista Artyshova	Shohartyshova@gmail.com	Clinical Hospital of the Presidential Administration of the Kyrgyz Republic	Bishkek		Nothing to declare
**Kyrgyzstan**	Sabina Baltabaeva	Sabinabaltabaeva95@gmail.com	I.K. Akhunbaev Kyrgyz State Medical Academy (KSMA), Medcenter.kg" clinic	Bishkek		Nothing to declare
**Kyrgyzstan**	Mariia Rekaeva	rekaevamaria@gmail.ru	I.K. Akhunbaev Kyrgyz State Medical Academy (KSMA), "Unimed" Clinic	Bishkek		Nothing to declare
**Kyrgyzstan**	Aziza Dalbaeva	doctordalbaevaaziza89@gmail.com	National Hospital	Bishkek		Nothing to declare
**Kyrgyzstan**	Anna Khamzina	anna_hamzina@mail.ru	I.K. Akhunbaev Kyrgyz State Medical Academy (KSMA), "Unimed" Clinic	Bishkek		Nothing to declare
**Kyrgyzstan**	Saule Temirbaeva	lioness_bi@mail.ru	National Hospital	Bishkek		Nothing to declare
**Kyrgyzstan**	Mukhammadiusuf	aratingo@mail.ru	Osh State University	Osh		Nothing to declare

	Abdykadyrov					
**Kyrgyzstan**	Zynan Ruslan uulu	ruslanovzynan@gmail.com	I.K. Akhunbaev Kyrgyz State Medical Academy (KSMA)	Bishkek		Nothing to declare
**Kyrgyzstan**	Nurbakyt Kadyrov	Nurbakyt 97 kn@mail.ru	Osh City Clinical Hospital	Osh		Nothing to declare
**Kyrgyzstan**	Batma Sattarova	sattarovabatma1@gmail.com	Batken Regional Hospital	Batken		Nothing to declare
**Kyrgyzstan**	Begimai Sovetbekova	Edbemchik@gmail.com	I.K. Akhunbaev Kyrgyz State Medical Academy (KSMA)	Bishkek		Nothing to declare
**Lebanon**	Fouad Khoury	Fouadmkhoury@outlook.com	Université Saint George - Hôpital national de neurologie et de neurochirurgie	Beirut		Nothing to declare
**Lithuania**	Gintare Baranauskiene	gintare.baranauskiene@mf.stud.vu.lt	Vilnius University	Vilnius		Nothing to declare
**Luxembourg**	Rejko Krüger	rejko.krueger@uni.lu	University of Luxembourg	Esch-sur-Alzette		Nothing to declare
**Luxembourg**	Patrick May	patrick.may@uni.lu	University of Luxembourg	Esch-sur-Alzette		Nothing to declare
**Madagascar**	Nomena Rasaholiarison	noux aulx@yahoo.com	Faculty of Medicine, University of Fianarantsoa	Fianarantsoa		Nothing to declare
**Malaysia**	Ai Huey Tan	tan.aihuey@ummc.edu.my	University of Malaya	Kuala Lumpur		Nothing to declare
**Malaysia**	Azlina Ahmad-Annuar	azlina aa@um.edu.my	University of Malaya	Kuala Lumpur		Nothing to declare
**Malaysia**	Mohamed Ibrahim Norlinah	norlinah@ppukm.ukm.edu.my	Universiti Kebangsaan Malaysia	Selangor		Nothing to declare
**Malaysia**	Nor Azian Abdul Murad	nor_azian@ppukm.ukm.edu.my	UKM Medical Molecular Biology Institute	Kuala Lumpur		Nothing to declare

**Malaysia**	Shahrul Azmin	shahrulazmin@hotmail.com	Universiti Kebangsaan Malaysia Medical Centre	Kuala Lumpur		Nothing to declare
**Malaysia**	Shen-Yang Lim	sylim@ummc.edu.my	University of Malaya	Kuala Lumpur		Nothing to declare
**Malaysia**	Wael Mohamed	waelmohamed@iium.edu.my	International Islamic University	Kuala Lumpur		Nothing to declare
**Malaysia**	Yi Wen Tay	tayyiwen96@um.edu.my	University of Malaya	Kuala Lumpur		Nothing to declare
**Malaysia**	Lim Kai-Shi	limkaishi612@gmail.com	University of Malaya	Kuala Lumpur		Nothing to declare
**Malaysia**	Azalea Tenerife Pajo	azaleapajo@gmail.com	University of Malaya	Kuala Lumpur		Nothing to declare
**Malaysia**	Chia Yuen Kang	cyk1118@gmail.com	Hospital Queen Elizabeth	Kota Kinabalu		Nothing to declare
**Malaysia**	Joshua Ooi Chin Ern	joshuaooichinern@gmail.com	Queen Elizabeth Hospital	Kota Kinabalu		Nothing to declare
**Malaysia**	Khairul Azmi Ibrahim	khairulazmi73@yahoo.com	HOSPITAL SULTANAH NUR ZAHIRAH KUALA TERENGGANU	KUALA TERENGGANU		Nothing to declare
**Malaysia**	Ahmad Shahir Bin Mawardi	sha ray@yahoo.com	Hospita Kuala Lumpur	Kuala Lumpur		Nothing to declare
**Malaysia**	Lim Thien Thien	thienthienlim@gmail.com	Island Hospital	Penang		Nothing to declare
**Malaysia**	Tzi Shin Toh	tzishintoh@gmail.com	University of Malaya	Kuala Lumpur		Nothing to declare
**Mexico**	Daniel Martinez-Ramirez	daniel.martinez-ramirez@tec.mx	Tecnologico de Monterrey	Monterrey		Nothing to declare
**Mexico**	Paula Reyes-Pérez	prreyesperez@liigh.unam.mx	Universidad Nacional Autónoma de México	Santiago de Querétaro		Global Parkinson's Genetics Program

**Mexico**	Alejandra Medina Rivera	amedina@liigh.unam.mx	Universidad Nacional Autónoma de México	Santiago de Querétaro		Nothing to declare
**Mexico**	Nancy Monroy Jaramillo	nancy.monroy@innn.edu.mx	Instituto Nacional de Neurología y Neurocirugía	Mexico City		Nothing to declare
**Mexico**	Nadia Alejandra Gandarilla Martinez	ngandarilla.neurologia@gmail.com	Centro Neurológico del Centro Médico ABC, Campus Santa Fe	Mexico City		Nothing to declare
**Mexico**	Ingrid Estrada-Bellmann	ingridestmann@hotmail.com	UNIVERSITY HOSPITAL “DR JOSE E GONZALEZ”	Monterrey		Nothing to declare
**Mexico**	Araliz Puente	aralizp@gmail.com	Hospital Ángeles Puebla	Puebla		Nothing to declare
**Mexico**	Ana Paula Angulo Arrieta	ana.anguloar@anahuac.mx	Hospital Ángeles Puebla, Universidad Anáhuac Puebla	Puebla		Nothing to declare
**Mexico**	Eugenia Morelos Figaredo	neuro.emf@gmail.com	ISSSTE Morelia	Morelia		Nothing to declare
**Mexico**	Karla Salinas Barboza	saurka24@gmail.com	HOSPITAL GENERAL DE MEXICO	Mexico City		Nothing to declare
**Mexico**	Dante Bernardo Oropeza Canto	danteoropeza2@hotmail.com	Hospital Angeles de Puebla	Puebla		Nothing to declare
**Mexico**	Mayela Rodríguez-Violante	mrodriguez@innn.edu.mx	Instituto Nacional de Neurología y Neurocirugía	Mexico City		Nothing to declare
**Mexico**	Ana Jimena Hernández-Medrano	anajimenahdz@gmail.com	Instituto Nacional de Neurología y Neurocirugía Manuel Velasco Suárez	Mexico City		Nothing to declare

**Mexico**	Amin Cervantes-Arriaga	acervantes@innn.edu.mx	Instituto Nacional de Neurología y Neurocirugía	Mexico City		Nothing to declare
**Mexico**	Edith Janeth Gaspar Martínez	edith.gaspar@protonmail.com	Universidad Nacional Autónoma de México	Santiago de Querétaro		Nothing to declare
**Mexico**	Alejandra E-Ruiz-Contreras	aleruiz@unam.mx	Universidad Nacional Autonoma de Mexico	Mexico City		Nothing to declare
**Mexico**	Alejandra Lázaro-Figueroa	lazfigal@gmail.com	National Autonomous University of Mexico	Mexico City		Nothing to declare
**Mexico**	Sarael Alcauter	Blvd. Juriquilla 3001. 76230	Universidad Nacional Autónoma de México	Mexico City		Nothing to declare
**Mexico**	Edwin Roberto Ramírez-Benítez	edwin.ramirez@innn.edu.mx	Instituto Nacional de Neurología y Neurocirugía	Mexico City		Nothing to declare
**Mexico**	Karina Cruz-Santillán	karina.cruz@innn.edu.mx	Instituto Nacional de Neurología y Neurocirugía	Mexico City		Nothing to declare
**Mexico**	Rodolfo Solís-Vivanco	rsolis@innn.edu.mx	Instituto Nacional de Neurología y Neurocirugía	Mexico City		Nothing to declare
**Mongolia**	Bayasgalan Tserensodnom	bayasgalan.ts@mnums.edu.mn	Mongolian National University of Medical Sciences	Ulaanbaatar		Nothing to declare
**Mongolia**	Khosbayar Tulgaa	khosbayar.t@mnums.edu.mn	Mongolian National University of Medical Sciences	Ulaanbaatar		Nothing to declare
**Mongolia**	Oyujin Ulziibaatar	ou252@nyu.edu	Mongolian National University of Medical Sciences	Ulaanbaatar		Nothing to declare

**Morocco**	Ahmed Bouhouche	a.bouhouche@um5r.ac.ma	Specialties Hospital, CHU Ibn Sina	Rabat		Nothing to declare
**Morocco**	Mossafa Hossain	hossein.mossafa@gmail.com	Clinique OCEANIC	CASABLANCA		Nothing to declare
**Nepal**	Rajeev Ojha	rajeevnet@hotmail.com	Tribhuvan University	Kirtipur		Nothing to declare
**Netherlands**	Wilma Van De Berg	wdj.vandeberg@amsterdamumc.nl	Vanderbilt University Medical Center	Amsterdam		Nothing to declare
**Netherlands**	Bas Bleom	bas.bloem@radboudumc.nl	Radboud University	Nijmegen		Nothing to declare
**Netherlands**	Bart Van De Warrenburg	bart.vandewarrenburg@radboudumc.nl	Radboud University Medical Center	Nijmegen		Nothing to declare
**Netherlands**	Lisette Charbonnier	l.charbonnier@lumc.nl	Brain Research and Innovation Center	Amsterdam		Nothing to declare
**New Zealand**	Tim J. Anderson	tim.anderson@otago.ac.nz	University of Otago	Dunedin		Heath Research Council of New Zealand; Ministry of Business Innovation and Employment (New Zealand), Neurological Foundation of New Zealand
**New Zealand**	Toni L. Pitcher	toni.pitcher@nzbri.org	University of Otago	Dunedin		Funding - Health Research

						Council of New Zealand
**New Zealand**	Daniel Jeremy Myall	daniel.myall@nzbri.org	New Zealand Brain Research Institution	Christchurch		Nothing to declare
**New Zealand**	John C. Dalrymple-Alford	john.dalrymple-alford@canterbury.ac.nz	University of Canterbury	Christchurch		Nothing to declare
**New Zealand**	Joseph Donnelly	JDonnelly@adhb.govt.nz	Te Whatu Ora - Health New Zealand Auckland, New Zealand	Auckland		Nothing to declare
**Nigeria**	Arinola Sanyaolu	asanyaolu@unilag.edu.ng	University of Lagos	Lagos		Nothing to declare
**Nigeria**	Njideka Okubadejo	nokubadejo@unilag.edu.ng	University of Lagos	Lagos		Michael J Fox Foundation; Tertiary Education Trust Fund (TETFUND) National Research Fund
**Nigeria**	Oluwadamilola Ojo	oluojo@unilag.edu.ng	College of Medicine of the University of Lagos	Lagos		Nothing to declare
**Nigeria**	Simon Izuchukwu Ozomma	simonozomma@yahoo.com	University of Calabar Teaching Hospital	Calabar		Nothing to declare
**Nigeria**	Kolawole Wahab	wahab.kw@unilorin.edu.ng	University of Ilorin	Ilorin		Nothing to declare
**Nigeria**	Oladunni Abiodun	oladunnidare@yahoo.com	General Hospital	Lagos		Nothing to declare
**Nigeria**	Sani Abubakar	saabubakar@abu.edu.ng	Ahmadu Bello University	Kaduna State		Nothing to declare
**Nigeria**	Fatimah Abdulai	fatimaabdulai32@yahoo.com	University of Abuja Teaching Hospital	Gwagwalada		Nothing to declare

**Nigeria**	Charles Achoru	charlesachoru82@gmail.com	Jos University Teaching Hospital	Jos		Nothing to declare
**Nigeria**	Osigwe Agabi	oagabi@unilag.edu.ng	College of Medicine, University of Lagos	Lagos		Nothing to declare
**Nigeria**	Uchechi Agulanna	ucheagulanna@gmail.com	Lagos University Teaching Hospital	Lagos		Nothing to declare
**Nigeria**	Rufus Akinyemi	roakinyemi@com.ui.edu.ng	Neuroscience and Ageing Research Unit, Institute for Advanced Medical Research and Training, College of Medicine, University of Ibadan	Ibadan		Nothing to declare
**Nigeria**	Wemimo Alaofin	awalaofin@gmail.com	University of Ilorin	Ilorin		Nothing to declare
**Nigeria**	Ifeyinwa Ani-Osheku	i.ani-osheku@nileuniversity.edu.ng	Asokoro District Hospital	Abuja		Nothing to declare
**Nigeria**	Roosevelt Anyanwu	ranyanwu@unilag.edu.ng	College of Medicine, University of Lagos	Lagos		Nothing to declare
**Nigeria**	Cyril Erameh	cyrilerameh@isth.com.ng	Irrua Specialist Teaching Hospital	Ilorin		Nothing to declare
**Nigeria**	Daniel Ezuduemoih	danpasezudu@gmail.com	Lagos University Teaching Hospital	Lagos		Nothing to declare
**Nigeria**	Abdullahi Ibrahim	dribrahimbichi@gmail.com	Federal University of Health Sciences Teaching Hospital	Azare		Nothing to declare
**Nigeria**	Erica Ikwenu	ikwenuerica@gmail.com	Lagos University Teaching Hospital	Lagos		Nothing to declare
**Nigeria**	Frank Imarhiagbe	frank.imarhiagbe@uniben.edu	University of Benin	Benin City		Nothing to declare
**Nigeria**	Ismaila Ishola	iishola@unilag.edu.ng	College of Medicine, University of Lagos	Lagos		Nothing to declare
**Nigeria**	Emmanuel Iwuozo	emmanuel.iwuozo@npmcn.edu.ng	Benue State University	Makurdi		Nothing to declare
**Nigeria**	Morenikeji Komolafe	morenikeji.komolafe@oauife.edu.ng	Obafemi Awolowo University	Ile-Ife		Nothing to declare

**Nigeria**	Alero Nnama	ibifurolove@gmail.com	University of Port Harcourt Teaching Hospital	Port Harcourt		Nothing to declare
**Nigeria**	Paul Nwani	po.nwani@unizik.edu.ng	Nnamdi Azikiwe University Teaching Hospital	Nnewi		Nothing to declare
**Nigeria**	Franscisca Nwaokorie	fnwaokorie@unilag.edu.ng	College of Medicine, University of Lagos	Lagos		Nothing to declare
**Nigeria**	Ernest Nwazor	ernestnwazor@yahoo.com	Rivers State University Teaching Hospital	Port Harcourt		Nothing to declare
**Nigeria**	Yahaya Obiabo	obiabo.yahaya@fuhso.edu.ng	Federal University of Health Sciences	Otukpo		Nothing to declare
**Nigeria**	Nkechi Obianozie	nkechiobian@gmail.com	University of Abuja Teaching Hospital	Gwagwalada		Nothing to declare
**Nigeria**	Olanike Odeniyi	nikeodeniyi@lagosstate.gov.ng	General Hospital	Lagos		Nothing to declare
**Nigeria**	Francis Odiase	francisodiase2000@hotmail.com	University of Benin	Benin City		Nothing to declare
**Nigeria**	Ewere Marie Ogbimi	eweremarie@gmail.com	Delta State University	Abraka		Nothing to declare
**Nigeria**	Adebimpe Ogunmodede	bimpefunmilola@yahoo.com	Federal Medical Center	Owo		Nothing to declare
**Nigeria**	Francis Ojini	fojini@unilag.edu.ng	University of Lagos	Lagos		Nothing to declare
**Nigeria**	Rashidat Olanigan	rolanigan@hotmail.com	Lagos State University Teaching Hospital	Ikeja		Nothing to declare
**Nigeria**	Adedunni Olusanya	dedunolusanya@gmail.com	College of Medicine, University of Lagos & R-Jolad Hospital	Lagos		Nothing to declare
**Nigeria**	Chiamaka Okereke	chiamakaokereke@unth.edu.ng	University of Nigeria Teaching Hospital	Ituku-Ozalla		Nothing to declare
**Nigeria**	Gerald Onwuegbuzie	gerald.onwuegbuzie@uniabuja.edu.ng	University of Abuja	Abuja		Nothing to declare
**Nigeria**	Godwin Osaigbovo	osaigbovog@unijos.edu.ng	Jos University Teaching Hospital	Jos		Nothing to declare

**Nigeria**	Nosakhare Osemwegie	nosakhare.osemwegie@uniport.edu.ng	University of Port Harcourt	Port Harcourt		Nothing to declare
**Nigeria**	Olajumoke Oshinaike	olufemi.oshinaike@lasucom.edu.ng	Lagos State University College of Medicine	Ikeja		Nothing to declare
**Nigeria**	Lukman Owolabi	olfemi.med@buk.edu.ng	Bayero University Kano	Kano		Nothing to declare
**Nigeria**	Raymond Owolabi	owolabiray@yahoo.com	Federal Medical Center	Owo		Nothing to declare
**Nigeria**	Shyngle Oyakhire	soyakhire@nationalhospital.gov.ng	National Hospital	Abuja		Nothing to declare
**Nigeria**	Fadimatu Sa'Ad	falmatasaad16@gmail.com	Federal Teaching Hospital	Gombe		Nothing to declare
**Nigeria**	Funmilola Taiwo	loleneuro@gmail.com	University College Hospital	Ibadan		Nothing to declare
**Nigeria**	Francisca Nwaokorie	fnwaokorie@unilag.edu.ng	University of Lagos	Lagos	Lagos	Nothing to declare
**Norway**	Lasse Pihlstrøm	lasse.pihlstrom@medisin.uio.no	Oslo University Hospital	Oslo		Southeastern Regional Health Authority, Norway and Michael J. Fox Foundation
**Norway**	Manuela Tan	manuela.tan@medisin.uio.no	Oslo University Hospital	Oslo		Southeastern Norway Regional Health Authority Norway and Michael J. Fox Foundation

**Norway**	Ingeborg Haugesag Lie	i.h.lie@studmed.uio.no	Oslo University Hospital	Oslo		Nothing to declare
**Norway**	Jodi Maple-Grødem	jodi.maple@uis.no	Stavanger University Hospital	Stavanger		Nothing to declare
**Norway**	Solveig E J Dalbro	soejac@ous-hf.no	Oslo University Hospital	Oslo		Nothing to declare
**Norway**	Ellen Hoven Maurtveten	emaurtve@ous-hf.no	Oslo University Hospital	Oslo		Nothing to declare
**Pakistan**	Shoaib Ur-Rehman	dr.shoaib@ustb.edu.pk	University of Science and Technology Bannu	Bannu		Nothing to declare
**Pakistan**	Mohamed Nour	man.nour22@gmail.com	Razi Hospital	Rawalpindi		Nothing to declare
**Peru**	Mario Cornejo-Olivas	mario.cornejo.o@incngen.org.pe	Universidad Cientifica del Sur	Lima		Michael J. Fox Foundation for Parkinson's Research and Aligning Science Across Parkinson's Initiative
**Peru**	Nicanor Mori Quispe	nicanor.mori@unmsm.edu.pe	Hospital Nacional Daniel A. Carrión	Lima		Nothing to declare
**Peru**	JULIA ESTHER RIOS PINTO	d.jrios@ms.upla.edu.pe	UNIVERSIDAD PERUANA LOS ANDES	Huancayo		Nothing to declare

**Peru**	Maryenela Illanes-Manrique	maryenela.illanes@atlanticfellows.org	Instituto Nacional de Ciencias Neurologicas	Lima		Nothing to declare
**Peru**	Cintia Margoth Armas Puente	Cintiamargoth@gmail.com	Instituto Nacional de Ciencias Neurológicas	Lima		Nothing to declare
**Peru**	Torres Luis	torresramirezl@hotmail.com	Instituto Nacional de Ciencias Neurologicas	Lima		Nothing to declare
**Philippines**	Maria Leila Doquenia	marialeiladoquenia@gmail.com	Metropolitan Medical Center	Manila		Nothing to declare
**Philippines**	Raymond Rosales	rlrosales@ust.edu.ph	Metropolitan Medical Center	Manila		Nothing to declare
**Philippines**	Gerard Saranza	gerardsaranza@gmail.com	Chong Hua Hospital	Cebu		Nothing to declare
**Poland**	Agata Gajos	agata.gajos@umed.lodz.pl	Medical University of Lodz	Lodz		Nothing to declare
**Russia**	Elena Iakovenko	helenaabracham@gmail.com	Research Center of Neurology	Moscow		Nothing to declare
**Russia**	Anna Gareeva	annagareeva@yandex.ru	Ufa Federal Research Center	Ufa		Nothing to declare
**Russia**	Gulnara Akhmadeeva	nevrolog.ufa@gmail.com	Ufa Scientific Center	Ufa		Nothing to declare
**Russia**	Irina Gilyazova	gilyasova_irina@mail.ru	Russian Academy of Sciences / Bashkir State Medical University	Ufa		Nothing to declare
**Saudi Arabia**	Bashayer Al Mubarak	bal-mubarak@kfshrc.edu.sa	King Faisal Specialist Hospital and Research Center	Riyadh		Nothing to declare
**Saudi Arabia**	Muhammad Umair	umairmu@ngha.med.sa	King Abdullah International Medical Research Center	Jeddah		Nothing to declare
**Saudi Arabia**	Nada Abdullah Altassan	naltassan@kfshrc.edu.sa	King Faisal Specialist Hospital and Research Center	Riyadh		Nothing to declare

**Serbia**	Nataša Dragašević Mišković	ntdragasevic@gmail.com	Neurology Clinic, University Clinical Center of Serbia	Belgrade		Nothing to declare
**Serbia**	Andona Milovanović	andona8@gmail.com	Neurology Clinic, University Clinical Center of Serbia	Belgrade		Nothing to declare
**Singapore**	Eng-King Tan	tan.eng.king@singhealth.com.sg	National Neuroscience Institute	Singapore		Singapore National Medical Research Council (MOH-OFLCG-000207)
**Singapore**	Jia Nee Foo	jianee.foo@ntu.edu.sg	Nanyang Technological University	Singapore		Singapore National Medical Research Council (MOH-000559)
**Singapore**	Elaine Chew	elaine.chew@ntu.edu.sg	Nanyang Technological University	Singapore		Nothing to declare
**Slovenia**	Vesna Van Midden	vesna.van.midden@gmail.com	Ljubljana University Medical Centre	Ljubljana		Nothing to declare
**South Africa**	Ferzana Amod	ferzanaamo@ialch.co.za	University of KwaZulu-Natal	Durban		Nothing to declare
**South Africa**	Jonathan Carr	jcarr@sun.ac.za	University of Stellenbosch	Stellenbosch		Nothing to declare
**South Africa**	Soraya Bardien	sbardien@sun.ac.za	Stellenbosch University	Stellenbosch		National Research Foundation of South Africa[Grant Number 129249]

**South Africa**	Nikita Pillay	nikitasipi40@gmail.com	University of the Western Cape	Bellville		Nothing to declare
**South Africa**	Kathryn Step	kathrynstep@sun.ac.za	Stellenbosch University	Cape Town		Nothing to declare
**South Africa**	Riaan Van Coller	rvcoller@gmail.com	University of Pretoria	Pretoria		Nothing to declare
**South Korea**	Beomseok Jeon	brain@snu.ac.kr	Seoul National University Hospital	Seoul		Nothing to declare
**South Korea**	Yun Joong Kim	yunjkim@yuhs.ac	Yongin Severance Hospital	Seoul		Nothing to declare
**South Korea**	Jung Hwan Shin	neosjh2009@gmail.com	Seoul National University	Seoul		Nothing to declare
**South Korea**	Joowon Jang	jereiard@naver.com	Seoul National University	Seoul		Nothing to declare
**South Korea**	Jee-Young Lee	wieber04@snu.ac.kr	SMG-SNU Boramae Medical Center, College of Medicine Seoul National University	Seoul		Dr. Lee has received a research grant of National Research Foundation (NRF) funded by the Korean Government (MSIT), a multidisciplinary research grant-in-aid and a focused research grant-in-aid from the Seoul Metropolitan Government Seoul National

						University (SMG-SNU) Boramae Medical Center, and a research grant from Gemvax, a speaker honorarium from SK chemicals and Bial, a travel support from Ono, and a scientific advisory board of Regeners, Inc.
**South Korea**	Ryul Kim	arkrk86@snu.ac.kr	SMG-SNU Boramae Meidcal Center	Seoul		Nothing to declare
**South Korea**	HAN-Joon Kim	movement@snu.ac.kr	Seoul National University Hospital	Seoul		Nothing to declare
**Spain**	Esther Cubo	ecubo@ubu.es	Hospital Universitario Burgos	Burgos		Nothing to declare
**Spain**	Ignacio Alvarez	ignacio.alvafer@gmail.com	University Hospital Mutua Terrassa	Barcelona		Nothing to declare
**Spain**	Janet Hoenicka	janet.hoenicka@sjd.es	Institut de Recerca Sant Joan de Deu	Barcelona		Fondo de Investigación Sanitaria, Instituto Salud Carlos III, Grant PI019/00126
**Spain**	Katrin Beyer	kbeyer@igtp.cat	Research Institute Germans Trias i Pujol	Barcelona		Nothing to declare

**Spain**	Maria Teresa Periñan	t.perinantocino@qmul.ac.uk	Instituto de Biomedicina de Sevilla	Seville		Nothing to declare
**Spain**	Pau Pastor	pastorpau@gmail.com	University Hospital Germans Trias i Pujol	Barcelona		Nothing to declare
**Spain**	Ruben Fernandez-Santiago	ruben.fernandez.santiago@gmail.com	Hospital Clínic de Barcelona	Barcelona		Nothing to declare
**Spain**	Pilar Gómez Garre	mgomez-ibis@us.es	Instituto de Biomedicina de Sevilla	Seville		Nothing to declare
**Spain**	Pablo Mir	pmir@us.es	Instituto de Biomedicina de Sevilla	Seville		Nothing to declare
**Spain**	Mario Ezquerra	ezquerra@recerca.clinic.cat	FCRB-IDIBAPS	Barcelona		Nothing to declare
**Spain**	Celia Painous Marti	painous@clinic.cat	Hospital Clinic Barcelona	Barcelona		Nothing to declare
**Spain**	Lola J. Díaz-Feliz	lolaj.diaz@estudiante.uam.es	Fernando Pessoa University, San Roque Hospital	Las Palmas de Gran Canaria		Nothing to declare
**Spain**	José Matías Arbelo González	jose.arbelo@hospitalessanroque.com	Hospital Universitario San Roque Las Palmas/ Universidad Fernando Pessoa Canarias (UFPC)	Las Palmas de Gran Canaria		Nothing to declare
**Spain**	Juan Carlos Martínez Castrillo	jcmcastrillo@gmail.com	Hospital Ramón y Cajal	Madrid		Nothing to declare
**Spain**	Marina Mata	mmataal@yahoo.es	Hospital Universitario Infanta Sofia	Madrid		Nothing to declare
**Spain**	Oriol De Fabregues	odefabregues@gmail.com	Hospital Universitari Vall d'Hebron	Barcelona		Nothing to declare

**Spain**	Lydia Vela-Desojo	lvela61@gmail.com	Hospital Universitario Fundación Alcorcón	Madrid		Nothing to declare
**Spain**	Manuel Menendez Gonzalez	menendezgmanuel@uniovi.es	Hospital Universitario Central de Asturias	Oviedo		Nothing to declare
**Spain**	Yaroslau Compta	ycompta@clinic.cat	IDIBAPS / Hospital Clinic	Barcelona		Nothing to declare
**Spain**	Alicia Garrido	agarridop@clinic.cat	IDIBAPS-FCRB. Hospital Clinic Barcelona	Barcelona		Nothing to declare
**Spain**	Maria J Marti	mjmarti@clinic.cat	Hospital Clinic de Barcelona. Institut d'Investigacio Biomedica August Pi i Sunyer (IDIBAPS)	Barcelona		Nothing to declare
**Spain**	Almudena Sánchez-Gómez	alsanchez@clinic.cat	Hospital Clinic of Barcelona	Barcelona		Nothing to declare
**Spain**	Alexia T. Sánchez Reyes	investigacionUTM@ufpcanarias.es	Universidad Fernando Pessoa Canarias	Las Palmas de Gran Canaria		Nothing to declare
**Spain**	Laia Muñoz Llahuna	lmunozl@santpau.cat	IR Sant Pau	Barcelona		Nothing to declare
**Spain**	Joaquim Aumatell Escabies	jaumatelle@santpau.cat	IR SANT PAU	Barcelona		Nothing to declare
**Spain**	Javier Pagonabarraga Mora	jpagonabarraga@santpau.cat	IR SANT PAU	Barcelona		Nothing to declare
**Spain**	Ignacio Illán Gala	iillan@santpau.cat	IR SANT PAU	Barcelona		Nothing to declare
**Spain**	Esteban Muñoz	jemunoz@clinic.cat	Hospital Clínic de Barcelona	Barcelona		Nothing to declare
**Spain**	Manuela San	msaneufrasio-ibis@us.es	Instituto de Biomedicina de Sevilla	Sevilla		Nothing to declare

	Eufrasio Martínez					
**Spain**	Laura Muñoz Delgado	lmunoz-ibis@us.es	Instituto de Biomedicina de Sevilla	Sevilla		Nothing to declare
**Spain**	Rafael Díaz Belloso	rdbelloso-ibis@us.es	Instituto de Biomedicina de Sevilla	Sevilla		Nothing to declare
**Spain**	Sergio García Díaz	sgarcia5@us.es	Instituto de Biomedicina de Sevilla	Sevilla		Nothing to declare
**Spain**	Marta Bonilla Toribio	mbonilla-ibis@us.es	Instituto de Biomedicina de Sevilla	Sevilla		Nothing to declare
**Spain**	Dolores Buiza Rueda	dbuiza-ibis@us.es	Instituto de Biomedicina de Sevilla	Sevilla		Nothing to declare
**Spain**	Antonio Cristobal Luque Ambrosiani	acluque-ibis@us.es	Instituto de Biomedicina de Sevilla	Sevilla		Nothing to declare
**Spain**	Silvia Jesus Maestre	smaestre-ibis@us.es	Instituto de Biomedicina de Sevilla	Sevilla		Nothing to declare
**Spain**	Daniel Macías García	dmacias-ibis@us.es	Instituto de Biomedicina de Sevilla	Sevilla		Nothing to declare
**Spain**	Elena Ojeda Lepe	eojeda-ibis@us.es	Instituto de Biomedicina de Sevilla	Sevilla		Nothing to declare
**Spain**	Rocío Pineda Sánchez	rpineda-ibis@us.es	Instituto de Biomedicina de Sevilla	Sevilla		Nothing to declare

**Spain**	Ana Castellano Guerrero	acastellano-ibis@us.es	Instituto de Biomedicina de Sevilla	Sevilla		Nothing to declare
**Spain**	Astrid Daniela Adarmes Gómez	adarmes-ibis@us.es	Instituto de Biomedicina de Sevilla	Sevilla		Nothing to declare
**Spain**	Cristina Pérez Calvo	cperez6-ibis@us.es	Instituto de Biomedicina de Sevilla	Seville		Nothing to declare
**Spain**	Alejandro Salguero Oviedo	asalguero-ibis@us.es	Instituto de Biomedicina de Sevilla	Sevilla		Nothing to declare
**Spain**	Lorena Clavijo Jiménez	lclavijo-ibis@us.es	Instituto de Biomedicina de Sevilla	Sevilla		Nothing to declare
**Spain**	Natalia Lopez	natalia@gp2.org	Global Parkinson's Genetics Program (GP2)	Madrid		Nothing to declare
**Sri Lanka**	Gamini Karapitiya Pathirana	ddg@nhsl.health.gov.lk	National Hospital of Sri Lanka	Colombo		Nothing to declare
**Sri Lanka**	Ruwani Wijeyekoon	research@asn.lk	Association of Sri Lankan Neurologists	Colombo		Nothing to declare
**Sri Lanka**	Muhammed Saamir Mohideen	saamirms@gmail.com	National Hospital Galle	Galle		Nothing to declare
**Sri Lanka**	Manjula Caldera	dir.thanuradhapura@health.gov.lk	Teaching Hospital Anuradhapura	Anuradhapura		Nothing to declare
**Sri Lanka**	Arjuna Fernando	arjuna.fernando@pgim.cmb.ac.lk	National Hospital of Sri Lanka	Colombo		Nothing to declare
**Sri Lanka**	Darshana Sirisena	darshanasirisena@yahoo.com	CNTH Ragama	Ragama		Nothing to declare
**Sri Lanka**	Senaka Bandusena	senaka.bandusena@gmail.com	National Hospital of Sri Lanka	Colombo		Nothing to declare

**Sri Lanka**	Thashi Chang	thashi@clinmed.cmb.ac.lk	University of Colombo, Sri Lanka	Colombo		Nothing to declare
**Sri Lanka**	Darshana Dias Wijegunasinghe	wijegunasinghe@gmail.com	Colombo North Teaching hospital	Colombo		Nothing to declare
**Sri Lanka**	Gamini Pathirana	drpathirana@gmail.com	NHSL	Colombo		Nothing to declare
**Sri Lanka**	Bimsara Sajotha Nalin Senanayake	bimsarasena@gmail.com	Institution of Neurology, National Hospital of Sri Lanka	Colombo		Nothing to declare
**Sri Lanka**	Ajantha Keshavaraj	ajanthakeshavaraj@yahoo.com	Teaching Hospital Jaffna	Jaffna		Nothing to declare
**Sudan**	Sarah El-Sadig	Sarah.misbah@uofk.edu	Faculty of medicine university of Khartoum	Khartoum		Nothing to declare
**Sudan**	Inas Elsayed	inaselsayed25@gmail.com	Faculty of pharmacy University of Gezira	Wad Madani		Nothing to declare
**Sweden**	Kajsa Brolin	kajsa.atterling_brolin@med.lu.se	Lund University	Lund		Nothing to declare
**Sweden**	Per Svenningsson	per.svenningsson@ki.se	Karolinska Institute	Stockholm		Nothing to declare
**Sweden**	Maria Swanberg	maria.swanberg@med.lu.se	Lund University	Lund		Nothing to declare
**Switzerland**	Christiane Zweier	christiane.zweier@insel.ch	Inselspital Bern, University of Bern	Bern		Nothing to declare
**Switzerland**	Gerd Tinkhauser	gerd.tinkhauser@insel.ch	University Hospital Bern	Bern		Nothing to declare
**Switzerland**	Paul Krack	paul.krack@insel.ch	Inselspital Bern, University of Bern	Bern		Nothing to declare
**Switzerland**	Deborah Bartholdi	Deborah.Bartholdi@Insel.ch	University Hospital Bern	Bern		Nothing to declare
**Switzerland**	Jonas Ihle	Jonas.Ihle@triemli.zuerich.ch	Hopital Triemli Zürich	Zürich		Nothing to declare

**Taiwan**	Chin-Hsien Lin	chlin@ntu.edu.tw	National Taiwan University Hospital	Taipei City		Nothing to declare
**Taiwan**	Hsiu-Chuan Wu	serenahcwu@gmail.com	Chang Gung Memorial Hospital	Taoyuan City		Nothing to declare
**Taiwan**	Pin-Jui Kung	pinjuikung@gmail.com	National Taiwan University	Taipei City		Global Parkinson's Genetics Program
**Taiwan**	Ruey-Meei Wu	robinwu@ntu.edu.tw	National Taiwan University Hospital	Taipei City		Minister of Science and Technology, Taiwan Government; National Taiwan University; Parkinson foundation (USA); Michael J. Fox Foundation
**Taiwan**	Yihru Wu	yihruwu@adm.cgmh.org.tw	Chang Gung Memorial Hospital	Taoyuan City		Nothing to declare
**Taiwan**	Pin-Shiuan, Chen	calice2657@gmail.com	National Taiwan University Hospital	Taipei		Nothing to declare
**Tajikistan**	Ganieva Manizha	ganieva.manizha.79@mail.ru	Avicenna Tajik State Medical University	Dushanbe		Nothing to declare
**Tajikistan**	Maksudjon Isrofilov	dr.maks-55@mail.ru	Avicenna Tajik State Medical University	Dushanbe		Nothing to declare
**Tanzania**	Kigocha Okeng'o	okigocha@yahoo.com	Muhimbili National Hospital-Mloganzila	Dar es Salaam		Nothing to declare
**Tanzania**	Philip Adebayo	philip.adebayo@aku.edu	The Aga Khan University	Dar es Salaam		Nothing to declare
**Tunisia**	Rim Amouri	rim.amouri@rns.tn	Mongi Ben Hmida National Institute of Neurolog	Tunis		Nothing to declare

**Tunisia**	Samia Ben Sassi	dr.samiabensassi@gmail.com	Mongi Ben Hmida National Institute of Neurology	Tunis		Nothing to declare
**Tunisia**	Chokri Mhiri	mhiri.chokri@gmail.com	Habib Bourguiba University Hospital	Sfax		Nothing to declare
**Tunisia**	Nabli Fatnassi Fatma	fatmafatnassi@yahoo.fr	National institute Mongi Ben Hmida of Neurology	Tunis		Nothing to declare
**Tunisia**	Amine Rachdi	Dr.rachdi.amine@gmail.com	Mongi Ben Hamida institute of Neurology			Nothing to declare
**Tunisia**	Zakaria Saied	zakariasaied@hotmail.com	National Institute Mongi Ben Hamida of Neurology	Tunis		Nothing to declare
**Tunisia**	Mouna Ben Djebara	mouna.bendjebara@gmail.co m	Razi Hospital	Tunis		Nothing to declare
**Tunisia**	Rania Zouari	zouari.rania12@gmail.com	National institute of neurology mongi ben hmida	Tunis		Nothing to declare
**Turkey**	A. Nazlı Başak	nbasak@ku.edu.tr	Koç University	Istanbul		Kirac Foundation and Koc Univ.
**Turkey**	Özgür Öztop Çakmak	ooztop@ku.edu.tr	Koç University	Istanbul		Nothing to declare
**Turkey**	Sibel Ertan	fserta@istanbul.edu.tr	Koç University	Istanbul		Nothing to declare
**Turkey**	Rezzak Yilmaz	Rezzak.Yilmaz@ankara.edu.t r	University of Ankara	Ankara		RY received grants from Ankara University, and honoraria/con sultancy fees from Abbvie, Gen, Ali Raif, Abdi Ibrahim, and advisory board

						contribution from Gen, and Abbvie.
**Turkey**	Binnur Çelik	binnurcelik@hotmail.com	University of Ankara	Ankara		Nothing to declare
**Turkey**	Gençer Genç	gencerg@gmail.com	Şişli Etfal Training and Research Hospital, University of Health Sciences, İstanbul, TR	Istanbul		Nothing to declare
**Turkey**	Muhittin Cenk Akbostancı	akbostan@medicine.ankara.edu.tr	Private Practice	Ankara		Nothing to declare
**Turkey**	Basar Bilgic	bilgicb@gmail.com	Istanbul University, Faculty of Medicine	Istanbul		Nothing to declare
**Turkey**	Bedia Samanci	bediasamanci@gmail.com	Istanbul University, Faculty of Medicine	Istanbul		Nothing to declare
**Turkey**	Murat Emre	muratemre56@gmail.com	Istanbul university	Istanbul		Nothing to declare
**Turkey**	Haşmet Hanağasi	hasmet@yahoo.com	ISTANBUL FACULTY OF MEDICINE	Istanbul		Nothing to declare
**Turkey**	Aysegul Gunduz	draysegulgunduz@yahoo.com	Istanbul University-Cerrahpasa, Cerrahpasa Faculty of Medicine	Istanbul		Nothing to declare
**Turkey**	Gulcin Benbir Senel	gulcin.benbir@iuc.edu.tr	Istanbul University-Cerrahpasa, Cerrahpasa Faculty of Medicine	Istanbul		Istanbul University-Cerrahpasa, Cerrahpasa Faculty of Medicine, Department of Neurology, Sleep and Disorders Unit, Istanbul, Turkiye

**United Kingdom**	Alastair Noyce	a.noyce@qmul.ac.uk	Queen Mary University of London	London		Prof. Noyce reports grants from Parkinson's UK, Barts Charity, Cure Parkinson’s, NIHR, Innovate UK, Virginia Keiley benefaction, Alchemab, Aligning Science Across Parkinson’s and Michael J Fox Foundation. Consultancy and personal fees from Astra Zeneca, AbbVie, Profile, Roche, Biogen, UCB, Bial, Charco Neurotech, uMedeor and Britannia.
**United Kingdom**	Anette Schrag	A.schrag@ucl.ac.uk	University College London	London		NIHR UCL/H Biomedical Research Centre

**United Kingdom**	Anthony Schapira	a.schapira@ucl.ac.uk	University College London	London		ASAP
**United Kingdom**	Camille Carroll	camille.carroll@plymouth.ac.uk	University of Plymouth	Plymouth		C Carroll receives salary from University of Plymouth, University Hospitals Plymouth NHS Trust and National Institute of Health Research; she has received advisory, consulting, and/or lecture fees from AbbVie, Bial, Lundbeck, Global Kinetics, Britannia and Medscape, and research funding from Parkinson's UK, Edmond J Safra Foundation, National Institute of Health

						Research and Cure Parkinson’s
**United Kingdom**	Donald Grosset	donald.grosset@glasgow.ac.uk	University of Glasgow	Glasgow		Tracking Parkinson's (J-1101) is funded by Parkinson's UK
**United Kingdom**	Eleanor J. Stafford	e.stafford@ucl.ac.uk	University College London	London		Nothing to declare
**United Kingdom**	Henry Houlden	h.houlden@ucl.ac.uk	University College London	London		Nothing to declare
**United Kingdom**	Huw R Morris	h.morris@ucl.ac.uk	University College London	London		Dr Morris is employed by UCL. In the last 12 months he reports paid consultancy from Roche and Amylyx ; lecture fees/honoraria - BMJ, Kyowa Kirin, Movement Disorders Society. Research Grants from Parkinson’s UK, Cure Parkinson’s

						Trust, PSP Association, CBD Solutions, Drake Foundation, Medical Research Council, Michael J Fox Foundation. Dr Morris is a co-applicant on a patent application related to C9ORF72 - Method for diagnosing a neurodegenerative disease (PCT/GB2012/052140)
**United Kingdom**	John Hardy	j.hardy@ucl.ac.uk	University College London	London		Nothing to declare
**United Kingdom**	Kin Ying Mok	k.mok@ucl.ac.uk	Univeristy College London	London		Nothing to declare
**United Kingdom**	Mie Rizig	m.rizig@ucl.ac.uk	University College London	London		Nothing to declare
**United Kingdom**	Nicholas Wood	n.wood@ucl.ac.uk	University College London	London		ASAP- CRN

**United Kingdom**	Nigel Williams	williamsnm@cardiff.ac.uk	Cardiff University	Cardiff		Parkinson's UK
**United Kingdom**	Olaitan Okunoye	olaitan.okunoye.16@ucl.ac.uk	University College London	London		Nothing to declare
**United Kingdom**	Rauan Kaiyrzhanov	rauan.kaiyrzhanov.14@ucl.ac.uk	University College London	London		Nothing to declare
**United Kingdom**	Rimona Weil	r.weil@ucl.ac.uk	University College London	London		Nothing to declare
**United Kingdom**	Seth Love	seth.love@bristol.ac.uk	University of Bristol	Bristol		Medical Research Council (UK) MR/T018569/1
**United Kingdom**	Simona Jasaityte	simona.jasaityte@ucl.ac.uk	University College London	London		Nothing to declare
**United Kingdom**	Sumit Dey	s.dey@qmul.ac.uk	Queen Mary University of London	London		The Michael J. Fox Foundation for Parkinson's Research
**United Kingdom**	Spencer Finch	s.finch@qmul.ac.uk	Queen Mary University of London	London		Nothing to declare
**United Kingdom**	Valentina Escott-Price	escottpricev@cardiff.ac.uk	Cardiff University	Cardiff		Nothing to declare
**United Kingdom**	Hamin Lee	hamin.lee@ucl.ac.uk	St George's, University of London	London		Nothing to declare

**United Kingdom**	Roger Barker	rab46@cam.ac.uk	University of Cambridge	Cambridge		Nothing to declare
**United Kingdom**	Mina Ryten	mina.ryten@ucl.ac.uk	University College London	London		Nothing to declare
**United Kingdom**	Michele Hu	michele.hu@ndcn.ox.ac.uk	University of Oxford	Oxford		Nothing to declare
**United Kingdom**	Laura Parkkinen	laura.parkkinen@ndcn.ox.ac.uk	University of Oxford	Oxford		Nothing to declare
**United Kingdom**	Kailash Bhatia	k.bhatia@ucl.ac.uk	University College London	London		Nothing to declare
**United Kingdom**	Richard Walker	richard.walker@nhct.nhs.uk	Northumbria Healthcare at NHS Foundation Trust	Newcastle upon Tyne		Nothing to declare
**United Kingdom**	Steve Gentleman	s.gentleman@imperial.ac.uk	Imperial College London	London		Nothing to declare
**United Kingdom**	Thomas Warner	t.warner@ucl.ac.uk	University College London	London		Nothing to declare
**United Kingdom**	David Burn	david.burn@newcastle.ac.uk	Newcastle University	Newcastle upon Tyne		Nothing to declare
**United Kingdom**	Christian Lambert	christian.lambert@ucl.ac.uk	Imperial College London	London		Nothing to declare
**United Kingdom**	Caroline Williams-Gray	chm27@cam.ac.uk	University of Cambridge	Cambridge		Nothing to declare
**United Kingdom**	Deborah Attuah	deborah.attuah@yld.io	YLD	London		Nothing to declare

**United Kingdom**	Raquel Real	r.real@ucl.ac.uk	University College London	London		Nothing to declare
**United Kingdom**	Yen Tai	yen.tai@imperial.ac.uk	Imperial College London	London		Nothing to declare
**United Kingdom**	Alexandra Zirra	a.zirra@qmul.ac.uk	Queen Mary University of London	London		Nothing to declare
**United Kingdom**	Christopher M Morris	c.m.morris@ncl.ac.uk	Newcastle University	Newcastle upon Tyne		Nothing to declare
**United Kingdom**	Matilda Lily Fenn	matildafenn1@gmail.com	University College London	London		Nothing to declare
**United Kingdom**	Andrew C Robinson	andrew.c.robinson@manchester.ac.uk	The University of Manchester	Manchester		Nothing to declare
**United Kingdom**	Lesley Yue Wu	lesleywu13@gmail.com	University College London	London		Nothing to declare
**United Kingdom**	Tessa Du Toit	tessa.toit.23@ucl.ac.uk	UCL	Londo		Nothing to declare
**United Kingdom**	Joshua Luc Isherwood Frost	joshua.frost@ucl.ac.uk	UCL Queen Square Institute of Neurology	London		Nothing to declare
**United Kingdom**	Federico Roncaroli	federico.roncaroli@manchester.ac.uk	University of Manchester	Manchester		Nothing to declare
**United Kingdom**	Ashvin Kuri	a.kuri2@nhs.net	Queen Mary University of London	London		Nothing to declare

**United Kingdom**	Sheena Waters	s.waters@qmul.ac.uk	Queen Mary University of London	London		Nothing to declare
**United Kingdom**	Laura Smith	l.j.smith@qmul.ac.uk	Queen Mary University of London	London		Nothing to declare
**United Kingdom**	Eduardo De Pablo-Fernández	e.depablofernandez@qmul.ac.uk	Queen Mary University of London	London		Nothing to declare
**United Kingdom**	Anisa Shahid	a.j.shahid@qmul.ac.uk	Queen Mary University of London	London		Nothing to declare
**United Kingdom**	Cristina Simonet	c.simonet@qmul.ac.uk	Queen Mary University of London	London		Nothing to declare
**United Kingdom**	Charlotte Dore	c.dore.17@ucl.ac.uk	University College London	London		Nothing to declare
**United Kingdom**	Oiher Serrano-Asensio	oiher.serrano.22@ucl.ac.uk	University College London	London		Nothing to declare
**United Kingdom**	Marco Toffoli	m.toffoli@ucl.ac.uk	University College London	London		Nothing to declare
**United Kingdom**	Riona Fumi	riona.fumi.20@ucl.ac.uk	University College London, Institute of Neurology	London		Nothing to declare
**United Kingdom**	Brook Huxford	b.f.r.huxford@qmul.ac.uk	Queen Mary University of London	London		Nothing to declare
**United Kingdom**	Harneek Chohan	h.chohan@qmul.ac.uk	Queen Mary University of London	London		Nothing to declare

**United Kingdom**	Sophie Meyer	sophiemeyer165@gmail.com	Queen Mary University of London	London		Nothing to declare
**United Kingdom**	Laura Pérez-Carbonell	l.perezcarbonell@qmul.ac.uk	Queen Mary University London / Guy's and St Thomas' NHS Foundation Trust	London		Nothing to declare
**United Kingdom**	Solomiia Bandrivska	bandrivska.solomiia@gmail.com	University College London	London		Nothing to declare
**United Kingdom**	Saiesha Dindayal	saieshadindayal@rocketmail.com	University College London	London		Nothing to declare
**United Kingdom**	Charlotte Andrews	c.andrews@qmul.ac.uk	Queen Mary University of London	London		Nothing to declare
**United Kingdom**	Emily Navarro Jones	emily.navarro-jones.23@ucl.ac.uk	University College London	London		Nothing to declare
**United Kingdom**	Kamalesh Dey	k.dey@qmul.ac.uk	Queen Mary University of London	London		Nothing to declare
**United Kingdom**	Faiza Durrani	f.durrani@qmul.ac.uk	Centre for Preventive Neurology	London		Nothing to declare
**United Kingdom**	Marte Jensen	marte.jensen.18@ucl.ac.uk	University College London	London		Nothing to declare
**United Kingdom**	Yoana Kordovska	yoana.kordovska.22@ucl.ac.uk	University College London	London		Nothing to declare
**United Kingdom**	Jennifer Brady	Jennifer.brady@ucl.ac.uk	University College London	London		Nothing to declare
**United Kingdom**	Nolia Lumley	nolia.lumley@ucl.ac.uk	University College London	London		Nothing to declare

**United Kingdom**	Erika Lam	erika.lam@ucl.ac.uk	University College London	London		Nothing to declare
**United Kingdom**	Tatiana Georgiades	tatiana.georgiades.21@ucl.ac.uk	University College London	London		Nothing to declare
**United Kingdom**	Kawmadi Wasundera Gunawardena	kawmadi.gunawardena@ouh.nhs.uk	Oxford University Hospitals NHS Foundation Trust	Oxford		Nothing to declare
**Uruguay**	Víctor E. Raggio Risso	vraggio@fmed.edu.uy	Facultad de Medciina, Universidad de la República	Montevideo		Nothing to declare
**Uruguay**	Elena M.Dieguez	Dra.elenadieguez@gmail.com	Facultad de Medicina universidad de de la tepublica montevideo uruguay	Montevideo		Nothing to declare
**USA**	Lara M. Lange	lara.lange@nih.gov	Laboratory of Neurogenetics, National Institute on Aging/Institute of Neurogenetics, University of Luebeck	Bethesda	Maryland	Nothing to declare
**USA**	Angel Vinuela		University of Puerto Rico	San Juan		Nothing to declare
**USA**	Alyssa O'Grady	aogrady@michaeljfox.org	The Michael J. Fox Foundation for Parkinson's Research	New York	New York	Nothing to declare
**USA**	Andrew B Singleton	andy@gp2.org	Global Parkinson’s Genetics Program (GP2)	Bethesda	Maryland	Michael J Fox Foundation for Parkinson's disease Research and Aligning Science Across Parkinson's Initiative
**USA**	Andrew K. Sobering	andrew.sobering@uga.edu	Augusta University / University of Georgia Medical Partnership	Augusta	Georgia	Nothing to declare

**USA**	Bernadette Siddiqi	bsiddiqi@michaeljfox.org	The Michael J. Fox Foundation for Parkinson's Research	New York	New York	Nothing to declare
**USA**	Bradford Casey	bcasey@michaeljfox.org	The Michael J. Fox Foundation for Parkinson's Research	New York	New York	Nothing to declare
**USA**	Brian Fiske	bfiske@michaeljfox.org	The Michael J. Fox Foundation for Parkinson's Research	New York	New York	Nothing to declare
**USA**	Cabell Jonas	cabell.jonas@kp.org	Mid-Atlantic Permanente Medical Group	Bethesda	Maryland	Nothing to declare
**USA**	Carlos Cruchaga	cruchagac@wustl.edu	Washington University	St. Louis	Missouri	National Institutes of Health (R01AG044546 (CC), P01AG003991(CC, JCM), RF1AG053303 (CC), RF1AG058501 (CC), U01AG058922 (CC), RF1AG074007 (YJS)), the Chuck Zuckerberg Initiative (CZI), the Michael J. Fox Foundation (LI, CC), and the Department of Defense (LI-W81XWH2010849). The

						recruitment and clinical characterization of research participants at Washington University were supported by NIH P30AG066444 (JCM), P01AG03991(JCM), and P01AG026276(JCM).
**USA**	Caroline B. Pantazis	cpantazis@aligningscience.com	Coalition for Aligning Science	Bethesda	Maryland	Nothing to declare
**USA**	Claire Wegel	cwegel@iu.edu	Indiana University	Bloomington	Indiana	Nothing to declare
**USA**	Cornelis Blauwendraat	cblauwendraat@aligningscience.com	Aligning Science Across Parkinson's (ASAP)	Bethesda	Maryland	Nothing to declare
**USA**	Dan Vitale	dan@datatecnica.com	Data Tecnica	Bethesda	Maryland	D.V.'s participation in this project was part of a competitive contract awarded to Data Tecnica International LLC by the National Institutes of Health to

						support open science research.
**USA**	Deborah Hall	deborah_a_hall@rush.edu	Rush University	Chicago	Illinois	Nothing to declare
**USA**	Dena Hernandez	hernand@mail.nih.gov	National Institutes of Health	Bethesda	Maryland	Nothing to declare
**USA**	Ekemini Riley	eriley@parkinsonsroadmap.org	Coalition for Aligning Science	Washington	Washington	Nothing to declare
**USA**	Faraz Faghri	faraz@datatecnica.com	Data Tecnica	Bethesda	Maryland	F.F.'s participation in this research was supported in part by the Intramural Research Program of the NIH, National Institute on Aging (NIA), National Institutes of Health, Department of Health and Human Services; project number ZO1 AG000535, as well as the National Institute of Neurological

						Disorders and Stroke. F.F.'s participation in this project was part of a competitive contract awarded to Data Tecnica International LLC by the National Institutes of Health to support open science research.
**USA**	Geidy E. Serrano	geidy.serrano@bannerhealth.com	Banner Sun Health Research Institute	Sun City	Arizona	Banner Sun Health Research Institute Brain and Body Donation Program of Sun City, Arizona for the provision of human biological materials. The Brain and Body Donation Program has been supported by

						the National Institute of Neurological Disorders and Stroke (U24 NS072026 National Brain and Tissue Resource for Parkinson's Disease and Related Disorders), the National Institute on Aging (P30 AG19610 and P30AG07298 0, Arizona Alzheimer's Disease Center), the Arizona Department of Health Services (contract 211002, Arizona Alzheimer's Research Center), the Arizona Biomedical Research Commission

						(contracts 4001, 0011, 05-901 and 1001 to the Arizona Parkinson's Disease Consortium) and the Michael J. Fox Foundation for Parkinson's Research
**USA**	Hampton Leonard	hampton@datatecnica.com	Data Tecnica	Bethesda	Maryland	H.L.L.'s participation in this project was part of a competitive contract awarded to Data Tecnica International LLC by the National Institutes of Health to support open science research.
**USA**	Hirotaka Iwaki	hirotaka@datatecnica.com	Data Tecnica	Washington	Washington	H.I.'s participation in this project was part of a competitive

						contract awarded to Data Tecnica International LLC by the National Institutes of Health to support open science research.
**USA**	Honglei Chen	chenho19@msu.edu	Michigan State University	East Lansing	Michigan	NIH/DoD/Parkinson Foundation/MSU Foundation/Gibby vs. Parky Foundation - No COI to disclose
**USA**	Ignacio F. Mata	matai@ccf.org	Cleveland Clinic	Cleveland	Ohio	Funding from MJFF and NIH
**USA**	Ignacio Juan Keller Sarmiento	ignacio.sarmiento@northwestern.edu	Northwestern University	Evanston	Illinois	Nothing to declare
**USA**	Jared Williamson	Jared.C.Williamson@kp.org	Kaiser Permanente	Oakland	California	Nothing to declare
**USA**	Jonggeol Jeff Kim	Jonggeol.Kim@bcm.edu	Baylor College of Medicine	Bethesda	Maryland	Nothing to declare
**USA**	Joseph Jankovic	pdcmdc@bcm.edu	Baylor College of Medicine	Houston	Texas	Nothing to declare
**USA**	Joshua Shulman	joshua.shulman@bcm.edu	Baylor College of Medicine / Texas Children's Hospital	Houston	Texas	Collection of samples and

						data at Baylor College of Medicine was supported by Huffington Foundation.
**USA**	J Solle	jsolle@michaeljfox.org	The Michael J. Fox Foundation for Parkinson's Research	New York	New York	Nothing to declare
**USA**	Kaileigh Murphy	kmurphy@michaeljfox.org	The Michael J. Fox Foundation for Parkinson's Research	New York	New York	Nothing to declare
**USA**	Kamalini Ghosh Galvelis	kghosh@parkinson.org	Parkinson's Foundation	Princeton	New Jersey	Nothing to declare
**USA**	Karen Nuytemans	knuytemans@med.miami.edu	University of Miami Miller School of Medicine	Miami	Florida	This work has been supported by the American Parkinson Disease Association and the Margaret Q. Landenberger Research Foundation.
**USA**	Karl Kieburtz	karl.kieburtz@chet.rochester.edu	Beth Israel Deaconess Medical Center	Boston	Massachusetts	Nothing to declare
**USA**	Katerina Markopoulou	amarkopoulou@northshore.org	North Shore University Health System	Chicago	Illinois	Nothing to declare
**USA**	Kenneth Marek	kmarek@indd.org	Institute for Neurodegenerative Disorders	New Haven	Connecticut	Consultant for Michael J Fox Foundation, GE Healthcare, Roche, UCB,

						BIAL, Denali, Takeda, , Cerapsir, UCB, Biohaven, Neuron23, Aprinoia, Astellas, Calico, Genentech, Invicro
**USA**	Kristin S. Levine	kristin@datatecnica.com	Data Tecnica	Washington	Washingt on	K.S.L.'s participation in this project was part of a competitive contract awarded to Data Tecnica International LLC by the National Institutes of Health to support open science research.
**USA**	Lana M. Chahine	lanachahine@pitt.edu	University of Pittsburgh	Pittsburgh	Pennsylv ania	Nothing to declare
**USA**	Laura Ibanez	ibanezl@wustl.edu	Washington University	St. Louis	Missouri	Nothing to declare
**USA**	Laurel Screven	laurel@gp2.org	Global Parkinson's Genetics Program (GP2)	Bethesda	Maryland	Nothing to declare
**USA**	Lauren Ruffrage	lruffrage@uabmc.edu	University of Alabama at Birmingham	Birmingham	Alabama	Nothing to declare

**USA**	Lisa Shulman	lshulman@som.umaryland.edu	University of Maryland	Baltimore	Maryland	Nothing to declare
**USA**	Luca Marsili	marsilla@ucmail.uc.edu	University of Cincinnati	Cincinnati	Ohio	Nothing to declare
**USA**	Maggie Kuhl	mkuhl@michaeljfox.org	The Michael J. Fox Foundation for Parkinson's Research	New York	New York	Nothing to declare
**USA**	Marissa Dean	mndean@uabmc.edu	University of Alabama at Birmingham	Birmingham	Alabama	Dr. Dean is an investigator in studies funded by Abbvie, Inc., Hoffmann-La Roche, CHDI Foundation, Inc., Annexon, Inc., Retrophin, Inc, Neurocrine Biosciences, UniQure Biopharma B.V., Praxis Precision Medicines, Neuraly, Inc., Michael J. Fox Foundation for Parkinson's Research, and US Army Medical Research and

						Material Command (grant#W81XWH-18-1-0508). In addition, Dr. Dean receives support through the Huntington's Disease Society of American Centers of Excellence program.
**USA**	Mary B Makarious	mary@datatecnica.com	Data Tecnica	Bethesda	Maryland	M.B.M.'s participation in this project was part of a competitive contract awarded to Data Tecnica International LLC by the National Institutes of Health to support open science research.
**USA**	Mathew Koretsky	mat@datatecnica.com	Data Tecnica	Bethesda	Maryland	M.K.'s participation in this project was part of a

						competitive contract awarded to Data Tecnica International LLC by the National Institutes of Health to support open science research.
**USA**	Megan J. Puckelwartz	m.puckelwartz@northwestern.edu	Northwestern University	Chicago	Illinois	Nothing to declare
**USA**	Mike A. Nalls	mike@datatecnica.com	Data Tecnica	Bethesda	Maryland	M.A.N.'s participation in this project was part of a competitive contract awarded to Data Tecnica International LLC by the National Institutes of Health to support open science research. M.A.N. also currently serves as an advisor for Clover

						Therapeutics and Neuron23 Inc.
**USA**	Naomi Louie	nlouie@michaeljfox.org	The Michael J. Fox Foundation for Parkinson's Research	New York	New York	Nothing to declare
**USA**	Niccolò Emanuele Mencacci	niccolo.mencacci@northwestern.edu	Northwestern University	Evanston	Illinois	Nothing to declare
**USA**	Roger Albin	ralbin@med.umich.edu	Universit of Michigan	Ann Arbor	Michigan	P50NS123067; Parkinson's Foundation
**USA**	Roy Alcalay	rna2104@cumc.columbia.edu	Columbia University	New York	New York	Dr. Alcalay is funded by the Micharl J. Fox Foundation and the Parkinson's Foundation. He received consultation fees from Avrobio, Caraway, GSK, Merck, Sanofi, Ono Therapeutics and Takeda
**USA**	Ruth Walker	ruth.walker@mssm.edu	James J. Peters Veterans Affairs Medical Center	New York	New York	Research funding from the Department of Veterans Affairs (CSRD Merit Award CX002342),

						consulted for Teladoc, Inc. and received an honorarium from New York University (NYU) Medical Center.
**USA**	Sohini Chowdhury	schowdhury@michaeljfox.org	The Michael J. Fox Foundation for Parkinson's Research	New York	New York	Nothing to declare
**USA**	Sonya Dumanis	sdumanis@parkinsonsroadmap.org	Aligning Science Across Parkinson's	Washington	Washington	Nothing to declare
**USA**	Steven Lubbe	steven.lubbe@northwestern.edu	Northwestern University	Chicago	Illinois	Nothing to declare
**USA**	Tao Xie	txie@bsd.uchicago.edu	University of Chicago	Chicago	Illinois	Nothing to declare
**USA**	Tatiana Foroud	tforoud@iu.edu	Indiana University School of Medicine	Indianapolis	Indiana	The Michael J. Fox Foundation for Parkinson's Research
**USA**	Thomas Beach	thomas.beach@bannerhealth.com	Sun Health Research Institution	Sun City	Arizona	Banner Sun Health Research Institute Brain and Body Donation Program of Sun City, Arizona for the provision of human

						biological materials. The Brain and Body Donation Program has been supported by the National Institute of Neurological Disorders and Stroke (U24NS072026 National Brain and Tissue Resource for Parkinson's Disease and Related Disorders), the National Institute on Aging (P30 AG19610 and P30AG072980, Arizona Alzheimer's Disease Center), the Arizona Department of Health Services (contract

						211002, Arizona Alzheimer's Research Center), the Arizona Biomedical Research Commission (contracts 4001, 0011, 05-901 and 1001 to the Arizona Parkinson's Disease Consortium) and the Michael J. Fox Foundation for Parkinson's Research
**USA**	Todd Sherer	tsherer@michaeljfox.org	The Michael J Fox Foundation for Parkinson's Research	New York	New York	Nothing to declare
**USA**	Dana Lewis	dlewis@parkinsonsroadmap.org	Aligning Science Across Parkinson's	Baltimore	Maryland	Nothing to declare
**USA**	Shreya Menon	shreya.menon@gladstone.ucsf.edu	Gladstone Institutes	San Francisco	California	Nothing to declare
**USA**	Melissa Nirenberg	melissa.nirenberg@gmail.com	Icahn School of Medicine at Mount Sinai	New York	New York	Nothing to declare
**USA**	Spencer Grant	spencer.grant@nih.gov	National Institutes of Health	Bethesda	Maryland	Nothing to declare
**USA**	Shannon Ballard	shannon@datatecnica.com	Data Tecnica	Bethesda	Maryland	S.B.'s participation

						in this project was part of a competitive contract awarded to Data Tecnica International LLC by the National Institutes of Health to support open science research.
**USA**	Chad Shaw	cashaw@bcm.edu	Baylor College of Medicine	Houston	Texas	Nothing to declare
**USA**	Sidra Aslam	Sidra.Aslam@bannerhealth.com	Banner Health	Phoenix	Arizona	Nothing to declare
**USA**	Devin Sharp	dsnyder@aligningscience.com	Aligning Science Across Parkinson's	Vancouver	British Columbia	Nothing to declare
**USA**	Rachel Saunders-Pullman	Rachel.Saunders-Pullman@mountsinai.org	Icahn School of Medicine at Mount Sinai	New York	New York	Nothing to declare
**USA**	Michiko Kimura Bruno	michikobruno@gmail.com	The Queen's Medical Center	Honolulu	Hawaii	Nothing to declare
**USA**	Matt Farrer	m.farrer@ufl.edu	University of Florida College of Medicine	Gainesville	Florida	Nothing to declare
**USA**	Haydeh Payami	haydehpayami@uabmc.edu	The University of Alabama at Birmingham Heersink School of Medicine	Birmingham	Alabama	Nothing to declare
**USA**	Ryan Pfingst	rpfingst@michaeljfox.org	The Michael J Fox Foundation	New York	New York	Nothing to declare
**USA**	James B Leverenz	leverej@ccf.org	Cleveland Clinic	Cleveland	Ohio	Nothing to declare

**USA**	Elizabeth Disbrow	elizabeth.disbrow@lsuhs.edu	LSU Health Shreveport	Shreveport	Louisiana	Nothing to declare
**USA**	Debi Brooks	dbrooks@michaeljfox.org	The Michael J Fox Foundation	New York	New York	Nothing to declare
**USA**	Randy Schekman	rschekman@parkinsonsroadmap.org	University of California, Berkeley	Berkeley	California	Nothing to declare
**USA**	Un Kang	un.kang@nyulangone.org	NYU Grossman School of Medicine	New York	New York	Nothing to declare
**USA**	Zbigniew K. Wszolek	Wszolek.Zbigniew@mayo.edu	Mayo Clinic College of Medicine	Rochester	Minnesota	Nothing to declare
**USA**	Cyrus Zabetian	zabetian@uw.edu	VA Puget Sound Health Care System	Seattle	Washington	Nothing to declare
**USA**	Zach Chaney	zchaney@michaeljfox.org	The Michael J Fox Foundation	New York	New York	Nothing to declare
**USA**	Christine Swanson-Fischer	christine.swanson-fischer@nih.gov	National Institutes of Health	Rockville	Maryland	Nothing to declare
**USA**	Conor Hennessey	chennessey@michaeljfox.org	The Michael J Fox Foundation	New York	New York	Nothing to declare
**USA**	Cassandra Barrett	cbarrett@michaeljfox.org	The Michael J Fox Foundation	New York	New York	Nothing to declare
**USA**	Beate Ritz	britz@ucla.edu	University of California, Los Angeles	Los Angeles	California	Nothing to declare
**USA**	Bradley Boeve	bboeve@mayo.edu	Mayo Clinic	Rochester	Minnesota	Nothing to declare
**USA**	Ashley Rawls	ashley.rawls@neurology.ufl.edu	University of Florida College of Medicine	Gainesville	Florida	Nothing to declare
**USA**	Holly A. Shill	holly.shill@commonspirit.org	Barrow Neurological Institute	Phoenix	Arizona	Nothing to declare
**USA**	Erika Driver-Dunckley	driverdunckley.erika@mayo.edu	Mayo Clinic AZ	Scottsdale	Arizona	Nothing to declare
**USA**	Bruce A. Chase	bruce.chase@endeavorhealth.org	Endeavor Health (formerly NorthShore University Health System)	Skokie	Illinois	Nothing to declare

**USA**	Mahesh Padmanaban	mpadmanaban@uchicagomedicine.org	University of Chicago	Chicago	Illinois	Nothing to declare
**USA**	Thiago Peixoto Leal	PEIXOTT@ccf.org	Cleveland Clinic	Cleveland	Ohio	Thiago Peixoto Leal is supported by the National Institutes of Health (NIH) grant R01 1R01NS112499-01A1 and Veterans Affair Puget Sound Healthcare System grant 5I01ABX005978-2
**USA**	Owen A. Ross	ross.owen@mayo.edu	Mayo Clinic	Jacksonville	Florida	Nothing to declare
**USA**	Michael Rose	michael.rose@osumc.edu	The Ohio State University Medical Center	Columbus	Ohio	Nothing to declare
**USA**	Ariane Park	ariane.park@osumc.edu	The Ohio State University Medical Center	Columbus	Ohio	I have received grants or contracts from the Parkinson's Foundation, Michael J. Fox Foundation, Parkinson Study Group

						and honoraria for consultancy from Cerevel.
**USA**	Victoria Klee	victoria.klee@osumc.edu	The Ohio State University	Columbus	Ohio	Nothing to declare
**USA**	James C. Beck	jbeck@parkinson.org	Parkinson's Foundation	New York	New York	Nothing to declare
**USA**	Suzanne Judd	sejudd@uab.edu	UAB	Birmingham	Alabama	Nothing to declare
**USA**	Daniel Weintraub	daniel.weintraub@pennmedicine.upenn.edu	U. Pennsylvania	Philadelphia	Pennsylvania	Nothing to declare
**USA**	Vikas Kotagal	vikaskot@med.umich.edu	University of Michigan	Ann Arbor	Michigan	Nothing to declare
**USA**	Nicolaas I. Bohnen	nbohnen@med.umich.edu	University of Michigan	Ann Arbor	Michigan	Nothing to declare
**USA**	Prabesh Kanel	prabeshk@med.umich.edu	University of Michigan	Ann Arbor	Michigan	Nothing to declare
**USA**	Chatkaew Pongmala	pchatkae@med.umich.edu	University of Michigan	Ann Arbor	Michigan	Nothing to declare
**USA**	Erin Williams	erin.williams@vai.org	Van Andel Institute	Grand Rapids	Michigan	Nothing to declare
**USA**	Audrey Strongosky	strongosky.audrey@mayo.edu	Mayo Clinic Florida	Jacksonville	Florida	Nothing to declare
**USA**	Michael Henderson	michael.henderson@vai.org	Van Andel Institute	Grand Rapids	Michigan	Nothing to declare
**USA**	Daniel C. Rohrer	dan.rohrer@vai.org	Van Andel Institute	Grand Rapids	Michigan	Nothing to declare
**USA**	Alexander Blanski	alex.blanski@vai.org	Van Andel Research Institute	Grand Rapids	Michigan	Nothing to declare
**USA**	Christina Missler	christina.missler@vai.org	Van Andel Institute	Grand Rapids	Michigan	Nothing to declare
**USA**	Alyssa Johansson	alyssa.johansson@vai.org	Van Andel Institute	Grand Rapids	Michigan	Nothing to declare

**USA**	Felipe Duarte-Zambrano	felipe.dz.101@gmail.com	Cleveland Clinic	Cleveland	Ohio	Nothing to declare
**USA**	Gist Croft	gcroft@nyscf.org	The New York Stem Cell Foundation	New York	New York	Nothing to declare
**USA**	Lisa Voltolina	lvoltolina@nyscf.org	New York Stem Cell Foundation Reseach Institute	New York	New York	Nothing to declare
**USA**	Whitley Aamodt	whitley.aamodt@pennmedicine.upenn.edu	University of Pennsylvania	Philadelphia	Pennsylvania	Nothing to declare
**USA**	Stewart A Factor	sfactor@emory.edu	Emory University	Atlanta	Georgia	Nothing to declare
**USA**	Alberto J. Espay	Alberto.espay@uc.edu	University of Cincinnati	Cincinnati	Ohio	Nothing to declare
**USA**	Nabila Dahodwala	nabila.dahodwala@gmail.com	University of Pennsyvlania	Philadelphia	Pennsylvania	Nothing to declare
**USA**	Chantale Branson	cbranson@msm.edu	Morehouse School of Medicine	Atlanta	Georgia	Nothing to declare
**USA**	Emily Hill	hill2e9@ucmail.uc.edu	University of Cincinnati	Cincinnati	Ohio	Nothing to declare
**USA**	Krutika Patel	krutika.patel.co@gmail.com	New York Stem Cell Foundation	Denver	Colorado	Nothing to declare
**USA**	Shyamal Mehta	mehta.shyamal@mayo.edu	Mayo Clinic, Arizona	Scottsdale	Arizona	Nothing to declare
**USA**	Emily Waldo	waldoe@ccf.org	Cleveland Clinic	Cleveland	Ohio	Nothing to declare
**USA**	Miguel Inca Martinez	incam@ccf.org	Cleveland Clinic Foundation	Cleveland	Ohio	Nothing to declare
**USA**	Anne-Marie Wills	awills@mgh.harvard.edu	Massachusetts General Hospital	Boston	Massachusetts	Nothing to declare
**USA**	Ejaz A. Shamim	ejaz.a.shamim@kp.org	Kaiser Permanente, MidAtlantic Permanente Research Institute	Washington	Washington	Nothing to declare
**USA**	Charles H. Adler	cadler@mayo.edu	Mayo Clinic College of Medicine, Mayo Clinic Arizona	Scottsdale	Arizona	Nothing to declare

**USA**	Ileana Lorenzini	ileana.lorenzini@bannerhealth.com	Banner Sun Health Research Institute	Sun City	Arizona	Nothing to declare
**USA**	Peter Heutink	peter@gp2.org	Global Parkinson’s Genetics Program (GP2)	Pacifica	California	Nothing to declare
**USA**	Bernabé Bustos	bernabe.bustos@northwestern.edu	Northwestern University Feinberg School of Medicine	Chicago	Illinois	Nothing to declare
**USA**	Shalini Padmanabhan	spadmanabhan@michaeljfox.org	Michael J. Fox Foundation	New York City	New York	Nothing to declare
**USA**	Serena Fong	sfong@michaeljfox.org	Michael J. Fox Foundation	New York City	New York	Nothing to declare
**USA**	Vidhu Agarwal	mail2vidhu.alld@gmail.com	National Institute of Health	Bethesda	Maryland	Nothing to declare
**USA**	Nicole Kuznetsov	nicole.kuznetsov@nih.gov	National Institute of Health	Bethesda	Maryland	Nothing to declare
**USA**	Lietsel Jones	lietsel@datatecnica.com	Data Tecnica International	Bethesda	Maryland	Nothing to declare
**USA**	Julia Staisch	julia.staisch@ochsner.org	Ochsner Clinic Foundation	New Orleans	Louisiana	Nothing to declare
**USA**	Camilla Kilbane	Camilla.Kilbane@UHhospitals.org	University Hospitals Cleveland Medical Center	Cleveland	Ohio	Nothing to declare
**USA**	Erin Furr Stimming	erin.e.furr@uth.tmc.edu	The University of Texas Health Science Center at Houston	Houston	Texas	Nothing to declare
**USA**	Scott Norris	norriss@wustl.edu	Washington University in St. Louis	St. Louis	Missouri	Nothing to declare
**USA**	Erica Bell	erica.bell@osumc.edu	The Ohio State University Medical Center	Columbus	Ohio	Nothing to declare
**USA**	Natalia Chunga	natalia.chunga@lsuhs.edu	Louisiana State University Health Sciences Center at Shreveport	Shreveport	Louisiana	Nothing to declare
**USA**	Mohamed Elkasaby	Mohamed.Elkasaby@UHhospitals.org	University Hospitals Cleveland Medical Center/Case Western Reserve University	Cleveland	Ohio	Nothing to declare
**USA**	Shivika Chandra	Shivika.chandra@uth.tmc.edu	UTHealth-Houston	Houston	Texas	Nothing to declare

**USA**	Erin Foster	erfoster@wustl.edu	Washington University School of Medicine	St. Louis	Missouri	Nothing to declare
**USA**	Vanessa Hinson	Hinsonvk@musc.edu	MUSC	Charleston	South Carolina	Nothing to declare
**USA**	Scott A. Norris	norriss@wustl.edu	Washington University	St. Louis	Missouri	Nothing to declare
**USA**	Andrew Nader AMeri	ameri@musc.edu	MUSC	Charleston	South Carolina	Nothing to declare
**Vietnam**	Duan Nguyen	ndduan@huemed-univ.edu.vn	Hue University	Huế		Nothing to declare
**Vietnam**	Toan Nguyen		Hue University	Huế		Nothing to declare
**Vietnam**	NguyễN Thái Thuỳ Ngân	tngan1105@gmail.com	University Medical Center, Ho Chi Minh City	Ho Chi Minh		Nothing to declare
**Vietnam**	Ha Ngoc Le Uyen	uyen.hnl@umc.edu.vn	University Medical Center Ho Chi Minh city	Ho Chi Minh		Nothing to declare
**Vietnam**	Tai Ngoc Tran	tai.tn@umc.edu.vn	University Medical Center HCMC	Ho Chi Minh		Nothing to declare
**Vietnam**	Khang Vo	khang.vnc@umc.edu.vn	University Medical Center	Ho Chi Minh		Nothing to declare
**Vietnam**	Vinh Thanh Nguyen	thanh.nv5@umc.edu.vn	University Medical Center Ho Chi Minh City	Ho Chi Minh		Nothing to declare
**Vietnam**	Lam Hoang Son	thankinh@umc.edu.vn	University Medical Center Ho Chi Minh City	Ho Chi Minh City		Nothing to declare
**Zambia**	Masharip Atadzhanov	masharip.atadzhanov@gmail.com	University of Zambia	Lusaka		Nothing to declare
